# Tuning Regioselectivity in Cyclopolymerization through Carbene Ligand Size Modulation in Molybdenum Imido Alkylidene Catalysts

**DOI:** 10.1021/acs.macromol.5c03366

**Published:** 2026-01-22

**Authors:** Koushani Kundu, Severin Haid, Patrick Probst, Laura Stöhr, Philipp Hauser, Wolfgang Frey, Johannes Kästner, Michael R. Buchmeiser

**Affiliations:** † Institute of Polymer Chemistry, 9149 University of Stuttgart, Pfaffenwaldring 55, 70569 Stuttgart, Germany; ‡ Institute of Theoretical Chemistry, University of Stuttgart, Pfaffenwaldring 55, 70569 Stuttgart, Germany; § Institute of Organic Chemistry, University of Stuttgart, Pfaffenwaldring 55, 70569 Stuttgart, Germany; ∥ German Institutes of Textile and Fiber Research (DITF), Körschtalstr. 26, 73770 Denkendorf, Germany

## Abstract

Herein we report on the regioselectivity in cyclopolymerization of diethyl dipropargylmalonate (DEDPM) with a set of 22 different neutral and cationic molybdenum imido alkylidene catalysts bearing either a cyclic alkyl amino carbene (CAAC) or *N*-heterocyclic carbene (NHC) ligand of the general formulas Mo­(*N*-Ar)­(CHCMe_2_R)­(NHC)­(X)_2_ and [Mo­(*N*-Ar)­(CHCMe_2_R)­(NHC or CAAC)­(X)]^+^ [(BAr^F^)_4_]^−^ (R = C_6_H_5_, CH_3_; Ar = 2,6-Me_2_C_6_H_3_, 2,6-Cl_2_C_6_H_3_, 2,6-^i^Pr_2_C_6_H_3_, 3,5-Me_2_C_6_H_3_, 2-^t^Bu-C_6_H_4_, 2-CF_3_–C_6_H_4_, C_6_F_5_; X = OTf, OC_6_F_5_, OMes, OCH­(CF_3_)_2_, OC­(CH_3_)­(CF_3_)_2_, 2′,4′,6′,2″,4″,6″-hexamethylterphen-1-yloxy (OHMT), Br, Cl; NHC = 1,3-dimesitylimidazol-2-ylidene (IMes), 1,3-dimesityl-4,5-dihydroimidazol-2-ylidene (IMesH_2_), 1,3-diisopropylimidazol-2-ylidene (IiPr), 1,3-dicyclohexylimidazol-2-ylidene (ICy), 1,3-dimesityl-3,4,5,6-tetrahydropyrimidin-2-ylidene (6-Mes), 1,3,4-triphenyl-1,2,4-triazol-5-ylidene (TPT), 1-(2,6-diisopropylphenyl)-3,3,5,5-tetramethyltetrahydropyrrol-2-ylidene (CAAC-1)). While most complexes result in predominantly α-insertion, a few also exhibit β-insertion, with α-insertion-derived triads as low as 37%. The α-selectivity was found to be proportional to the steric demand of the monomer. The influence of the coordinated carbene ligand on the regioselectivity of insertion was investigated by combining experimental data with the results obtained from DFT-optimized structures. The study revealed that the degree of α-selectivity depends on the steric size of the NHC or CAAC ligand and on the partial charge on the oxygen atom of the ester group of the coordinated monomer. The correlation is explained by two additional molybdenum–oxygen (ester) coordinations, which stabilize relevant intermediates. A correlation fit with a satisfactory *R* ^2^ of 0.854 confirms these effects quantitatively and the model was verified by repeated k-fold cross-validation.

## Graphical abstract

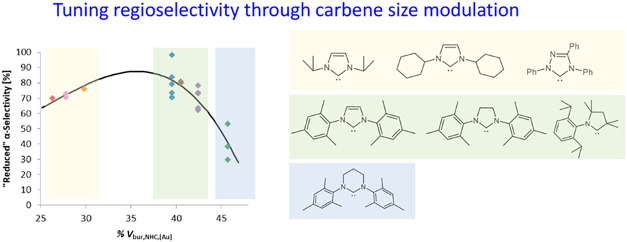

## Introduction

The synthesis of π-conjugated organic polymers through the cyclopolymerization of α,ω-diynes is of significant interest due to the promising optoelectronic properties of the resulting materials. Poly­(diyne)­s are photoconductive materials^1,2^ with applications in photovoltaics,^3^ and, when doped, they can be used as conductive materials.^4^ During the cyclopolymerization of α,ω-diynes, the monomer can undergo either α- or β-insertion (Figure 1). Regioselective cyclopolymerization facilitates the selective incorporation of α- or β-insertion derived units, directly influencing the properties of the polymeric material, including effective conjugation length (*N*_eff_), absorption maxima (λ_max_) and the HOMO–LUMO bandgap. For example, undoped poly­(diethyl dipropargylmalonate), (poly­(DEDPM)) containing >96% five-membered rings exhibit 600 times higher conductivity than that of a mixture of five- and six-membered rings.^5^ This underscores the importance of understanding the factors governing regioselectivity in these reactions.

**Figure 1:**
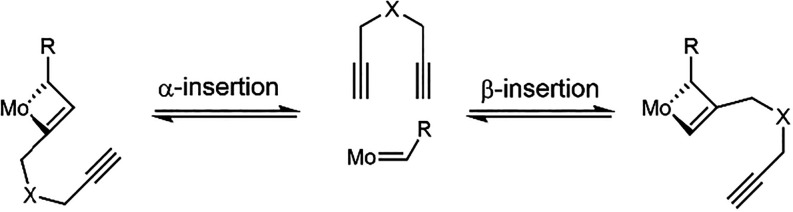
Monomer insertion in α- (left) and β-fashion (right).

The first living cyclopolymerization with well-defined molybdenum alkylidene catalyst was carried out by Schrock *et al.* and resulted in a polymer composed of a mixture of both α- and β-insertion derived dyads.^6,7^ Subsequently, selective β-addition was accomplished by applying the concept of small and large alkoxides.^8^ Our group reported on the synthesis of poly­(diyne)­s solely containing α-insertion-derived repeat units by employing fine-tuned Schrock type molybdenum alkylidene catalysts.^9,10^ The reports also outlined the positive effect of low temperature and addition of a coordinating base, such as quinuclidine, on α-selectivity. Ultimately, both neutral and cationic molybdenum imido alkylidene *N*-heterocyclic carbene (NHC) complexes demonstrated almost exclusive α-insertion across various 1,6-heptadiynes and 1,7-octadiynes.^11,12^ Despite the extensive body of data, a *comprehensive* and *quantitative* understanding of α-regioselectivity in the cyclopolymerization of 1,6-diynes with these type of molybdenum carbene complexes is still missing and a *general*, exact picture of the role of the individual ligands still has to be developed. In fact, the role of the structure of the metal catalysts containing a coordinating carbene such as an NHC or a cyclic alkyl amino carbene (CAAC), and particularly the role of the coordinated carbene, has not been yet systematically explored. In this study, we aimed to address this gap by evaluating a series of neutral and cationic molybdenum imido alkylidene catalysts bearing either CAAC or NHC ligands to establish a structure–activity relationship, together with the data obtained from DFT-optimized geometries.

## Results and Discussion

### Cyclopolymerization of DEDPM

In order to develop a precise structure–activity relationship between the steric demand of the carbene ligands in the ligand sphere and α-selectivity, a series of well-defined neutral and cationic molybdenum imido alkylidene complexes bearing either NHC or CAAC ligands have been investigated. These catalysts possess the general formulas Mo­(*N*-Ar)­(CHCMe_2_R)­(NHC)­(X)_2_ for the neutral species and [Mo­(*N*-Ar)­(CHCMe_2_R)­(NHC or CAAC)­(X)]^+^ [(BAr^F^)_4_]^−^ for the cationic analogues (Figure 2). The study focuses primarily on determining how the nature of the NHC or CAAC ligand exerts a direct influence on the α-selectivity observed in the cyclopolymerization of DEDPM (Scheme 1). To explore this, a variety of carbene ligands with different steric profiles were employed. These include small, less hindered NHCs such as IiPr, as well as bulkier carbenes like 6-Mes and CAAC-1.

**Figure 2:**
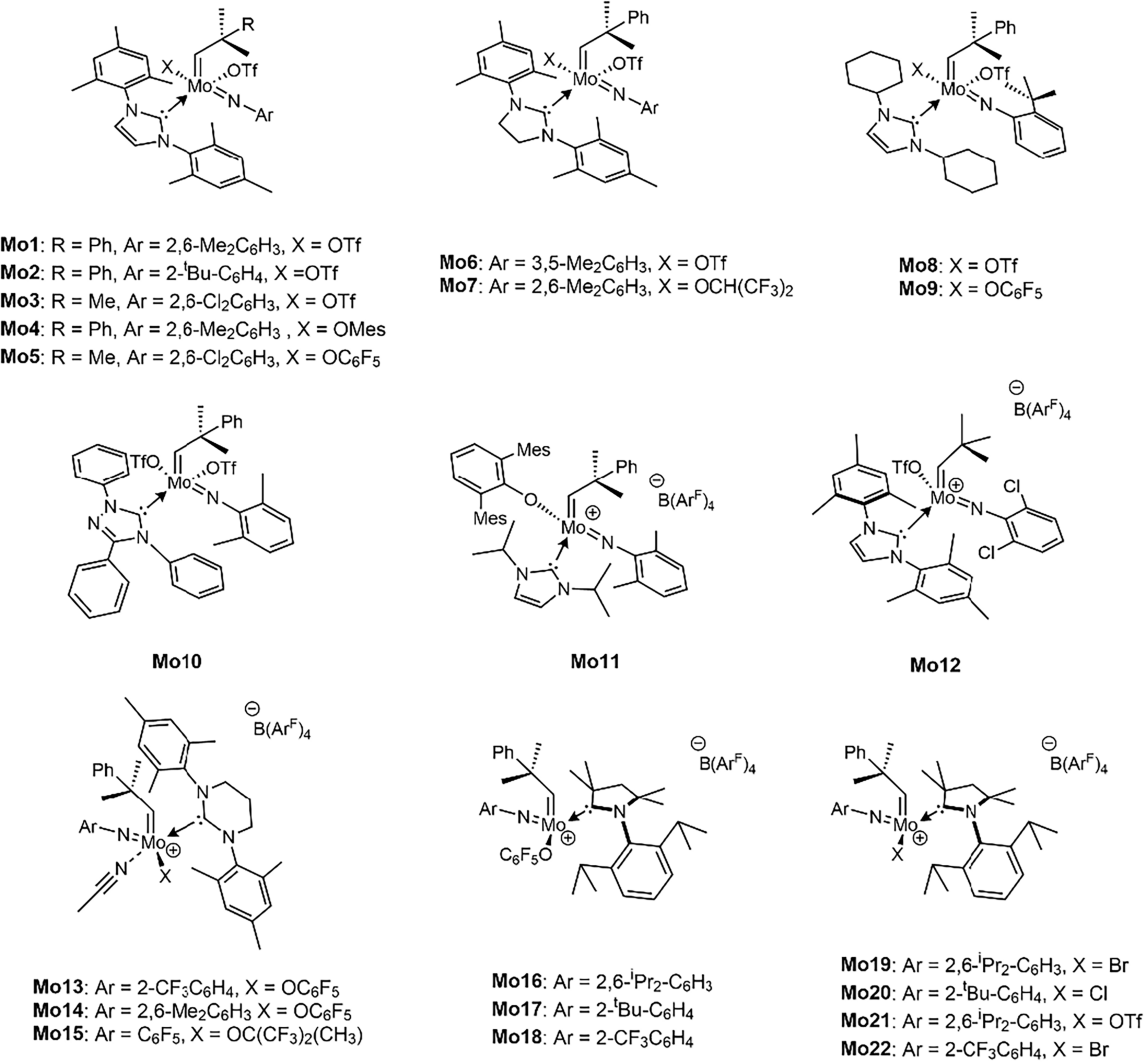
Initiators **Mo1–Mo22** used in the cyclopolymerization of DEDPM.

**Scheme 1:**
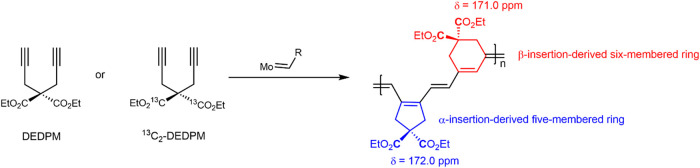
Structures of DEDPM, ^13^C_2_-DEDPM and α- and β-Insertion Derived Repeat Units

The ratio of α- or β-insertion derived units is conveniently determined by ^13^C NMR analysis.^6^ The α-insertion in 1,6-heptadiynes ultimately results in a five-membered ring formation, whereas β-insertion leads to the formation of a six-membered ring (Scheme 1). Therefore, the carbonyl carbons in the α- and β-insertion derived repeat units exhibit distinct chemical shifts in the ^13^C NMR, allowing for an unambiguous assignment and quantification of the insertion pattern.^6,11^ The chemical shift at δ ∼172.0 ppm corresponds to the α-insertion derived repeat units in poly­(DEDPM) while the chemical shift at δ ∼ 171.0 ppm corresponds to the β-insertion derived repeat units (Figure 3). Alternatively, the quaternary carbon (C_ipso_) also shows distinct signals for the two different insertions; a signal at δ = 57.5 ppm can be assigned to a five-membered ring while for a six-membered ring it appears at δ = 54 ppm. However, due to the poor solubility of the isolated polymers in CDCl_3_, obtaining spectra of sufficient quality can be challenging in some cases. To address this, ^13^C-labeled monomer (^13^C_2_-DEDPM) was employed for selected catalysts.

**Figure 3:**
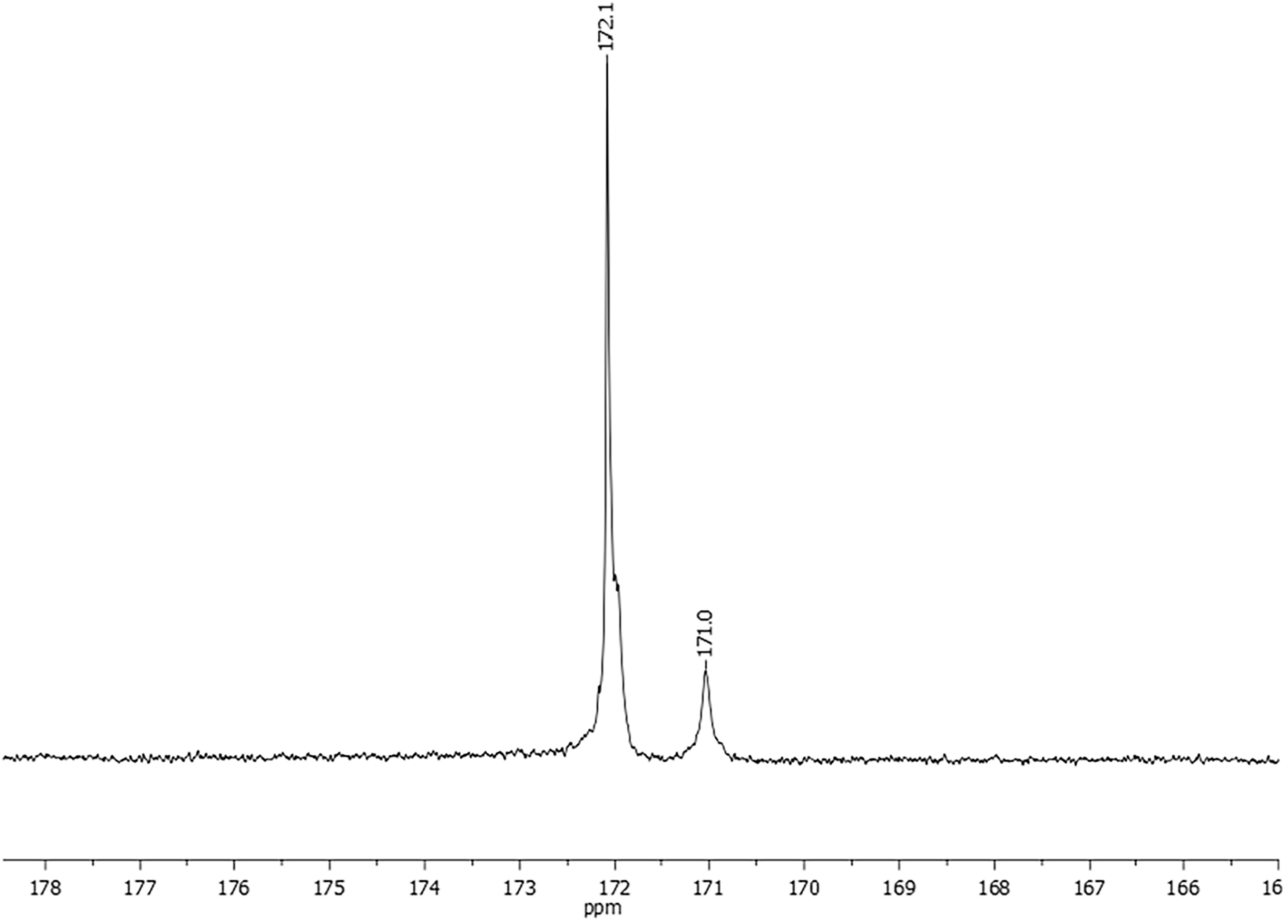
Carbonyl region of ^13^C NMR of poly­(^**13**^ **C**_**2**_ **-DEDPM**) synthesized by the action of **Mo17**.

A solution of the initiator in 1,2-dichloroethane was added in one shot to a solution containing 20 or 50 equiv of the monomer and the mixture was stirred vigorously to ensure homogeneity. The polymers were precipitated from pentane or methanol and dried thoroughly before subjecting to NMR analysis to determine the regioselectivity of insertion with these initiators. Data are summarized in Table 1. All initiators were highly reactive and immediately yielded bright purple polymers. However, for catalysts **Mo6** and **Mo8**, high temperature (up to 80 °C) was required to achieve reasonable conversion. Most initiators showed moderate to high α-insertion derived dyads in line with our previous observations with molybdenum alkylidene NHC initiators.^11,12^ In some polymers with a relatively low content of α-insertion-derived repeat units, a shoulder adjacent to the main signal, corresponding to the carbonyl group of the five-membered repeat unit (δ ∼ 172.0) was observed in the ^13^C NMR spectra. This feature arises from variations in the chemical environment of the individual repeat units at different positions along the polymer chain, where an α-insertion-derived repeat unit is neighbored by either two α- or one or two β-insertion-derived repeat unit. Selected polymer samples were subjected to size-exclusion chromatography (SEC) to determine the number-average molecular weights (*M̅n*) and polydispersities (*Đ*, Table 1). The high *Đ*-values indicate that the polymerizations do not proceed in a controlled manner. This observation is consistent with the very high reactivity of neutral and cationic molybdenum imido alkylidene NHC complexes reported previously.^11,12^ Representative UV–vis absorption spectra were also recorded for poly**(DEDPM)**·**Mo3** (>98% α), poly**(DEDPM)**·**Mo8** (43% α) and poly**(DEDPM)**·**Mo9** (68% α), as they differ in their proportions of α- and β-insertion-derived units. High α-insertion-derived poly­(DEDPM) showed absorption maxima (λ_max_) of 586 nm, while low α-insertion-derived poly­(DEDPM) had a λ_max_ of 541 nm.

**Table 1:** Cyclopolymerization of **DEDPM** with Catalysts **Mo1**–**Mo22** ^a^

catalyst	carbene	isolated yield (%)	α-selectivity (%)	*M̅n* (kg/mol)	*Đ*
**Mo1**	IMes	58	90		
**Mo2**	IMes	49	≥88	12	3.0
**Mo3**	IMes	>99	>98	10	2.3
**Mo4** ^b^	IMes	93	>99		
**Mo5** ^b^	IMes	>99	>99		
**Mo6** ^c^	IMesH_2_	92	81		
**Mo7** ^13^ ^e^	IMesH_2_	54	≥96	67	2.7
**Mo8** ^d^	ICy	99	43		
**Mo9** ^e^	ICy	99	68	34	1.7
**Mo10**	TPT	95	77		
**Mo11** ^b^	IiPr	>99	86	79	2.2
**Mo12** ^b^	IMes	>99	>97	9.3	1.6
**Mo13** ^b^	6-Mes	>99	37	15	1.6
**Mo14** ^b^	6-Mes	>99	44	12	1.6
**Mo15** ^b^	6-Mes	>99	72	45	2.5
**Mo16**	CAAC-1	94	60	37	1.7
**Mo17**	CAAC-1	>99	86	52	2.3
**Mo18**	CAAC-1	>99	88		
**Mo19**	CAAC-1	94	92	14	2.5
**Mo20**	CAAC-1	96	96	30	3.1
**Mo21**	CAAC-1	40	88	16	3.1
**Mo22**	CAAC-1	62	80		

a Polymerization conditions: 1,2-dichloroethane, room temperature, 2 h, catalyst: monomer = 1:50.

b 1,2-dichloroethane, room temperature, 2 h, catalyst: monomer = 1:20.

c CHCl_3_, 70 °C, 1 h, catalyst: monomer = 1:50;

d 1,2-dichloroethane, 80 °C, 20 h, catalyst: monomer = 1:50;

e CH_2_Cl_2_, −30 °C to room temperature, 1 h, catalyst: monomer = 1:50. number-average molecular weights (*M̅n*) and polydispersities (*Đ*) were determined by SEC in CHCl_3_ vs narrow polystyrene standards.

Selective α-insertion was observed with catalysts **Mo3**, **Mo4** and **Mo5**. Interestingly, some of the initiators showed higher preference for β-insertion, resulting in up to 63% β-insertion derived product. The catalysts that exhibited lower preference to α-insertion mostly contained a bulky NHC, such as 6-Mes, and/or a bulky anionic “X” ligand. **Mo13** and **Mo14**, both containing the bulky 6-Mes ligand, resulted in the lowest proportion of α-insertion derived product, i.e., 37 and 44% respectively. In line with this observation, **Mo16** bearing bulky CAAC-1 and pentafluorophenoxide ligands resulted in only 60% α-insertion. In contrast, **Mo11** containing the very bulky O-2,6-dimesitylphenoxide (OHMT) and the comparably small IiPr ligand still resulted in 86% α-insertion-derived repeat units. This suggests that the steric profile of the NHC or CAAC ligand is one major determinant in deciding insertion selectivity. The effect of the imido ligand was evaluated by comparing catalysts **Mo16**, **Mo17** and **Mo18**, which only differ in the imido ligand. In both **Mo16** (2,6-di*iso*propylphenyl imido) and **Mo17** (2-*tert*butylphenyl imido), the imido ligands are bulkier than in **Mo18** (2-trifluoromethylphenyl imido). Despite the similar steric demand of the imido ligands, **Mo16** and **Mo17** exhibit markedly different α-selectivity (60% and 86%, respectively), whereas **Mo18** shows a comparable α-selectivity (88%) to **Mo17**. Another pair of catalysts, **Mo19** (2,6-di*iso*propylphenyl imido) and **Mo22** (2-trifluoromethylphenyl imido), only differing in the imido ligands, showed α-selectivities of 92% and 80% respectively. However, **Mo19** bearing the bulkier 2,6-di*iso*propylphenyl imido ligand exhibited higher α-selectivity than the **Mo22** based on the 2-trifluoromethylphenyl imido ligand. Similarly, the effect of the “X” ligand can be evaluated by comparing **Mo1** (X = OTf) and **Mo4** (X = OMes) as they only differ in the “X” ligand. The α-selectivity increases from 90% (**Mo1**) to >99% (**Mo4**), suggesting that α-selectivity may increase upon introducing bulkier “X” ligands. However, an opposite trend is observed in another set of complexes, **Mo16** (X = OC_6_F_5_), **Mo19** (X = Br), and **Mo21** (X = OTf). In this case, **Mo16**, which bears the bulky OC_6_F_5_ ligand, exhibits the lowest α-selectivity (60%). A similar pattern is seen between **Mo17** (X = OC_6_F_5_, 86% α) and **Mo20** (X = Cl, 96% α), where the smaller chloride ligand leads to higher α-selectivity. Interestingly, this trend reverses again between **Mo18** (X = OC_6_F_5_, 88% α) and **Mo22** (X = Br, 80% α), where the bulkier OC_6_F_5_ ligand results in greater α-selectivity. These observations suggest that the influence of the imido ligand and the “X” ligand on regioselectivity cannot be interpreted independently, but rather must be considered in conjunction with the effects of other ligands in the coordination sphere.

To investigate the influence of monomer steric bulk, **Mo16**, a catalyst showing poor selectivity in polymerization of DEDPM, was chosen for additional experiments. Polymerization of a bulkier monomer 4-(ethoxycarbonyl)-4-(1S,2R,5S)-(−)-menthoxycarbonyl-1,6-heptadiyne (**M2**) was performed under identical conditions used for the polymerization of DEDPM with **Mo16**. Due to the asymmetry of the monomer, the two carbonyl carbons are inequivalent and therefore, the signals corresponding to the carbonyl carbons in ^13^C NMR spectra cannot be used to exactly determine the ratio of the α- and β-insertion-derived units. Therefore, the integral of the *ipso* carbon was used here to determine the α:β ratio. Only one peak corresponding to the *ipso* carbon showed up at δ = 57.5 ppm^11^ in the ^13^C NMR spectra of poly­(**M2**) obtained by the action of the **Mo16**, indicating >95% α-addition. The marked increase in α-content in poly­(**M2**) relative to poly­(DEDPM) suggests that α-content might be proportional to the size of the monomer’s ester substituent. Accordingly, the smaller monomer dimethyl dipropagylmalonate (**M3**) was polymerized using **Mo16**. Consistent with previous observations, the resulting poly­(**M3**) contained only 55% α-insertion–derived units, indicating virtually no selectivity.

### Mechanism

In the cross-metathesis with 2-methoxystyrene, it was previously observed that neutral pentacoordinated 16-electron molybdenum imido alkylidene bistriflate or monotriflate monoalkoxide NHC catalysts release one triflate upon substrate coordination, resulting in a cationic propagating species and the findings were also further supported by quantum chemical investigations.^14^ Accordingly, it is reasonable to assume that cyclopolymerization also involves a cationic propagating species, irrespective of whether the catalyst is introduced in a neutral or ionic form. All the neutral complexes tested in this work contain at least one triflate group. In view of their excellent leaving group propensity, it is reasonable to assume that one triflate group leaves upon coordination of the monomer, resulting in the formation of the cationic propagating species (Scheme 2).

**Scheme 2:**
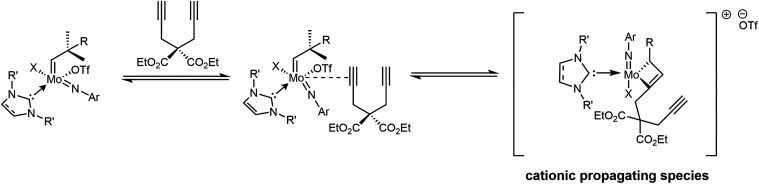
Formation of an α-Insertion-Derived Cationic Propagating Species from a 16-Electron Molybdenum Imido Alkylidene Bistriflate Or Monotriflate Monoalkoxide NHC Complex

The mechanism of cyclopolymerization catalyzed by molybdenum imido alkylidene complexes was first proposed by Schrock *et al.* and is widely accepted as the general pathway for all metal–alkylidene-catalyzed cyclopolymerizations.^6,7,15^ This mechanism can be directly adapted to both neutral and cationic molybdenum imido alkylidene complexes bearing NHC or CAAC ligands, as illustrated in Scheme 3. The mechanism proceeds via the formation of two molybdacyclobutenes, which are considered to be the deciding geometries for the regioselectivity of the employed catalyst. First, one of the two alkyne moieties of the diyne coordinates to the metal at a *trans* position to the strongest σ-donating CAAC or NHC ligand,^16–19^ either in α- or in β-fashion. α-Addition results in the molybdacyclobutene 4r-1α, where the carbon in α-position to the metal center is trisubstituted, while β-addition results in the molybdacyclobutene 4r-1β where the β-carbon is trisubstituted (Scheme 3). During the first step the molybdenum adapts a trigonal bipyramidal (TBP) geometry where the molybdacyclobutene and the carbene ligand form the equatorial plane. Once the molybdacyclobutene opens up through a productive cycloreversion, the alkylidene carbon can be either disubstituted (α-insertion) or monosubstituted (β-insertion). Next, an intramolecular interaction takes place between the second alkyne moiety of the monomer and the metal alkylidene, which results in the formation of the second molybdacyclobutene (4r-2α and 4r-2β in Scheme 3). Finally, the second molybdacyclobutene opens up to result in either a five-membered, α-insertion-derived or six-membered, β-insertion-derived repeat unit. Overall, the mechanism consists of two key intermediates for each mode of insertion, each containing a molybdacyclobutene. Recently, in our study on stereoselective ring-opening metathesis polymerization (ROMP) we demonstrated that in order to understand a ligand’s effect on the selectivity of the catalyst during a polymerization process, it is important to consider the presence of *all* ligands in the transition state, including the molybdacyclobutene and the truncated polymer chain on it, as they can directly or indirectly influence each other’s spatial orientation.^20^ This concept is particularly interesting in the context of the cyclopolymerization of DEDPM, as the monomer contains two ester groups. Depending on the flexibility of the polymer chain and steric demand of the ligand sphere, an ester group might interact with the metal center.

**Scheme 3:**
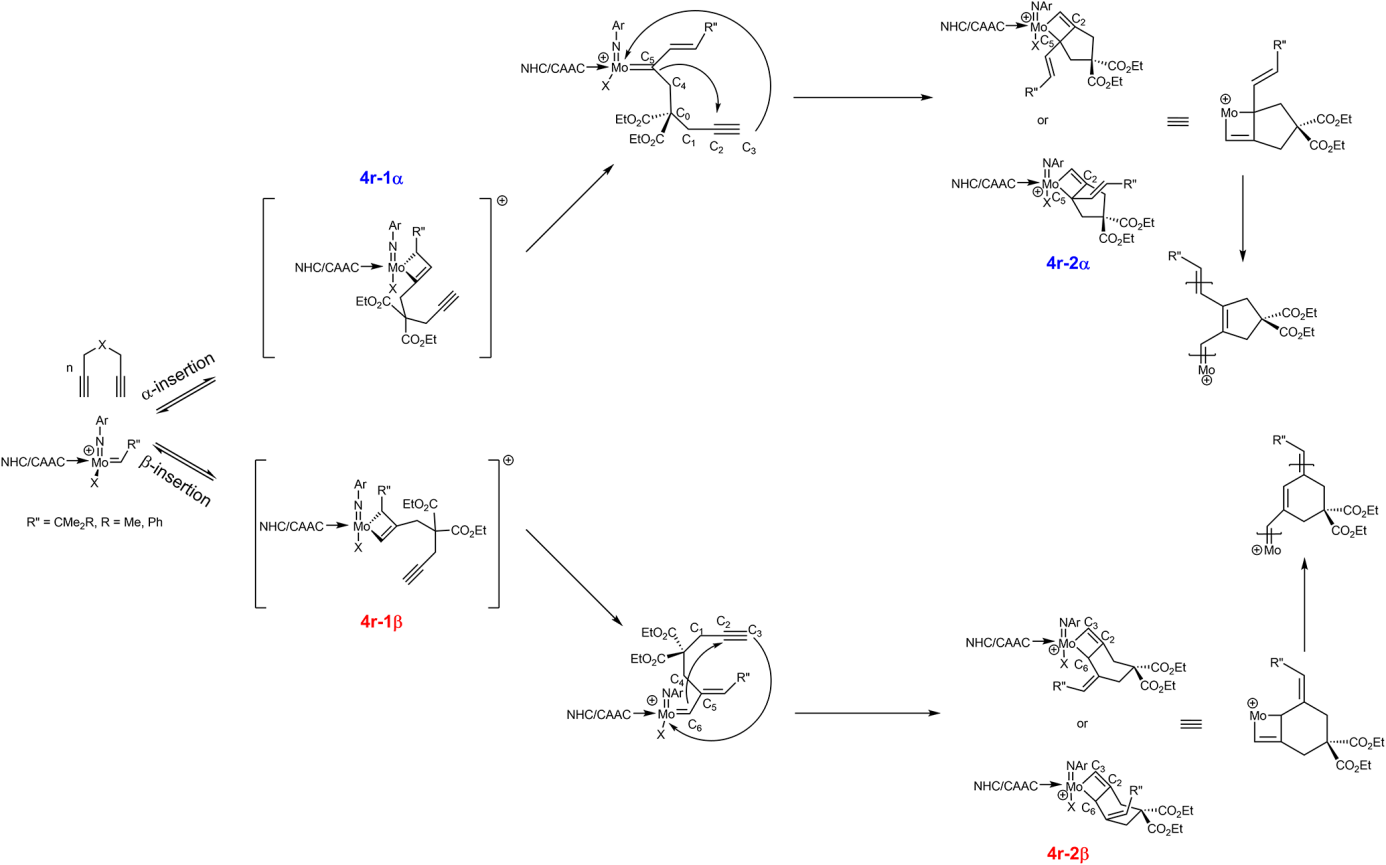
Mechanism for the Cyclopolymerization of DEDPM by Cationic Molybdenum Imido Alkylidene NHC and CAAC Complexes

### Computational Investigations

To investigate the origin of selectivity, theoretical studies were conducted with selected catalysts spanning a broad spectrum of selectivity. Given the time-consuming nature of locating transition states (TS), particularly in metathesis reactions, we focused instead on comparing minimum states (MS) across different catalysts.^21,22^ In order to incorporate the influence of the polymer chain, we included the first repeat unit of the chain into the calculation in place of R’’ in Scheme 3. Given the prevalence of the α-path for most catalysts, the corresponding repeat unit was chosen specifically. Consequently, the original alkylidene is not part of the computational model, since it only affects the start of the polymerization.

In previous studies it was shown that in the reaction of an α,ω-diyne with a cationic molybdenum imido alkylidene NHC catalyst the rate-determining step, i.e., the one with the highest barrier, is the formation of the first molybdacyclobutene.^21^ Similar was found for the reaction of α,ω-diynes with ruthenium-based catalysts,^21,23^ yet there the formation of the *second* ruthenacyclobutene is decisive for regioselectivity. In view of these findings, we carefully scrutinized the entire reaction coordinate of the reaction of DEDPM with catalyst **Mo3** (Figure 4). There, the first and fourth steps, which form π-complexes, are straightforward coordination events. The second step proceeds without a noticeable barrier in our calculations, an observation that likely applies to the fifth step as well, given their similarity. This narrows the potential rate-determining steps to the third and sixth steps. Both are very exergonic, indicating an early transition state. Consequently, MS 4r-1α, 4r-1β, 4r-2α, and 4r-2β were selected for further analysis. These are fairly similar to the relevant transition states, which conserve many of the relevant steric and electronic effects while being more easily accessible in calculation. The optimized structures provide a wide range of possible descriptors in order to explain or predict the experimentally observed selectivity. Among the most significant descriptors is the percentage buried volume (% *V*_bur_). % *V*_bur_ of a ligand is the fraction of a sphere, centered on the metal, occupied by that specific ligand.^24^ This value can be used to describe a ligand’s effective size and its steric hindrance on other ligands. After considering different parameters such as ligand–metal distances, ligand–metal–ligand angles, CM5 partial charges for the ligands and the metal center, van der Waals (vdW) volumes, and molar volumes,^25,26^ the most significant parameters were found to be the % *V*_bur_ of the carbene (% *V*_bur,NHC,4r‑2α_) in 4r-2α and the partial charge on the coordinating oxygen atom (*Q*_Ocoord,4r‑1α_) of the ester group of the monomer in intermediate 4r-1α (Figure 4).

**Figure 4:**
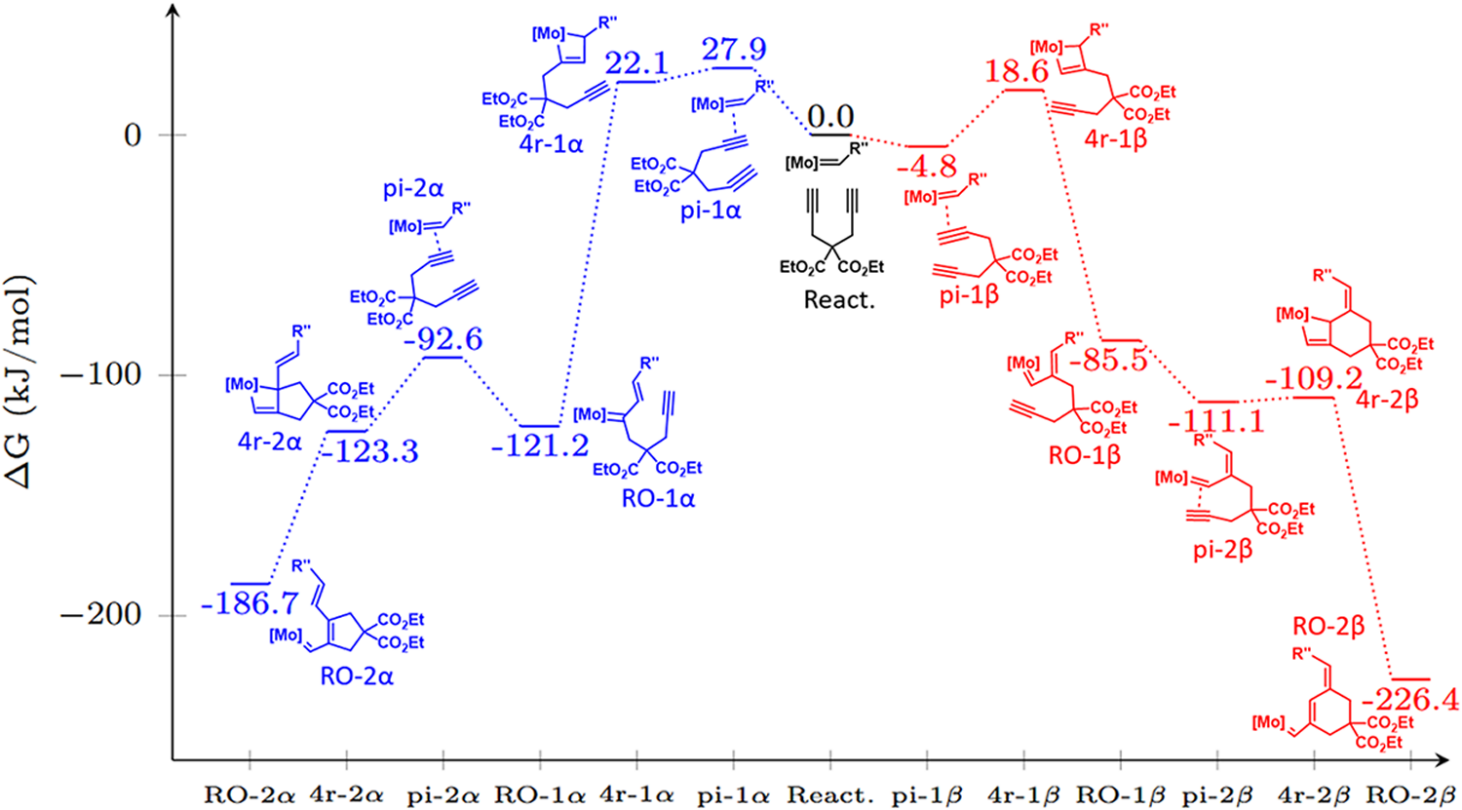
Calculated energies of all intermediates for both reaction paths for **Mo3**.

To quantitatively assess any effects on α-selectivity, the experimental α-selectivities were fitted by a combination of calculated % *V*_bur,NHC, 4r‑2α_ and *Q*_Ocoord,4r‑1α_. We have previously used a similar method to explain the selectivity in the ring-opening metathesis polymerization with similar types of catalysts.^20^ To construct a fit function here, Huber regression was used, which is a robust regression method that minimizes the impact of outliers via the Huber loss function.^27^ This can reduce errors introduced by the limited data set. The charges from the data set (Table S2, SI) were scaled to an interval [0;1] (Table S3, SI) and the coefficients are only given to the first decimal for readability here. The broadly best fit-function we found is

1 $$\alpha -\mathrm{selectivity}∼47.2\cdot {\%V}_{\mathrm{bur},\mathrm{NHC},4r-2\alpha }-82.1\cdot {\left(\%V_{\mathrm{bur},\mathrm{NHC},4r-2\alpha }\right)}^{3}+73.9\cdot Q_{\mathrm{Ocoord},4r-1\alpha }-105.5{\cdot \left(Q_{\mathrm{Ocoord},4r-1\alpha }\right)}^{2}$$

Since in the fit function (eq 1) the α-selectivity depends on both % *V*_bur,NHC,4r‑2α_ and *Q*_Ocoord,4r‑1α_, a three-dimensional plot would be necessary to fully describe the dependencies. Therefore, eq 1 can be reformulated to include any variance introduced by *Q*_Ocoord,4r‑1α_ in the α-selectivity

2 $$\mathrm{reduced \alpha}-\mathrm{selectivity}=\alpha -\mathrm{selectivity}-73.9\cdot Q_{\mathrm{Ocoord},4r-1\alpha }+105.5{\cdot \left(Q_{\mathrm{Ocoord},4r-1\alpha }\right)}^{2}∼47.2{\cdot \%V}_{\mathrm{bur},\mathrm{NHC},4r-2\alpha }-82.1{\cdot \left(\%V_{\mathrm{bur},\mathrm{NHC},4r-2\alpha }\right)}^{3}$$

This fit function (eq 2) matches the data with a good *R* ^2^ of 0.854 and an RMSE of 7.24% (Figure 5). It should be noted that % *V*_bur,NHC_ seems to be a critical variable. A cubic fit with % *V*_bur, NHC,4r‑2α_ alone was enough to explain 65% of the variance in the system. We found that 4r-1α can be stabilized by an additional coordination of the ester oxygen atoms.^12^ Such coordination is especially favored, since it is part of a six-membered ring (Figure 6, left) and results in O–Mo distances within a range of 2.31 to 3.31 Å. The molybdacyclobutene, the NHC/CAAC ligand, and the coordinating oxygen atom are all equatorially bound to the metal center. Consequently, and in line with the concept of large and small alkoxides,^28^ very *large* NHCs, such as 6-Mes, sterically hinder the coordination of oxygen, thereby lowering α-selectivity. Notably, coordination of oxygen is also not possible in minimum 4r-1β, since the ester group is located further away from the metal center.

**Figure 5:**
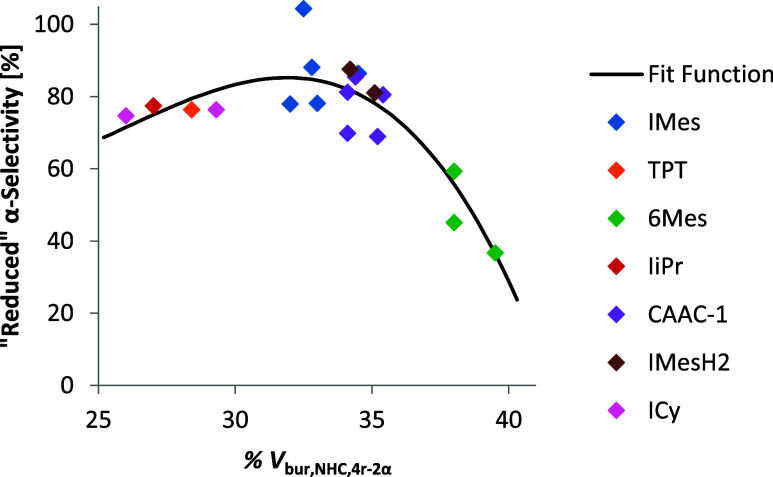
Plot of the “reduced” α-selectivity vs the % *V*_bur_ of the NHC calculated based on the computational structures of the 4r-2α intermediate.

**Figure 6:**
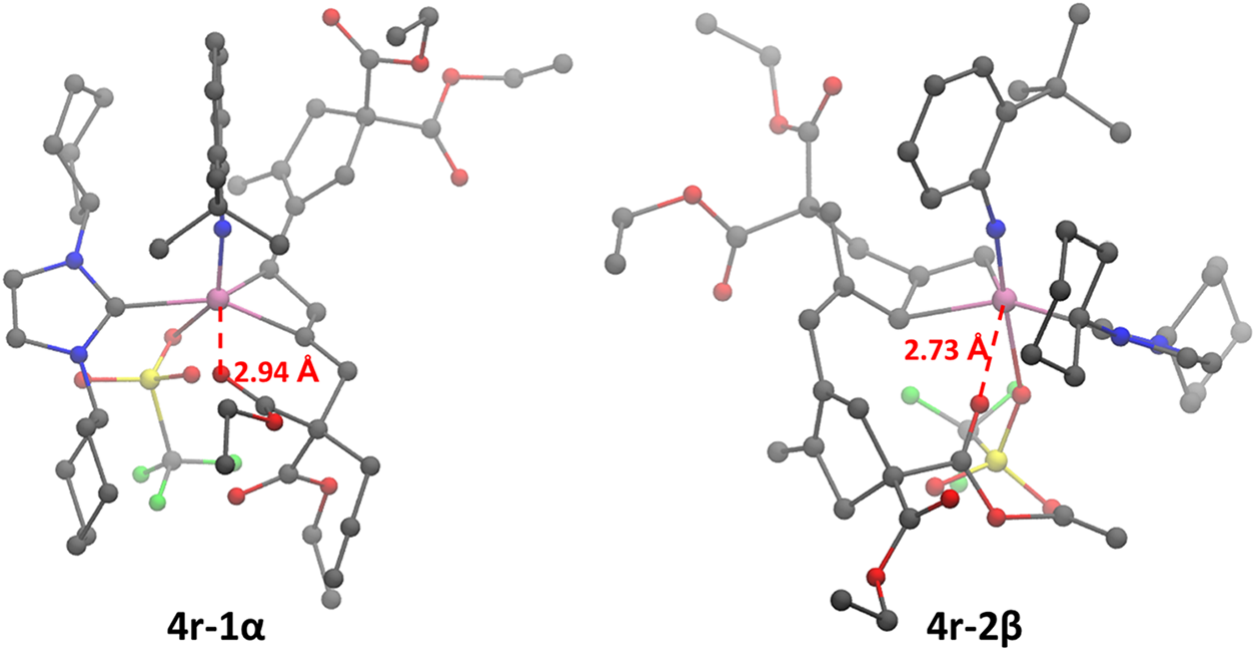
Steric hindrance and O–Mo coordination (red, dashed) drive the selectivity in two key intermediates in **Mo8**.

However, according to eq 2, very small NHCs also lower the α-selectivity. Coordination of the ester group may also facilitate β-addition since in 4r-2β, the ester group of the repeat unit adjacent to the molybdacyclobutene can coordinate to the metal (Figure 6, right). For 4r-2β this repeat unit is connected to the molybdacyclobutene via a short, inflexible connection (=CH−), allowing for the necessary closeness between the ester group and the metal center for such a coordination. In intermediate 4r-2α, the analogous connection is longer and more flexible (−CH=CH−), resulting in the ester groups being located more far away from the metal center. As a result, 4r-2β is the only of the two MS that can engage in this secondary coordination. Calculations indicate that this coordination occurs only for a few catalysts, primarily those with very small NHCs, such as ICy or TPT. Since the oxygen atom is again equatorially bound, it is sterically hindered by the NHCs. In this case, due to the larger side chain being close to the metal center, sufficient steric hindrance can be achieved even by medium-sized NHCs. The coordination in 4r-2β also appear to be much weaker than the ones appearing in 4r-1α, with the shortest O–Mo distance still being 2.73 Å. This is also reflected in the asymmetry of the graph shown in Figure 5.

The partial charge is more complicated to interpret. The charge of the coordinating oxygen in 4r-1α *Q*_Ocoord,4r‑1α_ is not correlated with the O–Mo distance (*R* ^2^ = −0.068). However, it is correlated, e.g., with the buried volume of the polymer chain in intermediate 4r-2α (*R* ^2^ = 0.433). Thus, *Q*_Ocoord,4r‑1α_ can be understood as the sum of various effects, both steric and electronic, on the polymer *and* oxygen coordination in particular. While it is not an intuitive variable, it appears accurate and grants more credence to the importance of the oxygen coordination.

The imido and X-ligands showed little correlation with α-selectivity. For instance, including both buried volumes as second-degree polynomials in the fit shown in eq 1 increased the *R* ^2^ only marginally, i.e., from 0.854 to 0.876 (SI). Similarly, incorporating the partial charges of all ligands raised *R* ^2^ only to 0.882 (SI). Because the inclusion of unnecessary variables typically reduces the robustness of a fit, these parameters were omitted. However, evidence was found for one possible effect originating from the X-ligand. **Mo11** shows no O–Mo coordination in 4r-2β and a decent α-selectivity of 86%, despite bearing a very small NHC (IiPr). The computational structures indicate that the “X” ligand and the polymer chain exist in close proximity, and therefore the “X” ligand can prevent the Mo–O coordination. The X-ligand is situated at the axial position in 4r-1α and 4r-2β as shown in Scheme 3 and Figure 6, and the coordinating oxygen atom in 4r-2β is typically closer to the “X” ligand than in 4r-1α. As a result, only the coordination in 4r-2β is hindered. **Mo11** has a far larger X-ligand than all other considered catalysts, so it is the only one where this effect is relevant. In eq 1, the effect of the X-ligand is contained in the variable *Q*_Ocoord,4r‑1α_.

Due to the absence of a larger data set with structurally diverse monomers, no variable is present in the fit to explicitly describe their influence. The experimental findings indicate that larger ester substituents on the monomer result in a propensity toward α-selectivity. The bulkier ester groups in the polymer chain can in principle also introduce greater steric hindrance between ligands; thereby suppressing additional coordination, such as the ones outlined above. In case the alkyl substituents of the ester group are sufficiently bulky, this can influence the ligands in a way that forces the oxygen into a position where it cannot further coordinate. However, in the computational structures, the alkyl substituents of the ester groups of the monomer are oriented away from the metal center and other ligands. This indicates that the size of the ester groups affects this coordination only minimally. The alteration in selectivity can instead be attributed to a steric conflict arising in the β-pathway between the ester substituents of the monomer and the polymer chain. As shown in Figure 4, the chains are positioned in proximity in the intermediates pi-1β and 4r-1β. Should an increase in bulk occur, more steric hindrance would be present, and the intermediates may increase the energy of the path.

### Statistical Evaluation

To estimate the accuracy of a model, the data is sometimes split into a training and a test set. However, given the limited amount of data, here we instead applied a 5-fold cross-validation, repeated 100 times.^29^ Each iteration used a different subset as the test set, with the remainder for training. The resulting averages of the coefficients are given in the following equation, with the errors in parentheses

3 $$\alpha -\mathrm{selectivity}∼49.5\left(12.8\right)\cdot {\%V}_{\mathrm{bur},\mathrm{NHC},4r-2\alpha }-84.3\left(11.9\right)\cdot {\left({\%V}_{\mathrm{bur},\mathrm{NHC},4r-2\alpha }\right)}^{3}+74.7\left(16.0\right)\cdot Q_{\mathrm{Ocoord},4r-1\alpha }-106.9\left(19.6\right)\cdot {\left(Q_{\mathrm{Ocoord},4r-1\alpha }\right)}^{2}$$

Another possible method to estimate the reliability of a model is *y*-randomization, in which the data set is systematically randomized in multiple ways.^30^ The α-selectivity can either be shuffled, replaced with random values between 35 and 100% or left as is. Similarly, the variables extracted from the computational structures can be retained or substituted by equal randomly generated variables (135, matching the number examined for the catalysts). For each of the scenarios, the optimal quadratic two-variable fit is identified. In a subsequent step, it was attempted to increase the *R* ^2^ of each fit by using the same variables but substituting alternative functional forms, such as exponential or cubic terms. The resulting *R* ^2^ and RMSE for all variable sets are summarized in Table 2.

**Table 2:** RMSE and *R* ^2^ for the Fit Function in eq 1 and Fit Functions Derived Using *y*-Randomizations, with the Same General Approach as the Original Fit Function

α-selectivity	fitting variables	*R* ^2^ (best fit)	RMSE (best fit)
from experiment	from calculations	0.854	7.239
from experiment	randomly generated	0.791	8.658
randomly shuffled	from calculations	0.703	10.314
randomly generated	from calculations	0.690	11.018
randomly generated	randomly generated	0.839	7.937
randomly shuffled	randomly generated	0.700	10.377

Although the fit obtained from the experimental and computational data represents the best-performing model, several fits generated through *y*-randomization approach similar quality and are, overall, not substantially worse. From a purely mathematical standpoint, this might question the reliability of our model. However, the *y*-randomization likely underestimates the true accuracy of our model here. First, many of the variables in the computational data show obvious correlation. For instance, the buried volume of the X-ligand is roughly the same no matter for which intermediate it is measured. Meanwhile, a randomly generated data set will have far fewer such correlations. Indeed, the fits based on a randomly generated data set possess, on average higher *R* ^2^ than the *y*-randomization fits based on the computational data. Second, had the fit function with the highest *R* ^2^ included variables lacking intuitive explanatory value, we would have either selected a different fit or reevaluated the mechanistic hypothesis. Consequently, the effective number of independent variables is much smaller, and any *y*-randomizations would be expected to yield substantially poorer fits. Because these effects are difficult to quantify, we nevertheless conducted the randomizations using all 135 variables.

### Disentangling of Steric Effects

The buried volume depends on the steric contributions of neighboring ligands, which compete for the same coordination sphere. As a result, a decrease in buried volume may reflect either a physically smaller ligand or reduced steric demand due to larger neighboring ligands. For instance, the additional oxygen coordination in 4r-2β and 4r-1α reduces the buried volume of the NHC/CAAC and makes these variables a much worse predictor for α-selectivity. Our model predicts that only the physical size of the ligands is relevant. To test this, the buried volumes of all NHC ligands were recalculated using less-crowded linear complexes, namely [(NHC)­AuCl].^31^ Here, steric interactions with other ligands are essentially absent. The resulting fit function retained the same general form, supporting the hypothesis

4 $$\alpha -\mathrm{selectivity}∼62.1\cdot {\%V}_{\mathrm{bur},\mathrm{NHC},\left[\mathrm{Au}\right]}-88.6{\cdot \left(\%V_{\mathrm{bur},\mathrm{NHC},\left[\mathrm{Au}\right]}\right)}^{3}+102.4\cdot {\mathrm{CM}5}_{\mathrm{Ocoord},4r-1\alpha }-132.3\cdot {\left({\mathrm{CM}5}_{\mathrm{Ocoord},4r-1\alpha }\right)}^{2}$$

Similar to eqs 1 and 2, eq 4 can also be reformulated to

5 $$\mathrm{reduced \alpha}-\mathrm{selectivity}=\alpha -\mathrm{selectivity}-102.4\cdot {\mathrm{CM}5}_{\mathrm{Ocoord},4r-1\alpha }+132.3\cdot {\left({\mathrm{CM}5}_{\mathrm{Ocoord},4r-1\alpha }\right)}^{2}∼62.1\cdot \%V_{\mathrm{bur},\mathrm{NHC},\left[\mathrm{Au}\right]}-88.6\cdot {\left({\%V}_{\mathrm{bur},\mathrm{NHC},\left[\mathrm{Au}\right]}\right)}^{3}$$

The resulting fit (eq 5) function again had a good *R* ^2^ of 0.855 and RMSE of 7.21%. This fit function suggests the opportunity for some further optimization of the catalyst, since none of the NHCs fall on the maximum of the curve (Figure 7). A slightly smaller NHC than IMes should theoretically be better. However, since the data set is limited and the influence of *Q*_Ocoord,4r‑1α_ is expected to impact the outcome, such a prediction must still be taken with some caution.

**Figure 7:**
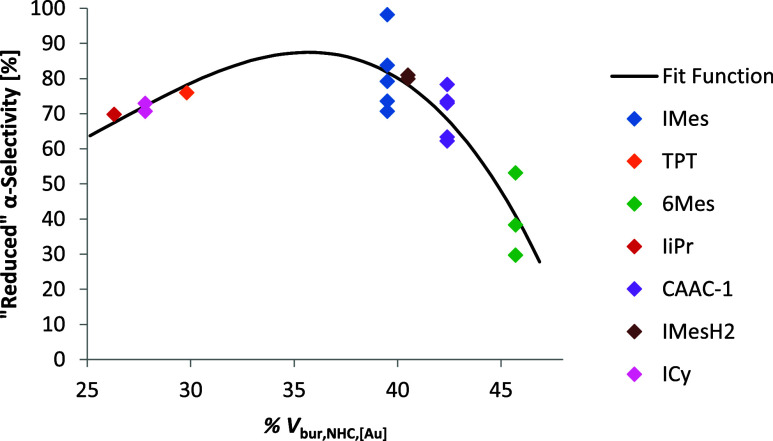
Plot of the “reduced” α-selectivity versus the % *V*_bur_ of the NHC/CAAC ligands; calculations are based on the computational structures of [(NHC)­AuCl].

## Conclusions

The regioselective cyclopolymerization of DEDPM has been investigated with a series of molybdenum imido alkylidene NHC and CAAC complexes. While the corresponding NHC complexes are literature-known to be able to regioselectively cyclopolymerize α,ω-diynes, this study demonstrates for the first time that CAAC analogues can also catalyze the cyclopolymerization of DEDPM with high α-selectivity. The complexes studied offered a wide range of α-selectivity, with values as low as 37% and as high as >99%, thereby reflecting the tunability of these systems. It was demonstrated that the steric demand of the NHC or CAAC directly influences regioselectivity and a correlation fit was obtained with an RMSE of 7.24%. Notably, both excessively bulky and small NHCs led to a decrease in α-selectivity. Most importantly, it was found that both molybdacyclobutenes 4r-1α and 4r-2α directly contribute to the catalyst’s α-selectivity, through the steric influence of the NHC ligand and a stabilizing chelation between the molybdenum center and the oxygen atom of the monomer, respectively. It was also found that a very bulky “X” ligand, such as OHMT, in combination with a rather smaller NHC (IiPr) can hinder any similar coordination in molybdacyclobutene 4r-2β, and thereby causing β-addition to be even less favorable. These findings collectively enhance the applicability of molybdenum imido alkylidene NHC/CAAC catalysts in the regioselective cyclopolymerization and can be used in catalyst design approaches for regulating regioselectivity in cyclopolymerizations.

## Experimental Section

Unless stated otherwise, all reactions involving metal complexes were performed under N_2_ atmosphere using either standard Schlenk techniques or in a glovebox (LabMaster 130, MBraun, Garching, Germany). Glassware was stored at 120 °C overnight and cooled in an evacuated antechamber. CH_2_Cl_2_, diethyl ether, toluene, pentane, and tetrahydrofuran (THF) were dried by a solvent purification system (SPS, MBraun). 1,2-Dichloroethane was dried over CaH_2_ and stored over 4 Å molecular sieves. Starting materials and reagents were purchased from Merck (Darmstadt, Germany), Alfa Aesar (Karlsruhe, Germany), or ABCR (Karlsruhe, Germany) and were used as received unless stated otherwise. ^1^H NMR, ^19^F NMR and ^13^C NMR (proton decoupled) measurements were carried out on a Bruker Avance III 400 at 400 MHz, 376 and 101 MHz, respectively. The ^13^C NMR spectra of selected polymers were recorded at the Institute of Organic Chemistry, University of Stuttgart, on a Bruker Avance III HD at 176 MHz using broadband decoupling. Chemical shifts are reported in ppm relative to the solvent signal. Data are reported as follows: chemical shift, multiplicity (s = singlet, d = doublet, t = triplet, q = quartet, quint = quintet, sept = septet, br = broad, m = multiplet), coupling constants (Hz) and integral. Single-crystal X-ray analysis was performed on a Bruker Kappa APEXII Duo diffractometer with Mo–K_α_-irradiation at the Institute of Organic Chemistry, University of Stuttgart, Germany. Initiators **Mo1**,^32^ **Mo2**,^14^ **Mo3**,^32^ **Mo5**,^32^ **Mo6**,^33^ **Mo7**,^13^ **Mo8** - **Mo10**,^32^ **Mo11**,^34^ **Mo12**,^35^ **Mo13** - **Mo15**,^36^ **Mo16** - **Mo22**,^37^ and monomers DEDPM^5^ and ^13^C_2_-DEDPM^12^ were synthesized according to the literature.

Deposition Numbers CCDC 2492418 (**Mo4**) contain the supplementary crystallographic data for this paper. These data are provided free of charge by the joint Cambridge Crystallographic Data Centre and Fachinformationszentrum Karlsruhe Access Structures service.

### Mo­(2,6-Me_2_C_6_H_3_)­(CHCMe_2_Ph)­(OTf)­(OMes)­(IMes) (Mo4)

To a solution of Mo­(N-2,6-Me_2_C_6_H_3_)­(CHCMe_2_Ph)­(OTf)_2_(IMes) (200 mg, 0.21 mmol, 1 equiv) in CH_2_Cl_2_ (3 mL) a solution of LiOMes (45 mg, 16 mmol, 1.5 equiv) in CH_2_Cl_2_ (3 mL) was added and the reaction mixture was stirred for 24 h at room temperature. Subsequent filtration over Celite and evaporation of the solvent led to an orange crude product, which was crystallized from CH_2_Cl_2_, diethyl ether and pentane at −35 °C to obtain the complex as an orange solid. **Yield:** 111 mg (56%). ^**1**^ **H NMR** (CDCl_3_): δ 14.35 (s, 1H), 7.50–7.46 (m, 5H), 7.26 (s, 2H), 6.99 (s, 3H), 6.86 (s, 2H), 6.79 (s, 2H), 6.48 (s, 2H), 2.68 (s, 6H), 2.41 (s, 3H), 2.40 (s, 3H), 2.23 (s, 6H), 2.20 (s, 6H), 2.09–2.08 (m, 9H), 2.00 (s, 6H); ^**19**^ **F NMR** (CDCl_3_): δ −78.47; ^**13**^ **C NMR** (CDCl_3_): δ 317.3 (CHCMe_2_Ph), 187.1 (C_carbene_), 160.2, 154.4, 151.0, 139.6, 135.6, 135.5, 133.4, 129.3, 129.0, 128.5, 128.2, 128.1, 127.4, 127.0, 126.1, 125.9, 124.8, 119.0 (q, ^1^ *J*_CF_ = 323.3, CF_3_SO_3_), 55.6, 37.1, 34.3, 29.5, 22.5, 22.3, 21.1, 20.8, 18.8, 18.4, 14.2. Elemental analysis (%) Calcd. for C_49_H_56_F_3_MoN_3_O_4_S: C 62.88 H 6.03 N 4.49; found: C 62.62 H 6.08 N 4.51.

### General Procedure for Cyclopolymerization

The monomer (30 mg, 0.12 mmol, 20 or 50 equiv) was dissolved in ∼2 mL 1,2-dichloroethane, then a solution of the catalyst (1 equiv) in ∼0.5 mL 1,2-dichloroethane was added in one shot. The reaction mixture was stirred at the indicated temperature for 2 h. The reaction mixture was then brought out of the glovebox, precipitated under air by dropwise addition into pentane or methanol (30 mL), centrifuged, decanted, and dried *in vacuo* overnight. The isolated polymers were stored under nitrogen. The proportion of α- and β-insertion derived repeat units was determined by ^13^C NMR in CDCl_3_.

### Computational Details

All calculations were performed with Turbomole Version 7.4.1 ^38^ via ChemShell^39^ and DL-FIND.^40^ Geometries were preoptimized with GFN2-xTB,^41^ with conformational sampling via the Conformer and Rotamer Ensemble Sampling Tool (CREST),^42^ and the polymer chain was truncated after the first repeating unit. Subsequently, the most stable conformer was optimized with B3LYP-D3­(BJ)/def2-SVP.^43–48^ For all molybdenum complexes, solvation effects (ε = 10.36 for 1,2-dichloroethane) were included using the COSMO^49^ model, whereas for gold, they were omitted.^50^ Single points were calculated at the B3LYP-D3­(BJ)/def2-TZVP level.^43–48^ Thermodynamic corrections at 298 K were calculated on the same level of theory as the geometry optimizations. All geometric, steric, and electronic parameters were extracted from the DFT-optimized structures. Buried volumes were calculated with SambVca 2.1^24^ and included hydrogen atoms. A sphere radius of 4.4 Å was used for molybd complexes and 4.7 Å for gold complexes, and standard settings otherwise. CM5^25^ partial charges were calculated at the same level of theory as the geometry optimizations, and the vdW and molar volumes were computed using MolVol v1.2.0.^26^

## Supplementary Material



## Notes

Additional experimental details (^1^H NMR, ^13^C NMR and ^19^F NMR spectra of the novel catalysts and ^13^C NMR spectra of all polymers) and calculated parameters (PDF)
