# Supplementary material for: Tuning Regioselectivity in Cyclopolymerization through Carbene Ligand Size Modulation in Molybdenum Imido Alkylidene Catalysts
Source: Macromolecules. 2026 Jan 22;59(3):1262–72. doi: 10.1021/acs.macromol.5c03366 (PMC12895512; doi:10.1021/acs.macromol.5c03366)
Supplement: Supplementary file 1 [file ma5c03366_si_001.pdf]

Supporting Information

Tuning Regioselectivity in Cyclopolymerization through Carbene Ligand Size Modulation in Molybdenum Imido Alkylidene Catalysts

Koushani Kundu,[1#] Severin Haid,[2#] Patrick Probst,[1] Laura Stöhr,[1] Philipp Hauser,[1] Wolfgang Frey,[3] Johannes Kästner,[2*] Michael R. Buchmeiser[1,4*]

[1]Institute of Polymer Chemistry, University of Stuttgart, Pfaffenwaldring 55, 70569 Stuttgart, Germany; [2]Institute of Theoretical Chemistry, University of Stuttgart, Pfaffenwaldring 55, 70569 Stuttgart, [3]Institute of Organic Chemistry, University of Stuttgart, Pfaffenwaldring 55, 70569 Stuttgart, [4]German Institutes of Textile and Fiber Research (DITF), Körschtalstr. 26, 73770 Denkendorf, Germany

Table of Contents

1. NMR Spectra 1
2. UV-Vis Spectra 16
3. Crystal Data and Structure Refinement for Mo4 18
4. Calculations 20

1. NMR Spectra

Note: The ^{13}C NMR spectra of poly(DEDPM) are only depicted in the region of the decisive carbonyl group.

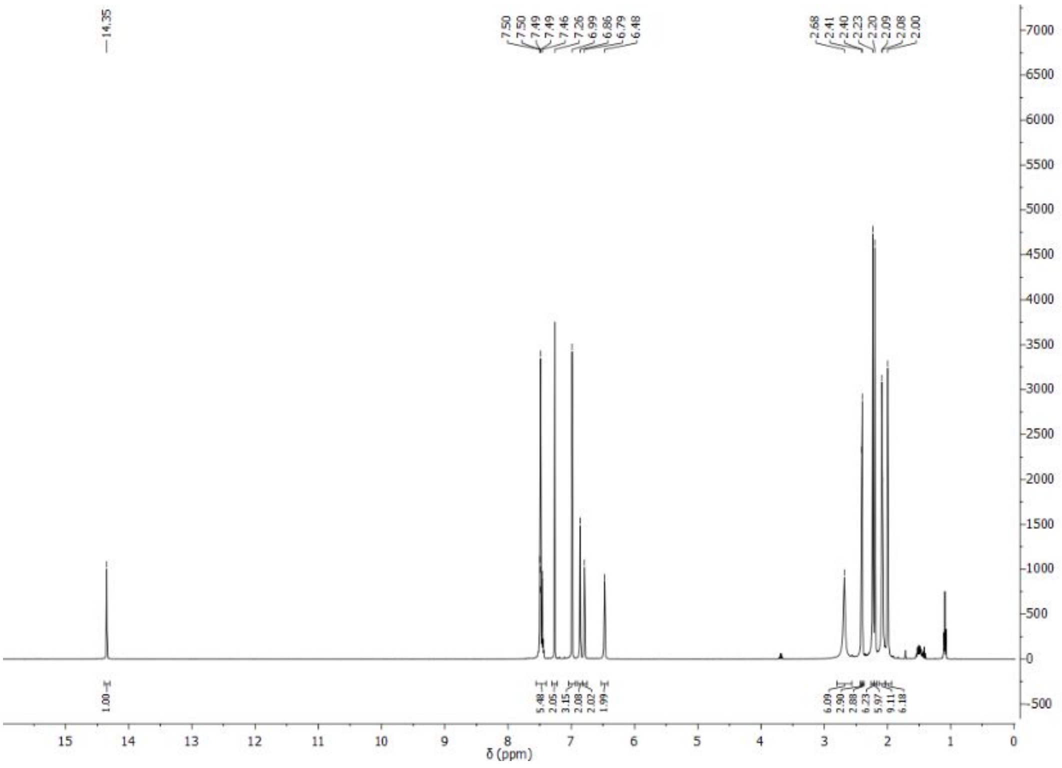

Figure S1. ^{1}H NMR spectrum of **Mo4** in $CDCl_3$.

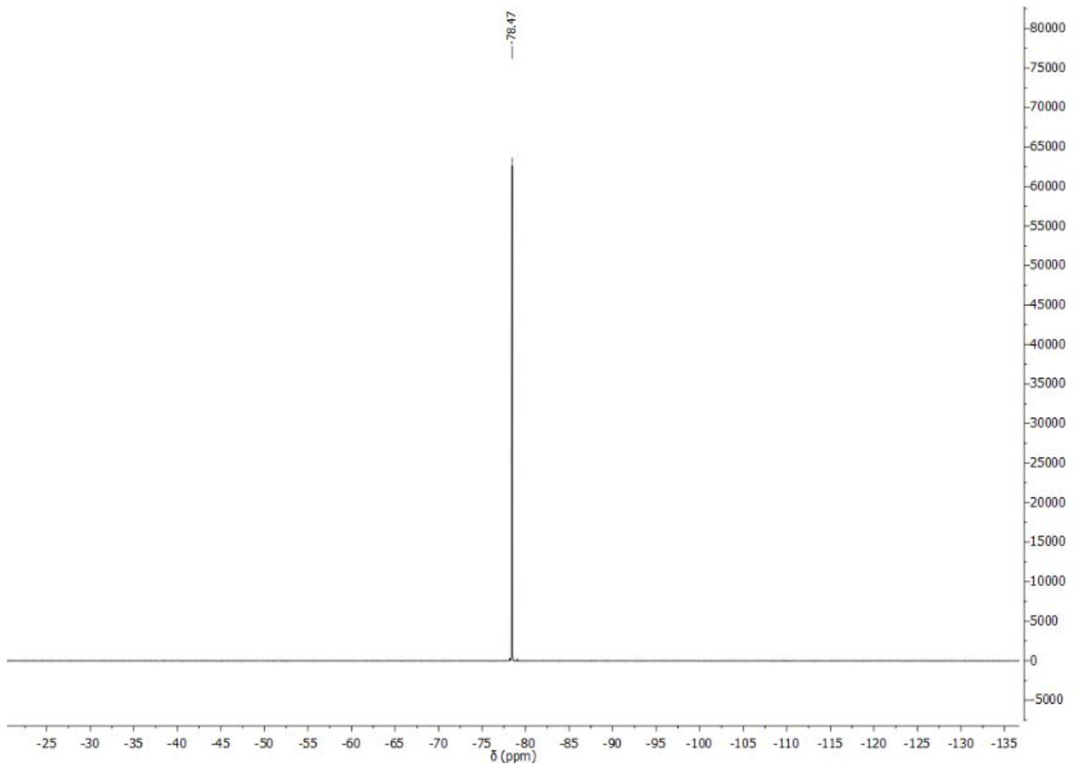

Figure S2. ^{19}F NMR spectrum of **Mo4** in $CDCl_3$.

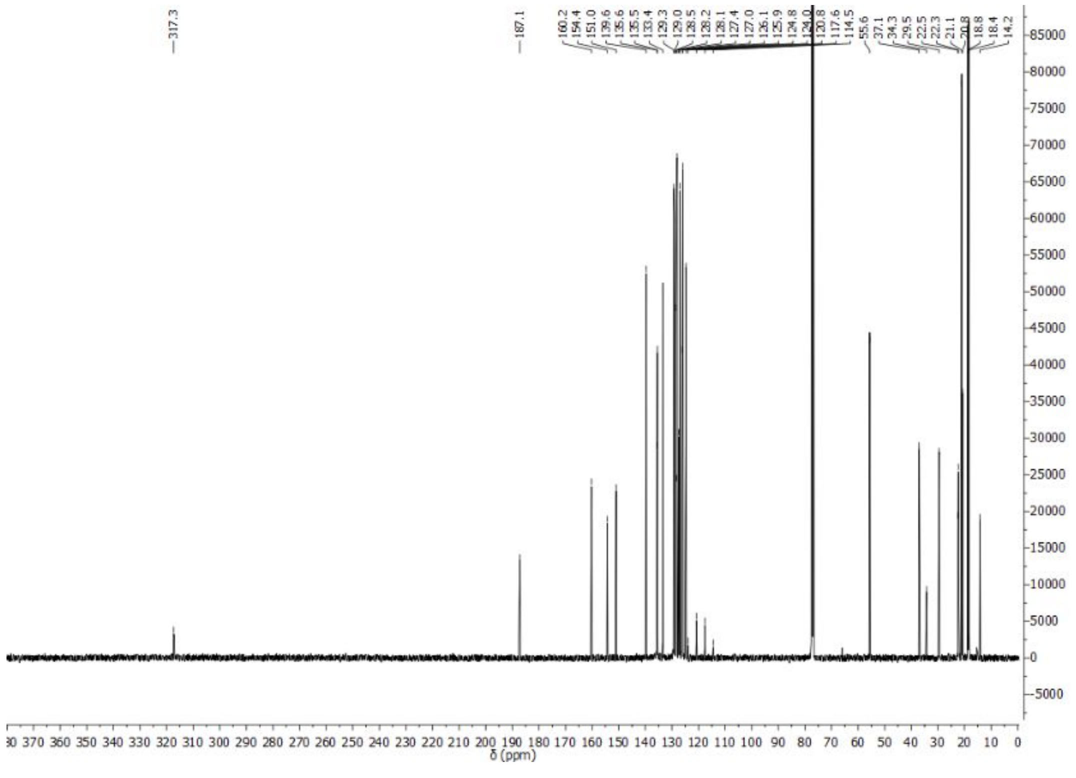

Figure S3. ^{13}C NMR spectrum of **Mo4** in $CDCl_3$.

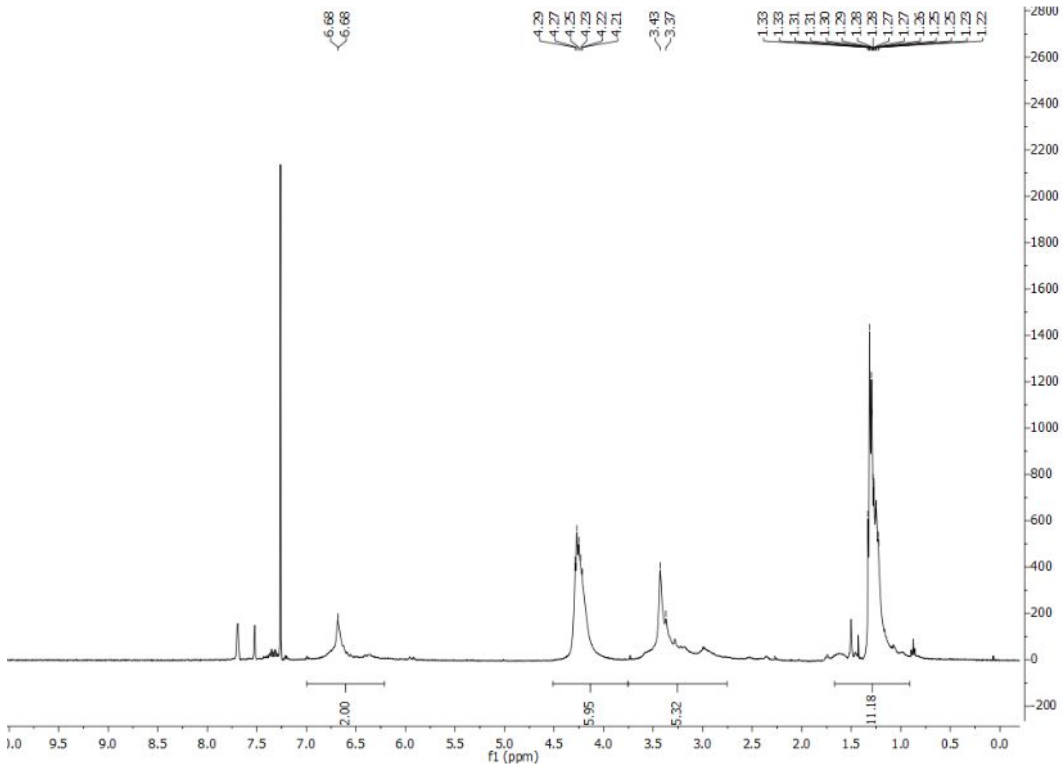

Figure S4. Representative ^{1}H NMR spectrum of poly(**DEDPM**) in $CDCl_3$.

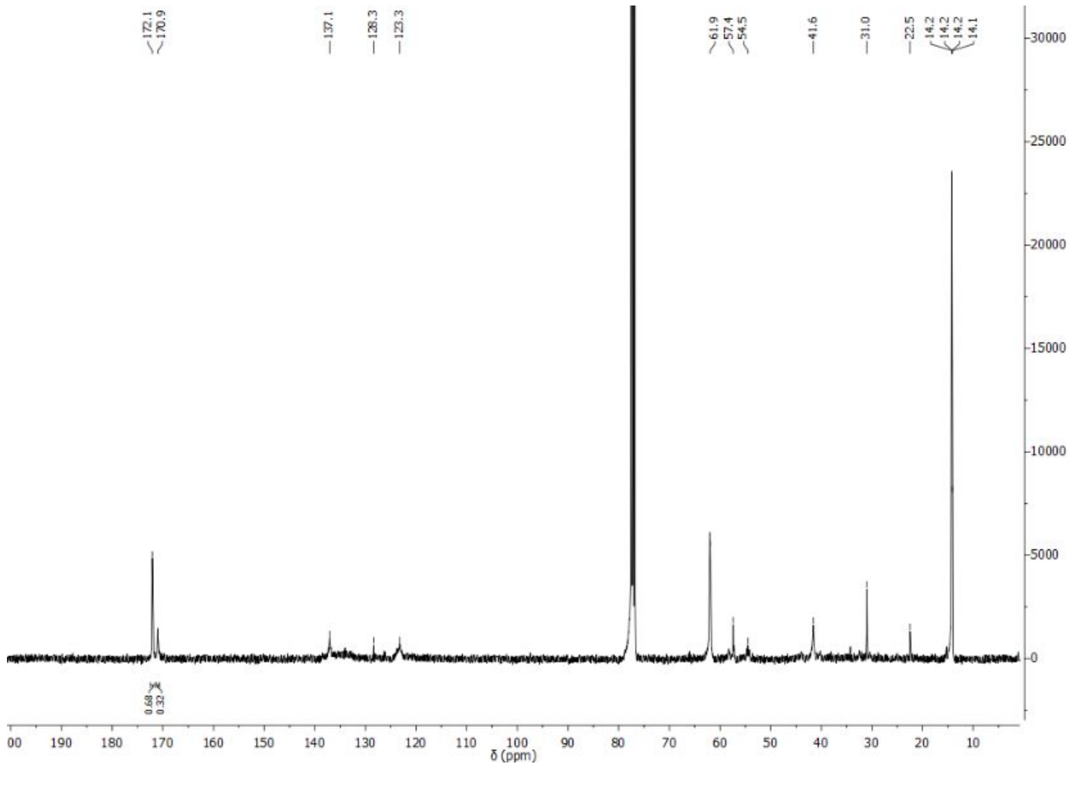

Figure S5. Representative ^{13}C NMR spectrum of poly(**DEDPM**) in $CDCl_3$.

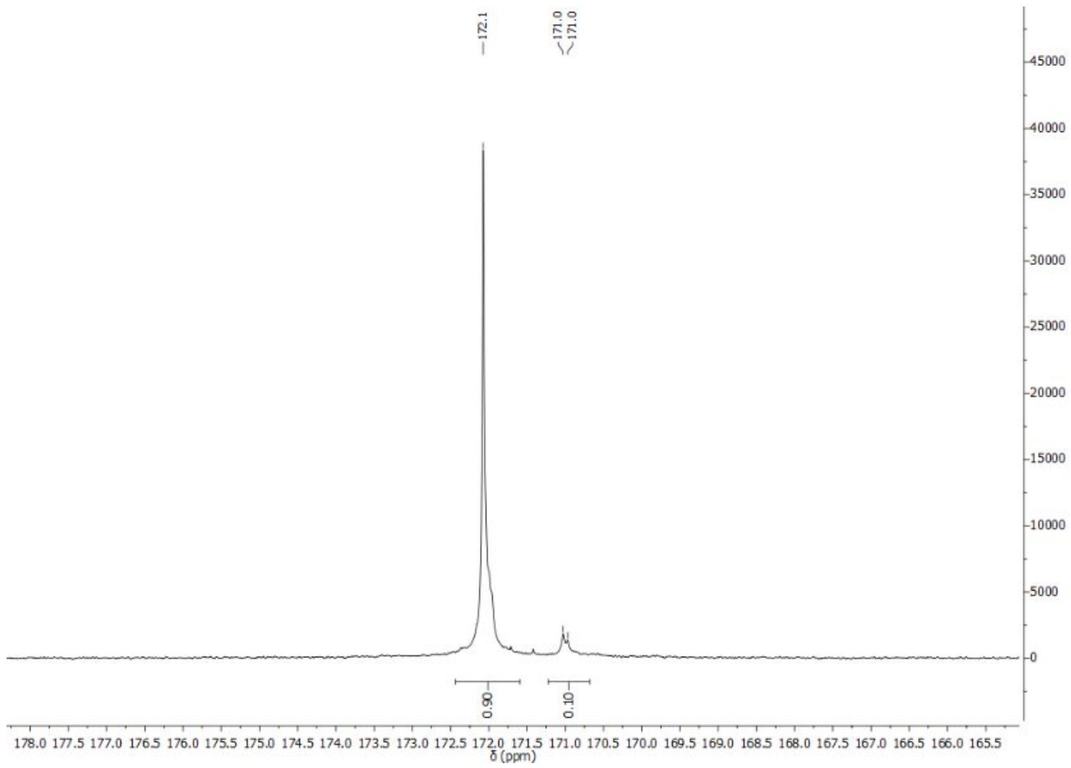

Figure S6. ^{13}C NMR spectrum ($CDCl_3$) of poly(**$^{13}C_2$-DEDPM**) obtained by the action of **Mo1**.

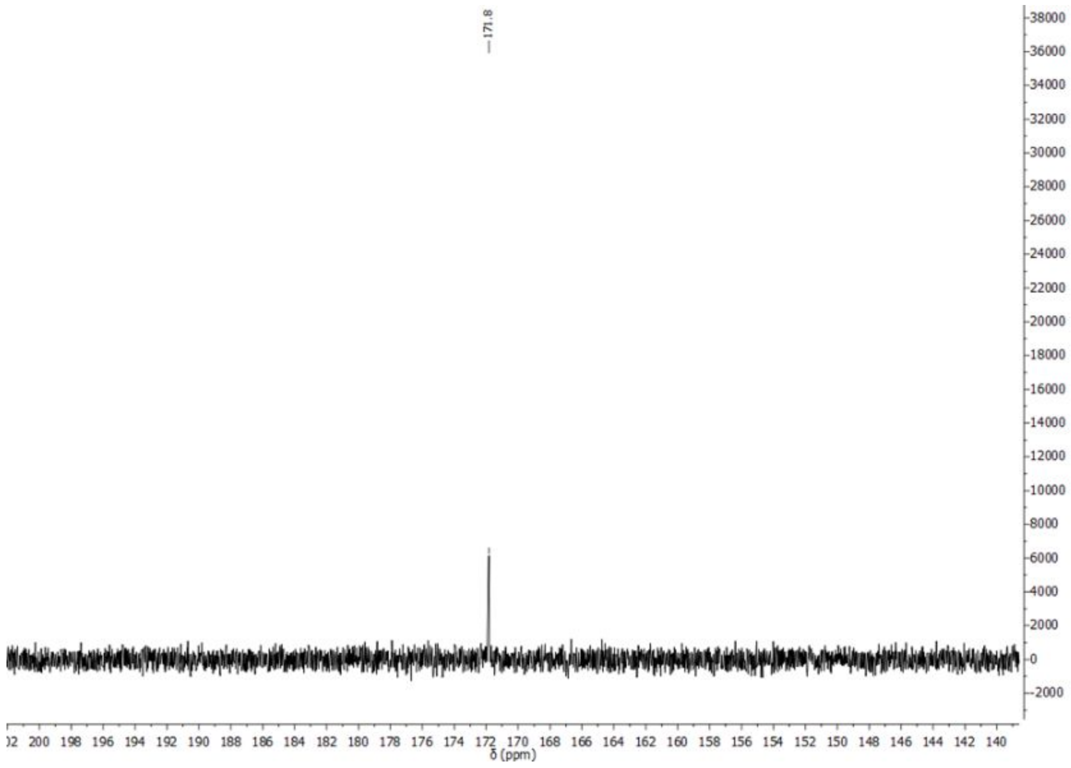

Figure S7. ^{13}C NMR spectrum ($CDCl_3$) of poly(**DEDPM**) obtained by the action of **Mo2**.

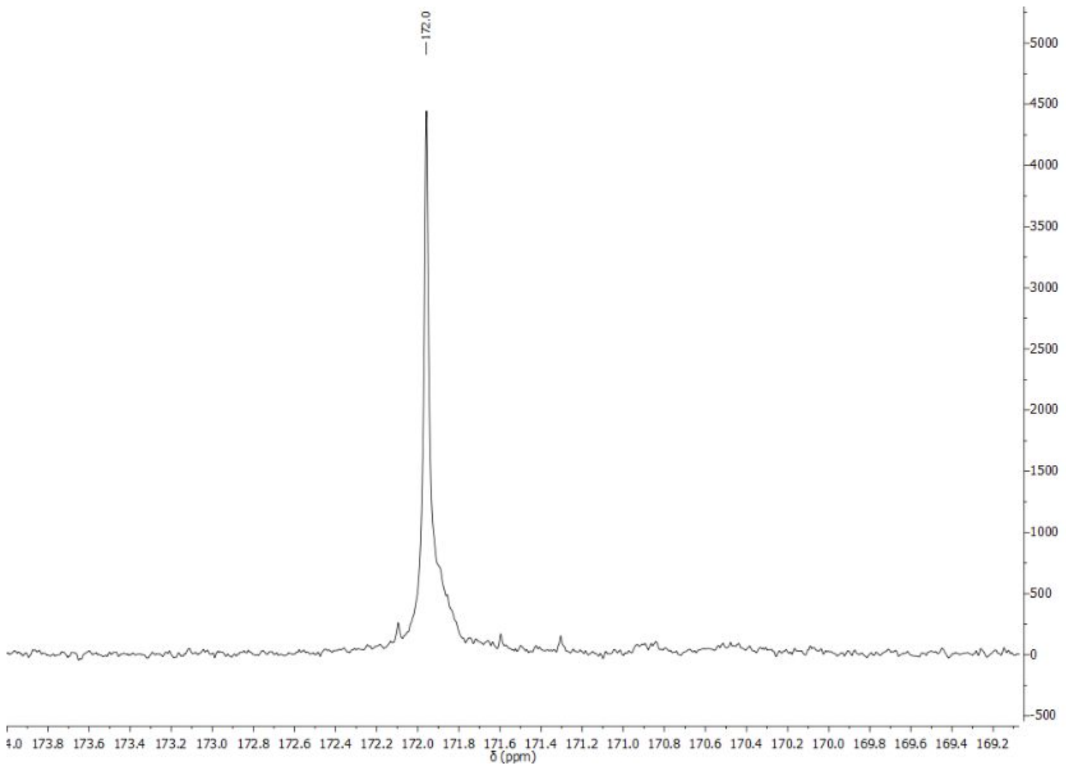

Figure S8. ^{13}C NMR spectrum ($CDCl_3$) of poly(**$^{13}C_2$-DEDPM**) obtained by the action of **Mo3**.

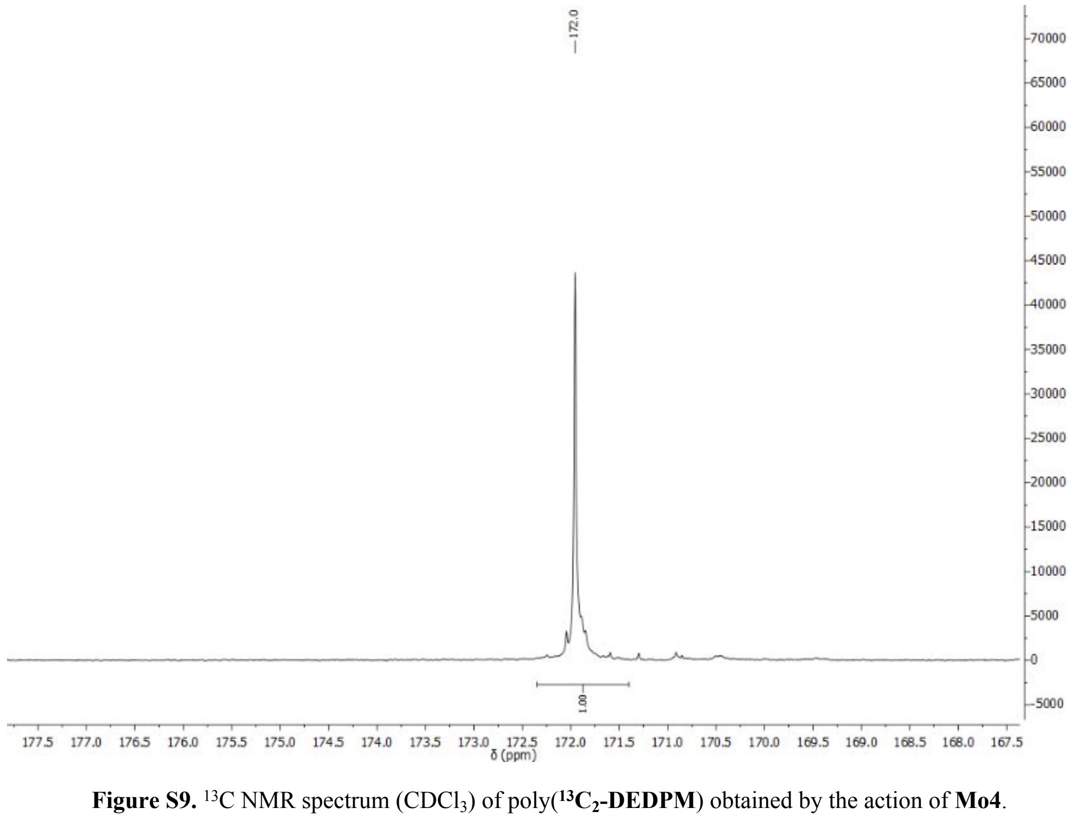

Figure S9. ^{13}C NMR spectrum ($CDCl_3$) of poly(**$^{13}C_2$-DEDPM**) obtained by the action of **Mo4**.

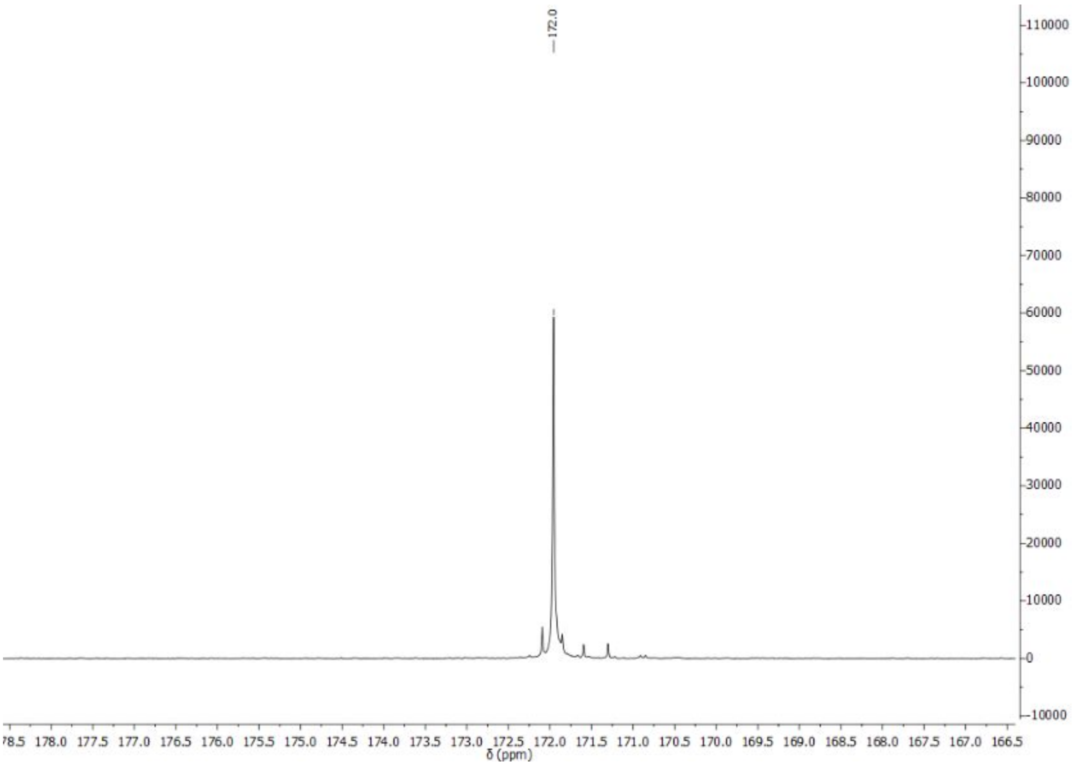

Figure S10. ^{13}C NMR spectrum ($CDCl_3$) of poly(**$^{13}C_2$-DEDPM**) obtained by the action of **Mo5**.

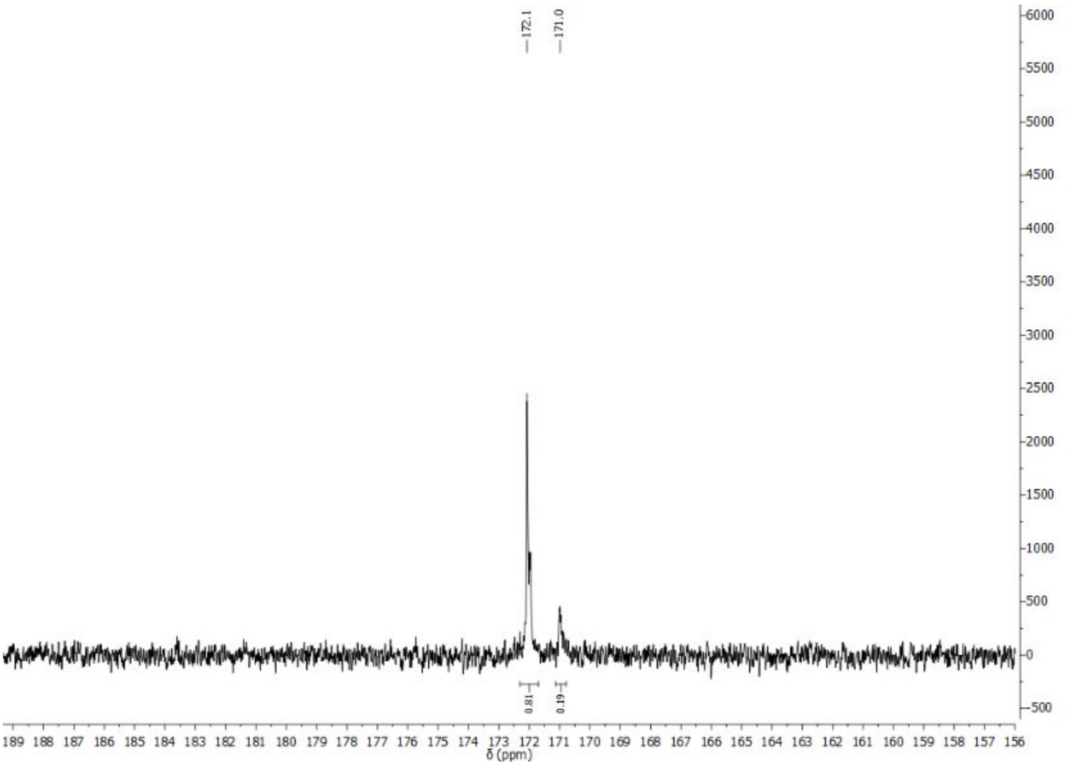

Figure S11. ^{13}C NMR spectrum ($CDCl_3$) of poly(**DEDPM**) obtained by the action of **Mo6**.

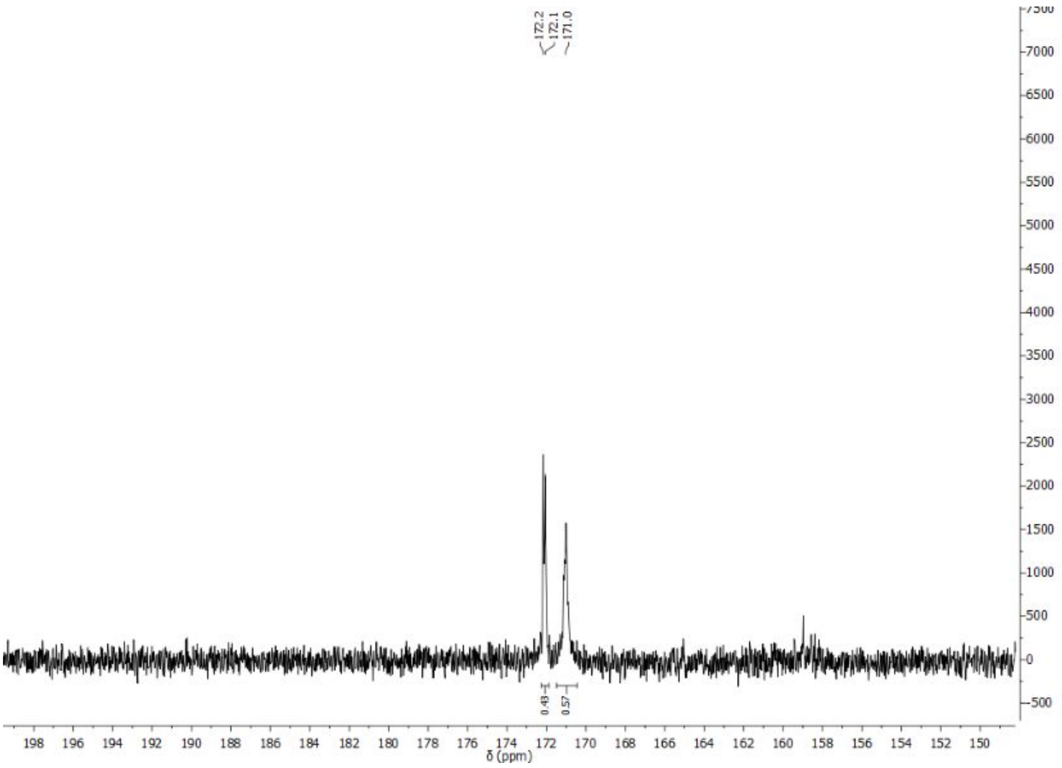

Figure S12. ^{13}C NMR spectrum ($CDCl_3$) of poly(**DEDPM**) obtained by the action of **Mo8**.

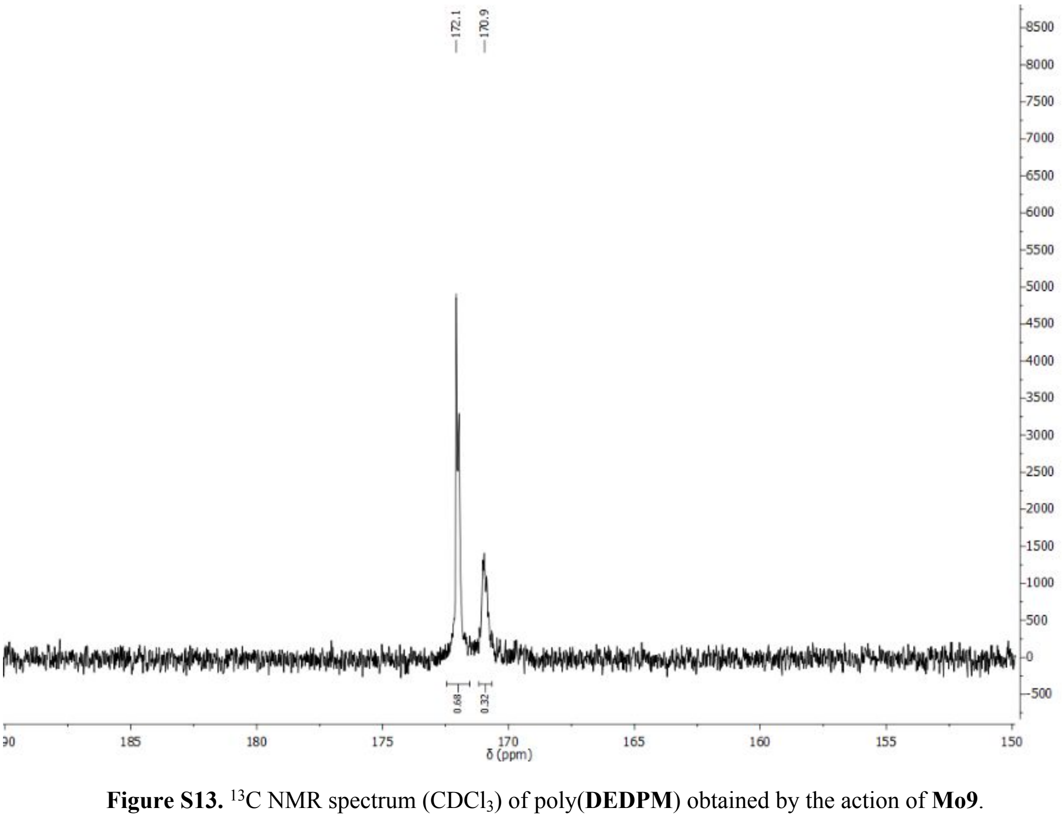

Figure S13. ^{13}C NMR spectrum ($CDCl_3$) of poly(**DEDPM**) obtained by the action of **Mo9**.

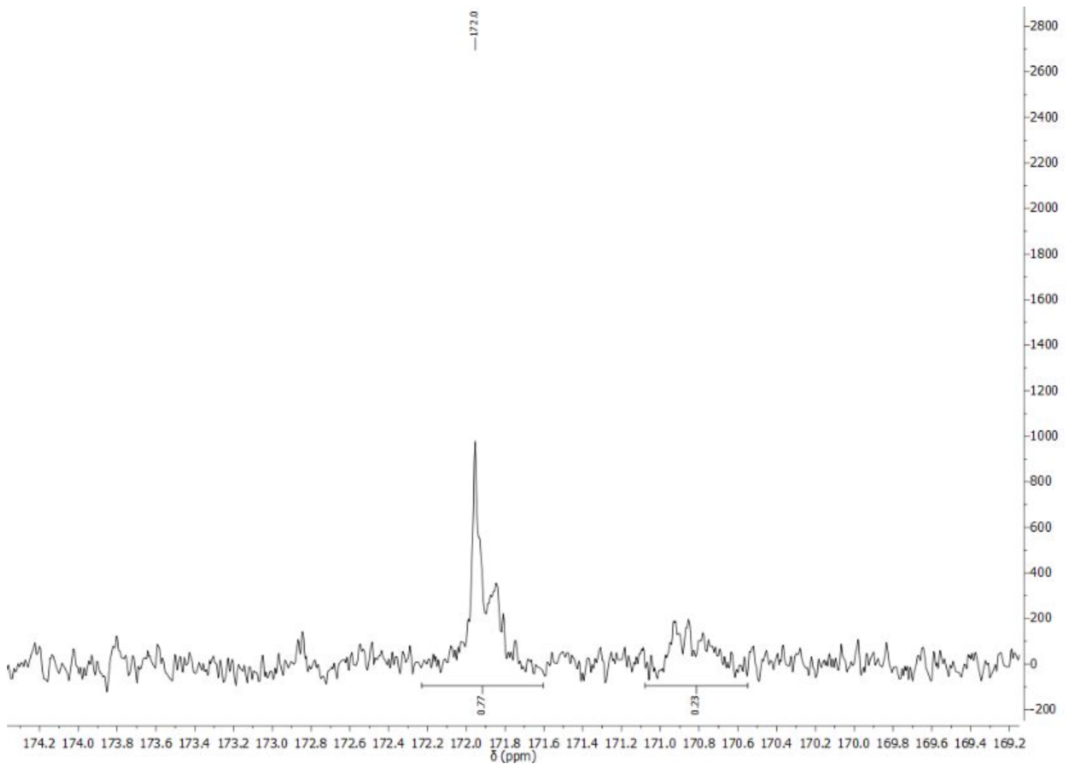

Figure S14. ^{13}C NMR spectrum ($CDCl_3$) of poly(**DEDPM**) obtained by the action of **Mo10**.

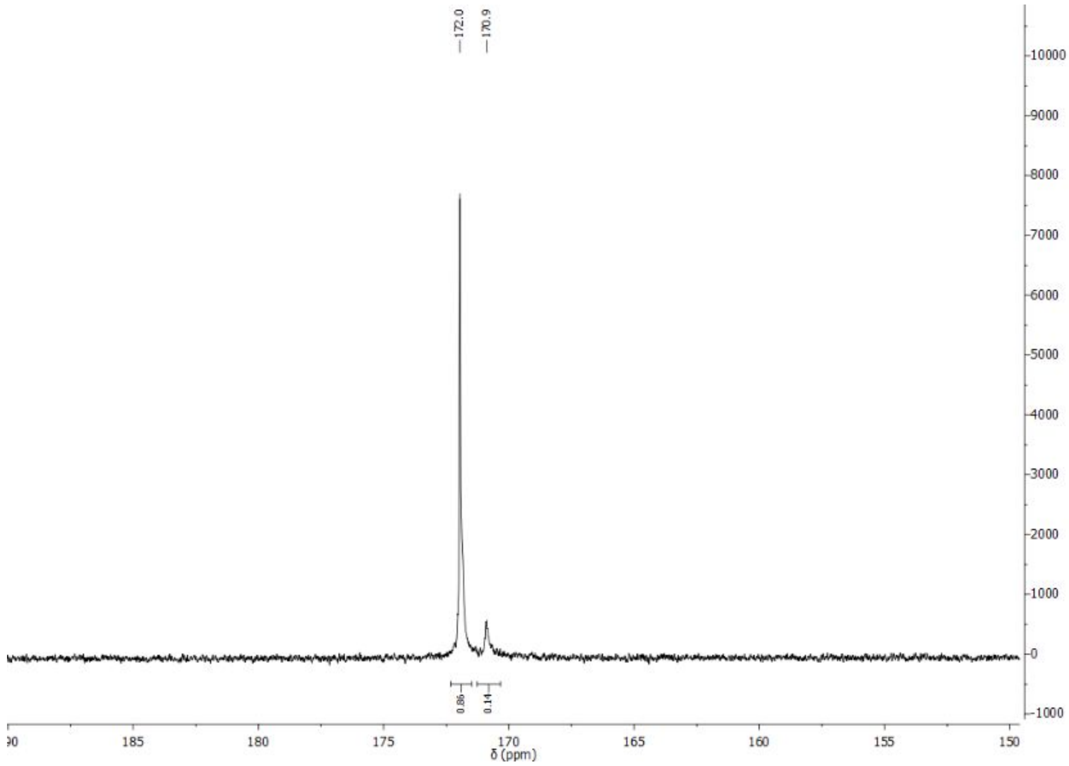

Figure S15. ^{13}C NMR spectrum ($CDCl_3$) of poly(**$^{13}C_2$-DEDPM**) obtained by the action of **Mo11**.

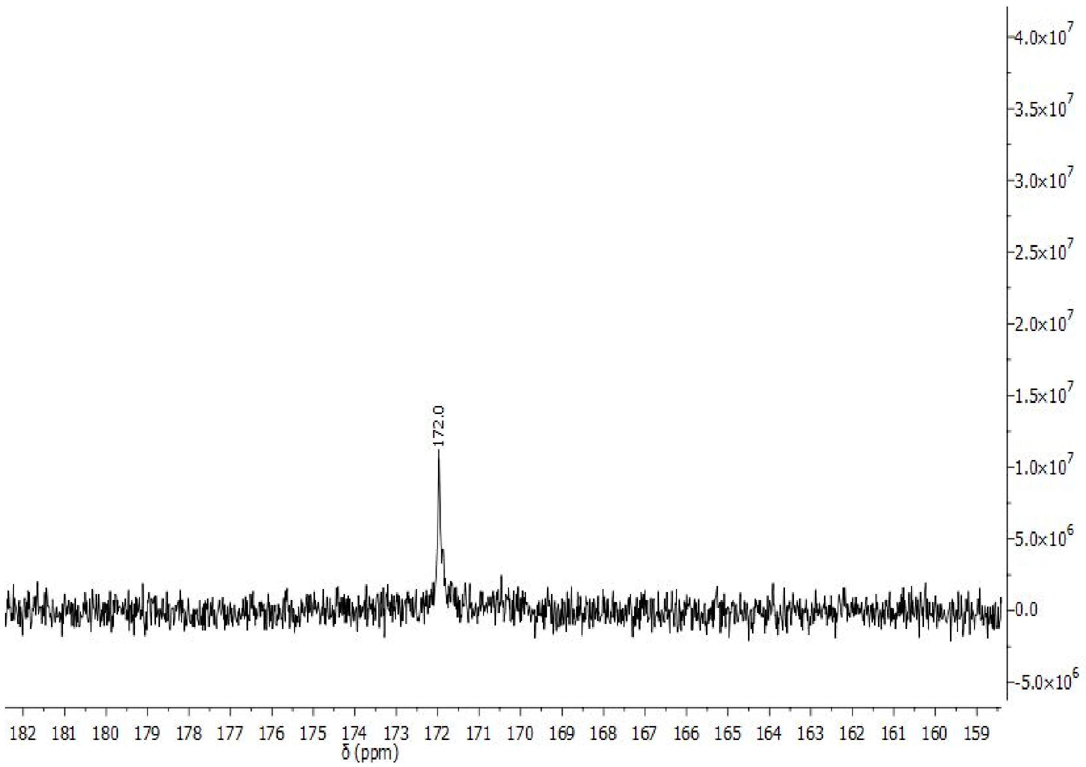

Figure S16. ^{13}C NMR spectrum ($CDCl_3$) of poly(**DEDPM**) obtained by the action of **Mo12**.

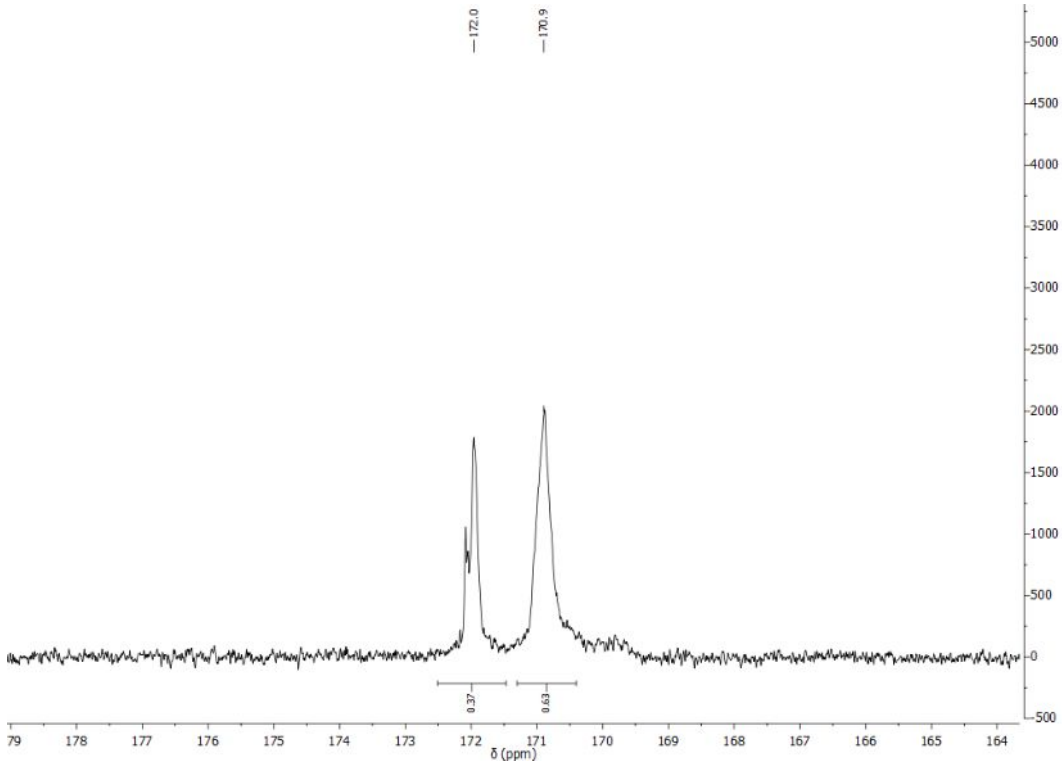

Figure S17. ^{13}C NMR spectrum ($CDCl_3$) of poly(**$^{13}C_2$-DEDPM**) obtained by the action of **Mo13**.

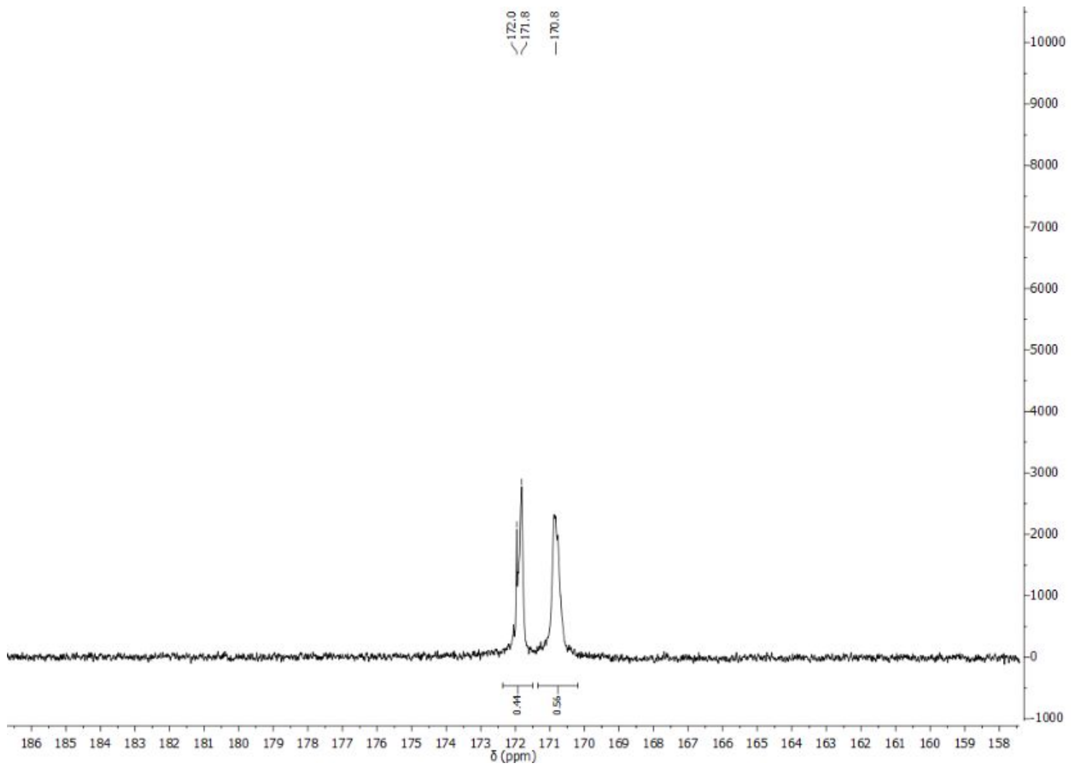

Figure S18. ^{13}C NMR spectrum ($CDCl_3$) of poly(**$^{13}C_2$-DEDPM**) obtained by the action of **Mo14**.

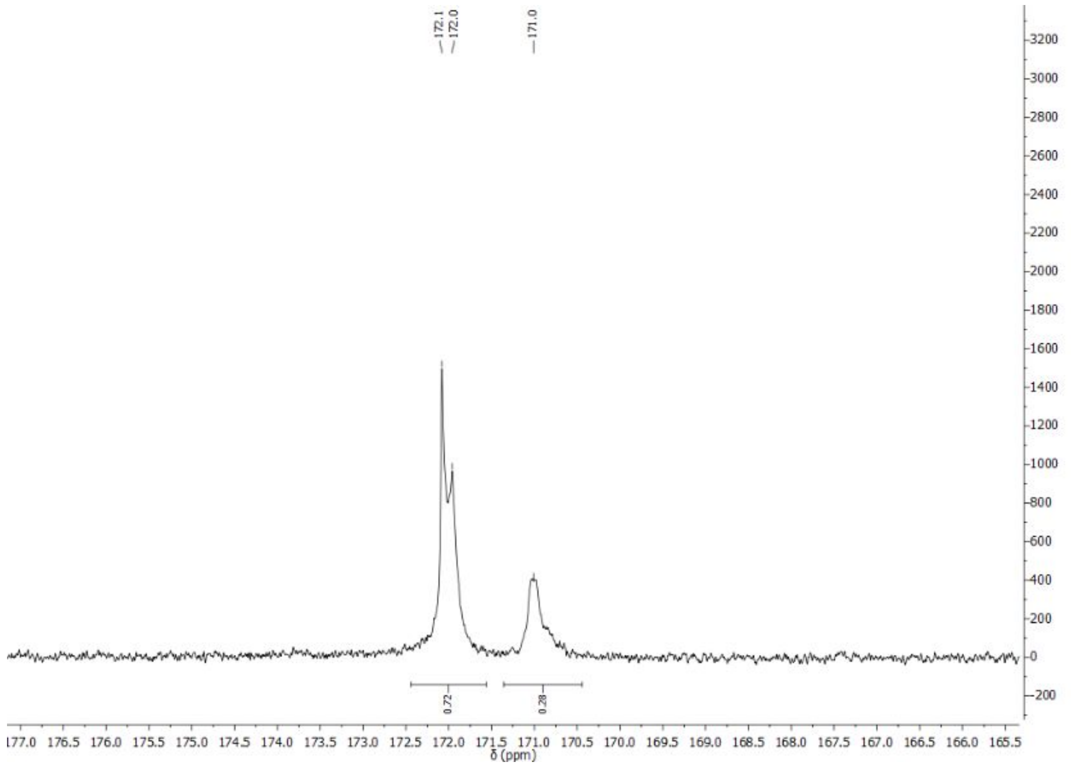

Figure S19. ^{13}C NMR spectrum ($CDCl_3$) of poly(**$^{13}C_2$-DEDPM**) obtained by the action of **Mo15**.

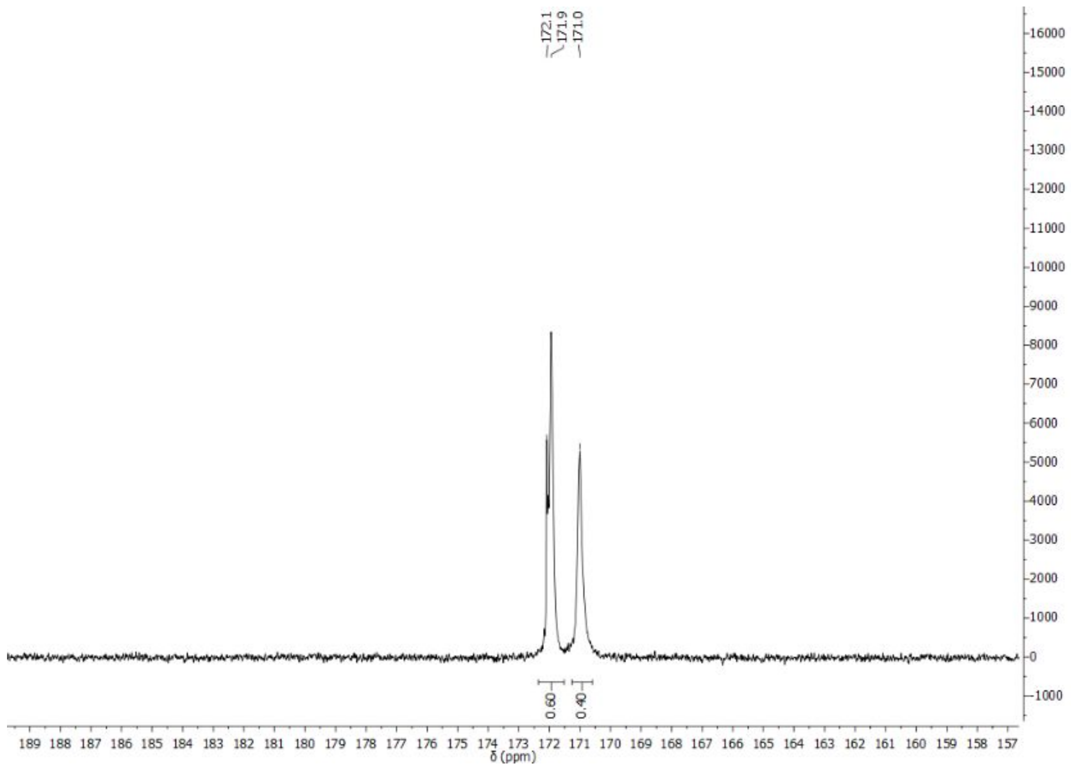

Figure S20. ^{13}C NMR spectrum ($CDCl_3$) of poly(**$^{13}C_2$-DEDPM**) obtained by the action of **Mo16**.

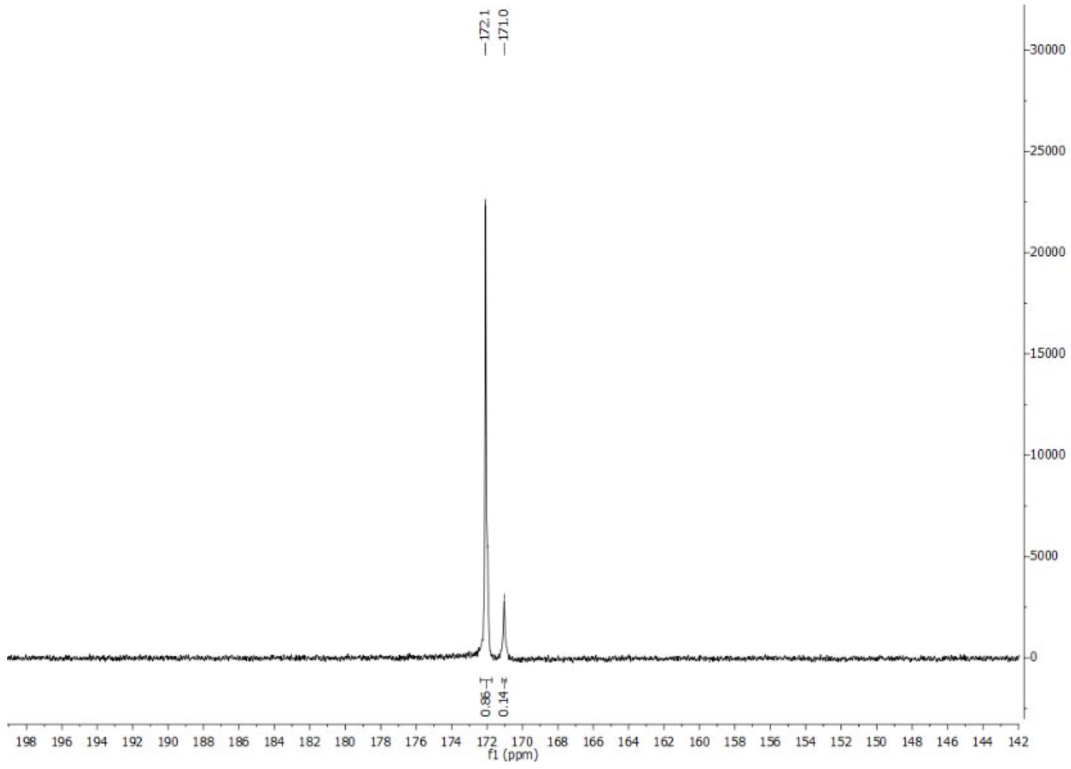

Figure S21. ^{13}C NMR spectrum ($CDCl_3$) of poly(**$^{13}C_2$-DEDPM**) obtained by the action of **Mo17**.

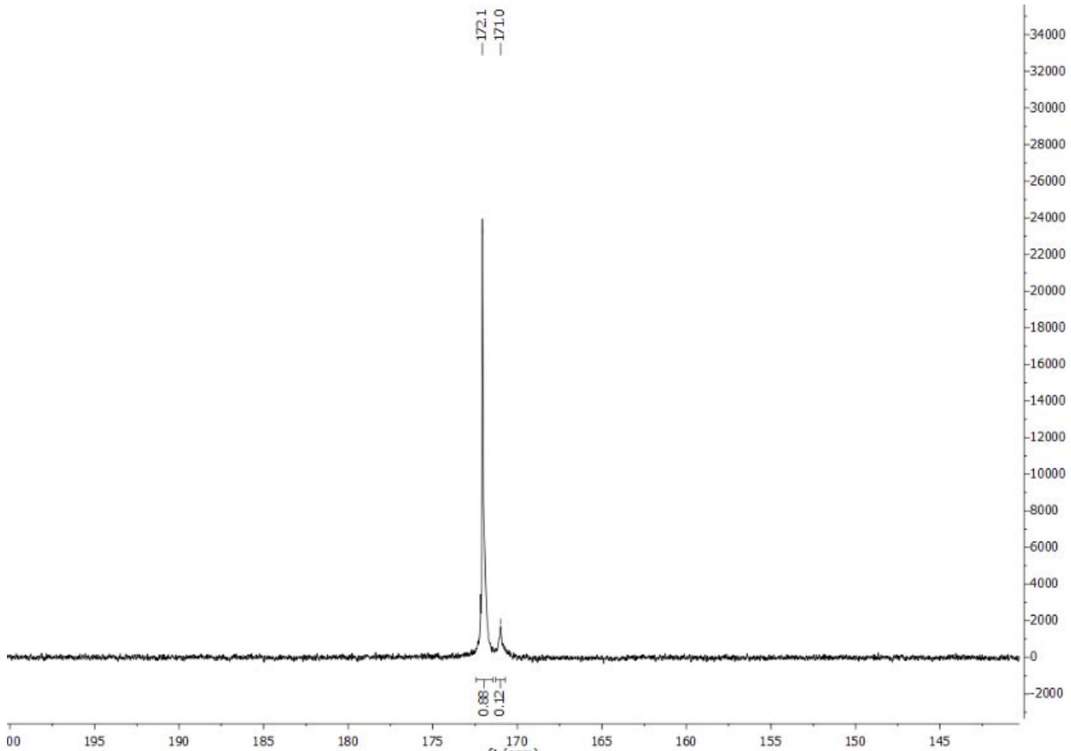

Figure S22. ^{13}C NMR spectrum ($CDCl_3$) of poly(**$^{13}C_2$-DEDPM**) obtained by the action of **Mo18**.

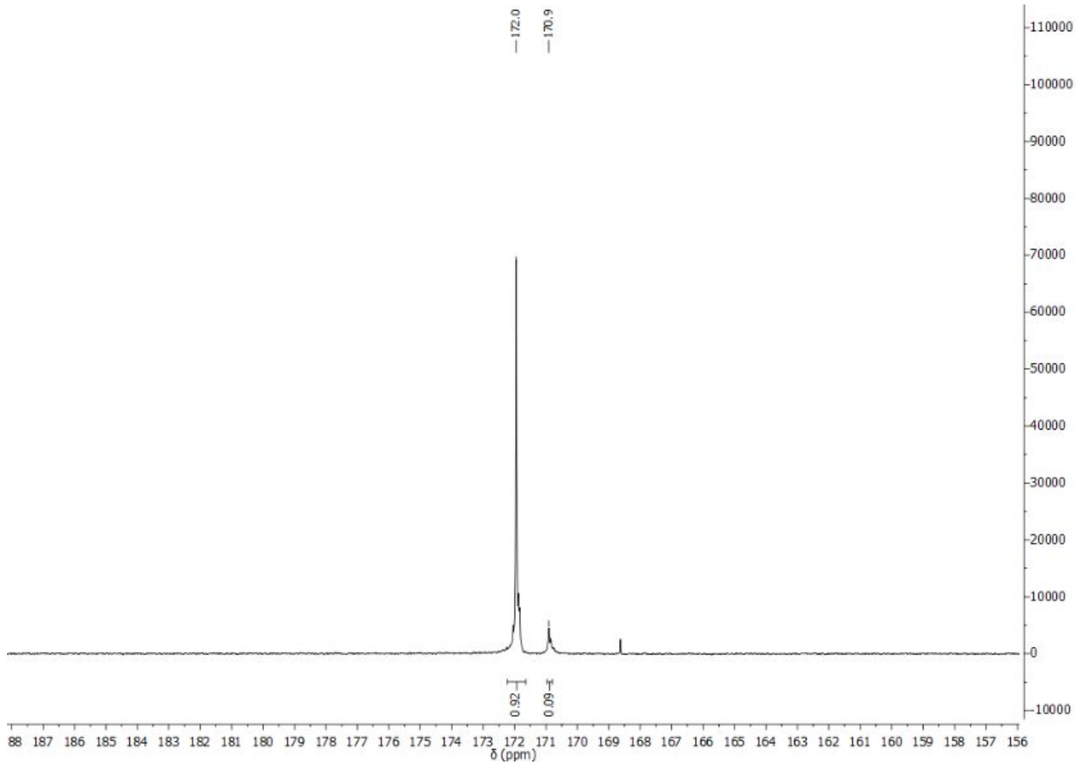

Figure S23. ^{13}C NMR spectrum ($CDCl_3$) of poly(**$^{13}C_2$-DEDPM**) obtained by the action of **Mo19**.

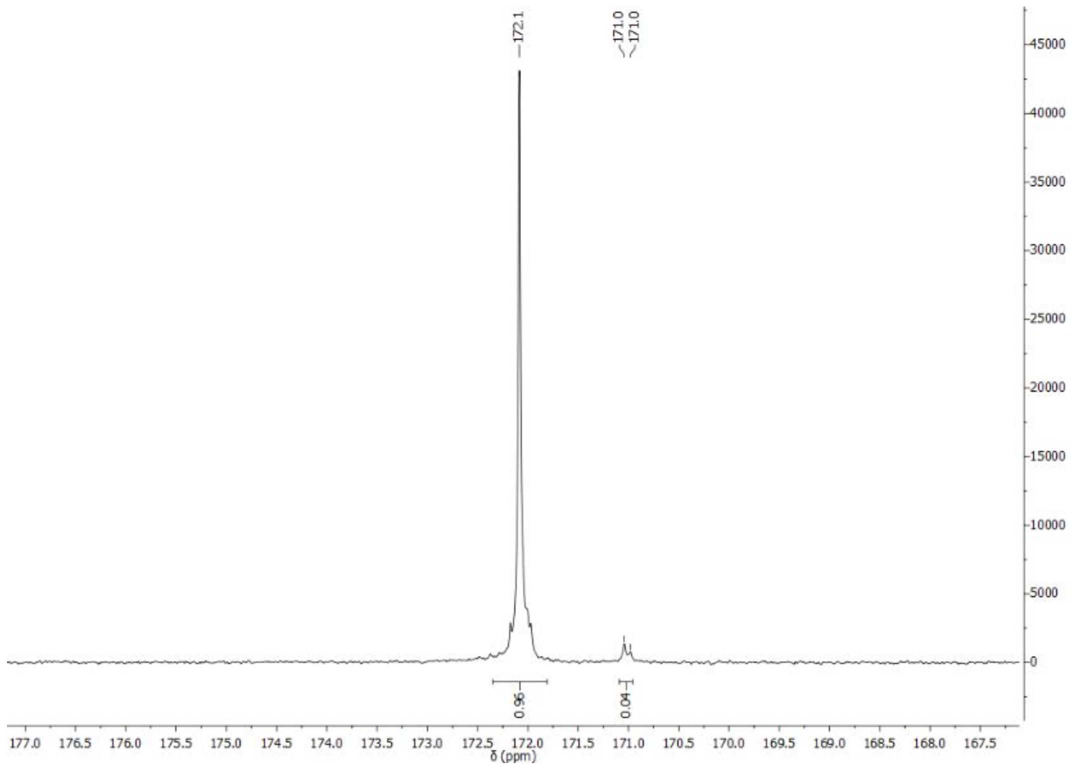

Figure S24. ^{13}C NMR spectrum ($CDCl_3$) of poly(**$^{13}C_2$-DEDPM**) obtained by the action of **Mo20**.

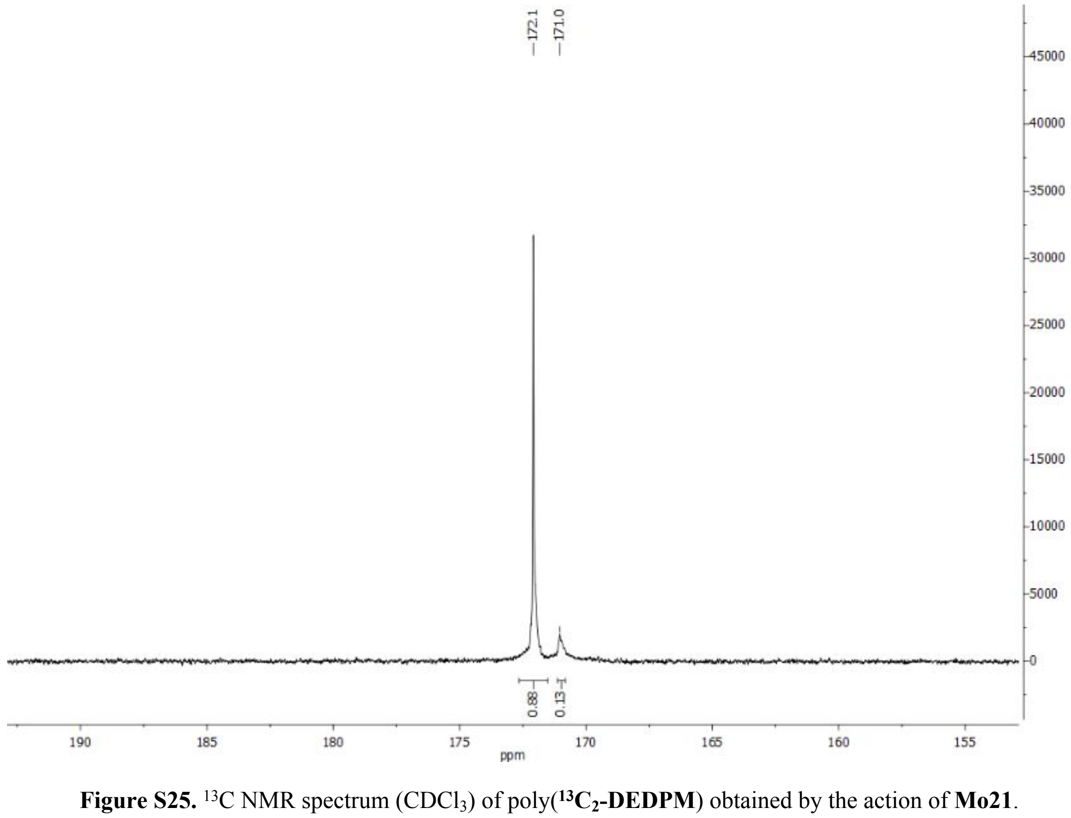

Figure S25. ^{13}C NMR spectrum ($CDCl_3$) of poly(**$^{13}C_2$-DEDPM**) obtained by the action of **Mo21**.

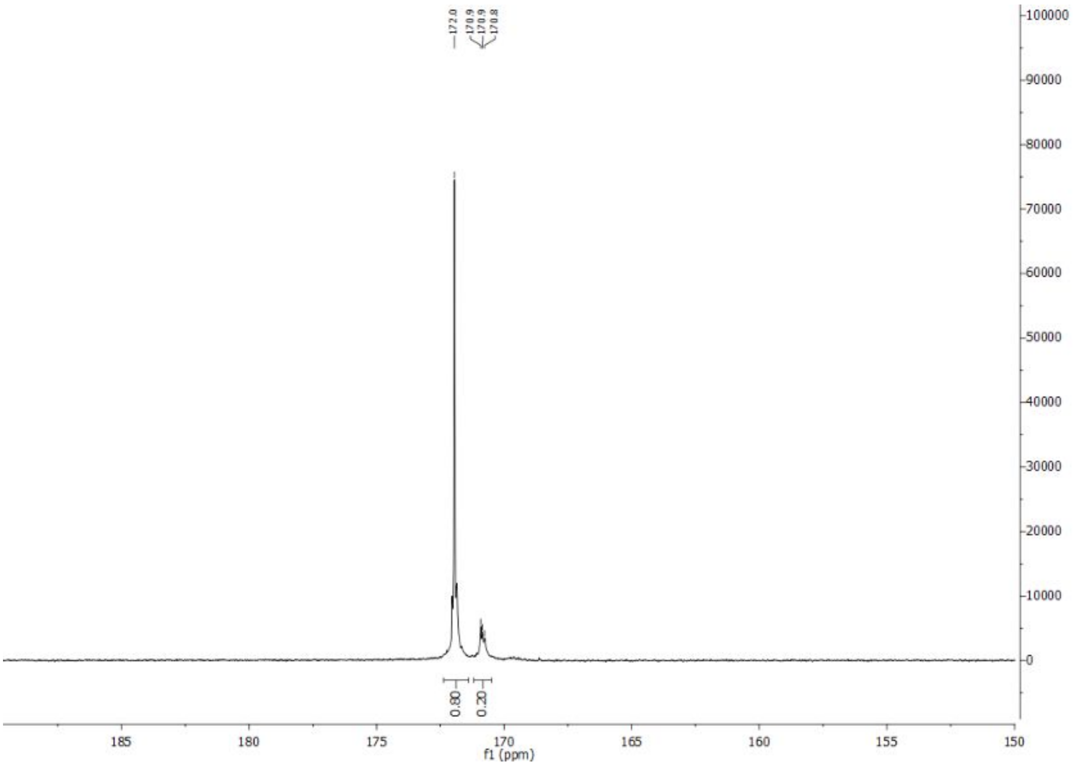

Figure S26. ^{13}C NMR spectrum ($CDCl_3$) of poly(**$^{13}C_2$-DEDPM**) obtained by the action of **Mo22**.

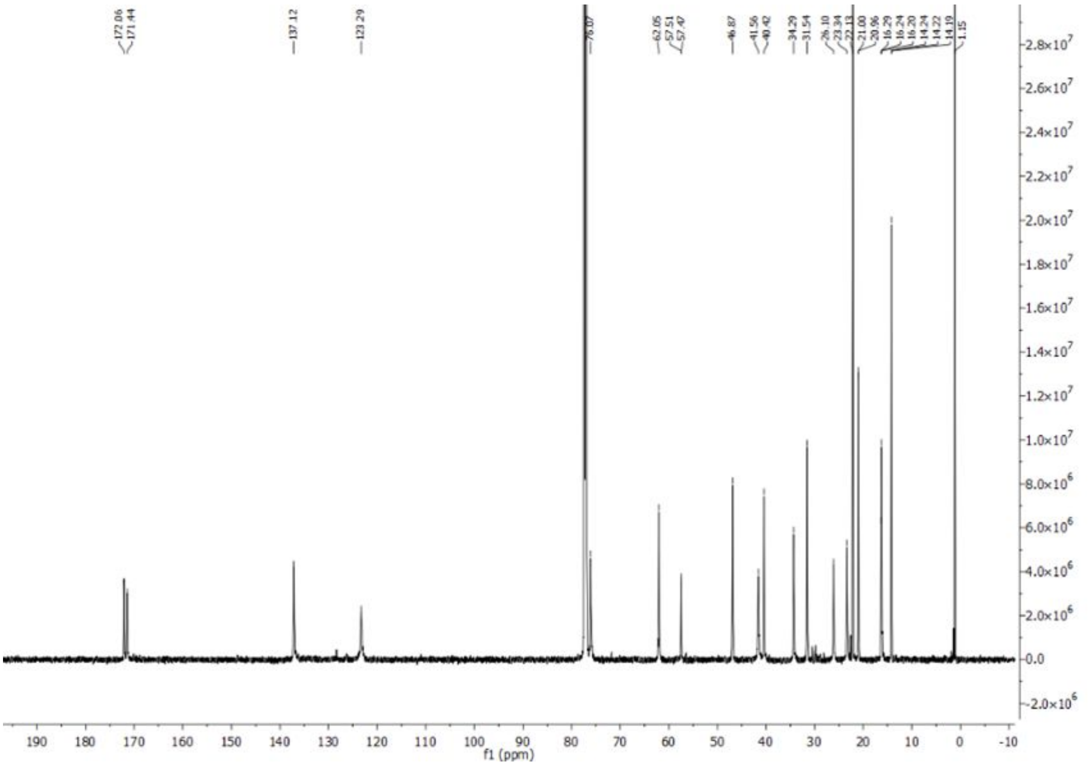

Figure S27. ^{13}C NMR spectrum ($CDCl_3$) of poly(**M2**) obtained by the action of **Mo16**.

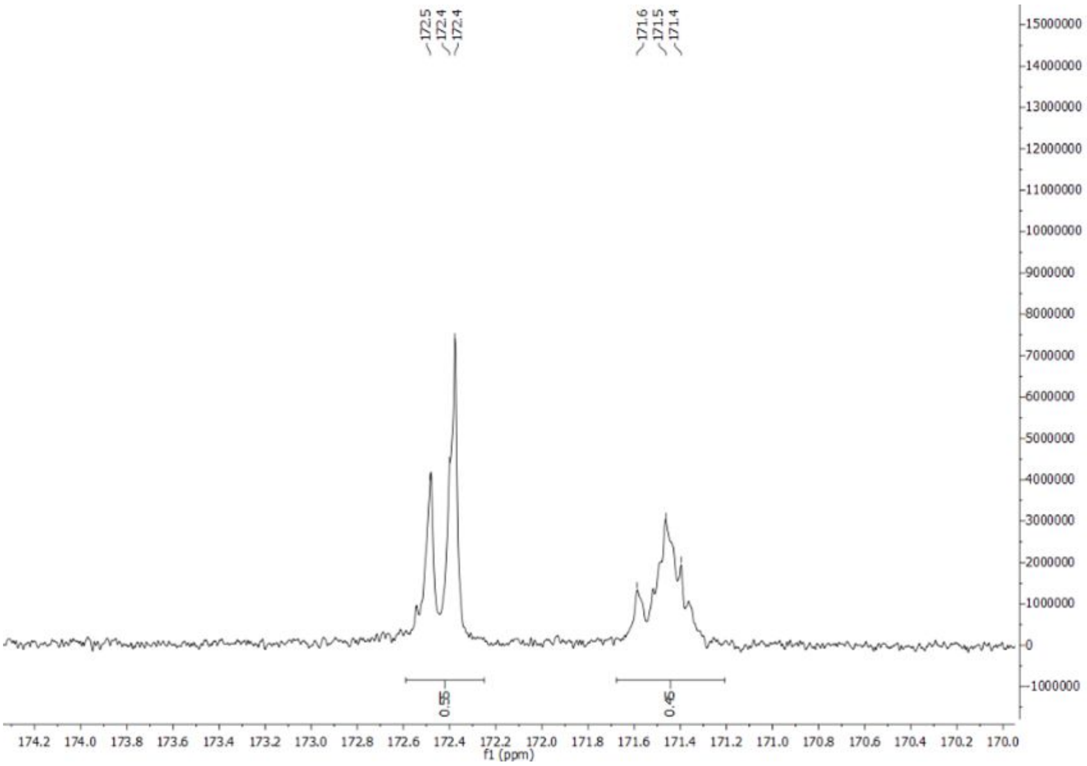

Figure S28. ^{13}C NMR spectrum ($CDCl_3$) of poly(**M3**) obtained by the action of **Mo16**.

2. UV-Vis Spectra

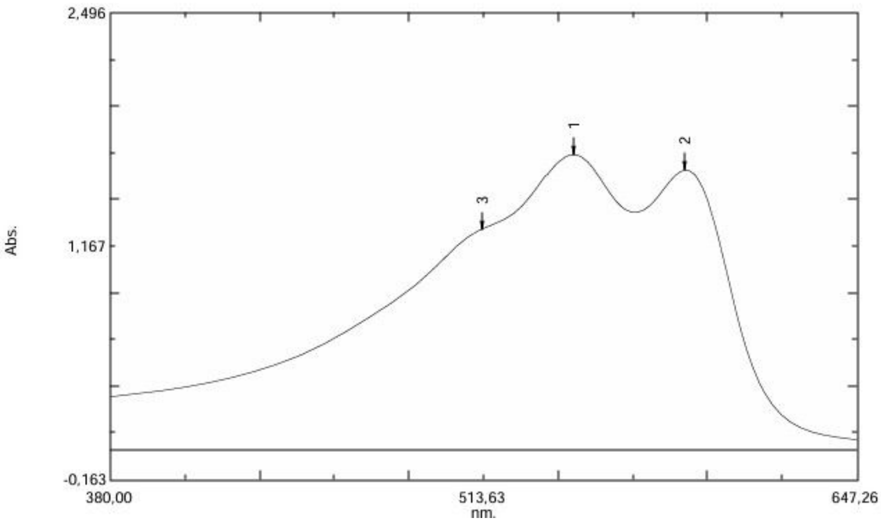

Figure S29. UV-Vis spectrum ($CHCl_3$) of poly(**DEDPM**) obtained by the action of catalyst **Mo3**. λ_1 = 546.0 nm, λ_2 = 585.5 nm, λ_3 = 513.0 nm.

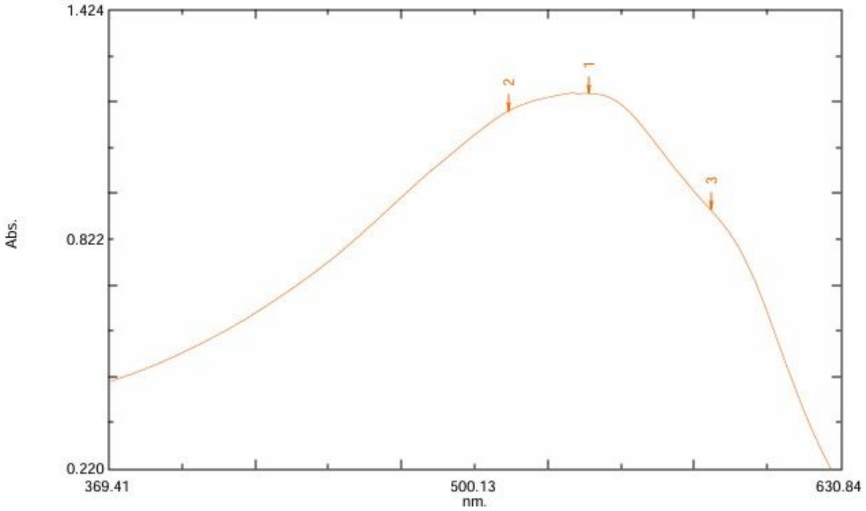

Figure S30. UV-Vis spectrum (1,2-dichloroethane) of poly(**DEDPM**) obtained by the action of catalyst **Mo8**. λ_1 = 541.0 nm, λ_2 = 512.5 nm, λ_3 = 584.5 nm.

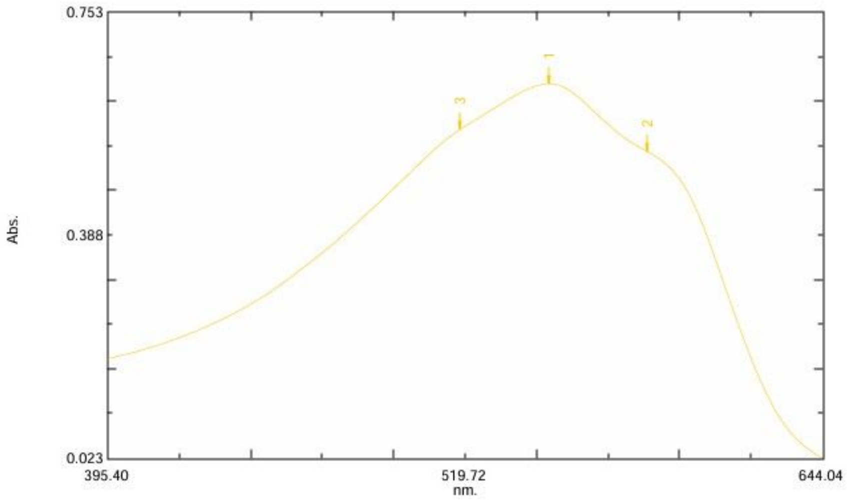

Figure S31. UV-Vis spectrum (1,2-dichloroethane) of poly(**DEDPM**) obtained by the action of catalyst **Mo9**. λ_1 = 549.0 nm, λ_2 = 583.0 nm, λ_3 = 518.0 nm.

3. Crystal Data and Structure Refinement for Mo4

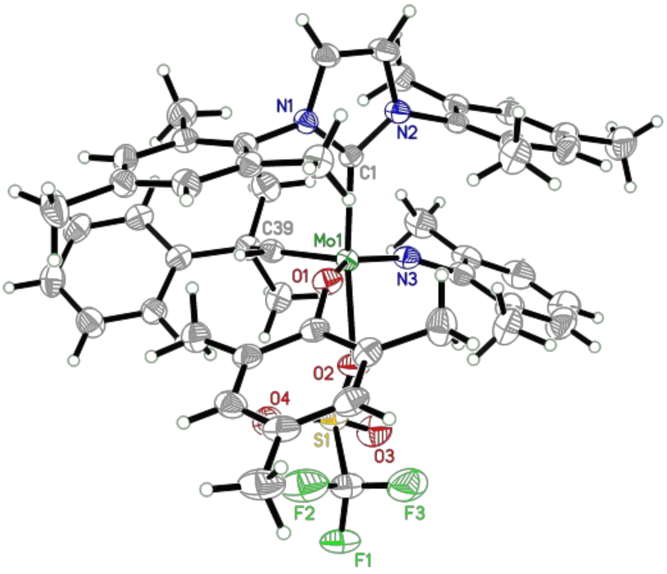

Figure S32. Single-crystal X-ray structure of **Mo4**.

Table S1. Crystal data and structure refinement for **Mo4**.

Empirical formula	$C_{51}H_{60}Cl_4F_3MoN_3O_4S$
Formula weight	1105.82
Temperature	160(2) K
Wavelength	1.54178 Å
Crystal system, space group	Triclinic, P-1
Unit cell dimensions	a = 10.8336(17) Å α = 94.411(7)°<br>b = 11.9133(18) Å β = 91.410(9)°<br>c = 20.715(3) Å γ = 96.149(7)°
Volume	2648.8(7) Å^3
Z, Calculated density	2, 1.386 Mg/m^3
Absorption coefficient	4.710 mm^{-1}
F(000)	1144
Crystal size	0.206 x 0.199 x 0.061 mm
Theta range for data collection	3.744 to 63.990°.
Limiting indices	-12<=h<=12, -10<=k<=13, -24<=l<=23
Reflections collected / unique	21688 / 8458 [R(int) = 0.0419]
Completeness to θ = 63.990	96.1 %

Absorption correction	Semi-empirical from equivalents
Max. and min. transmission	0.7528 and 0.5538
Refinement method	Full-matrix least-squares on F^2
Data / restraints / parameters	8458 / 0 / 621
Goodness-of-fit on F^2	1.043
Final R indices [I>2σ(I)]	R1 = 0.0359, wR2 = 0.0908
R indices (all data)	R1 = 0.0460, wR2 = 0.0962
Extinction coefficient	n/a
Largest diff. peak and hole	0.599 and -0.371 e.A^{-3}

4. Calculations

Table S2. Summary of structural parameters calculated using DFT-optimized structures.

Catalyst	$d_{O\text{-}Mo,4r\text{-}1\alpha}$ [Å]	$d_{O\text{-}Mo,4r\text{-}2\beta}$ [Å]	$V_{bur,NHC,4r\text{-}2\alpha}$ [%]	$V_{bur,X,4r\text{-}2\alpha}$ [%]	$V_{bur,NHC,[Au]}$ [%]	$V_{bur,X,[Au]}$ [%]	$CM5_{OCoord,4r\text{-}1\alpha}$	$V_{bur,Poly,4r\text{-}2\alpha}$ [%]	α-Selectivity
**Mo1**	2.68	3.67	32.0	14.6	39.5	18.5	-0.297827	34.3	90
**Mo2**	2.43	3.53	31.0	14.7	39.5	18.5	-0.302264	33.3	88
**Mo3**	2.39	3.81	31.6	14.6	39.5	18.5	-0.302324	34.2	98
**Mo4**	3.31	4.62	31.5	18.4	39.5	21.0	-0.292446	32.9	99
**Mo5**	3.09	3.65	33.5	16.0	39.5	16.3	-0.298538	33.2	99
**Mo6**	2.31	3.08	33.9	14.5	40.5	18.5	-0.305378	33.6	81
**Mo7**	2.71	3.68	33.8	17.3	40.5	18.2	-0.296007	31.6	96
**Mo8**	2.94	2.73	25.8	16.0	27.8	18.5	-0.2885	30.9	43
**Mo9**	2.77	3.15	30.4	15.3	27.8	16.3	-0.291893	30.4	68
**Mo10**	2.36	3.72	28.9	14.9	29.8	18.5	-0.305218	34.6	77
**Mo11**	2.93	4.81	28.5	25.2	26.3	38.6	-0.296061	32.6	86
**Mo13**	2.72	4.72	38.6	15.6	45.7	16.3	-0.293644	32.2	37
**Mo14**	2.73	2.96	37.0	15.7	45.7	16.3	-0.293324	32.9	44
**Mo15**	2.68	3.12	37.1	19.5	45.7	21.8	-0.300298	31.8	72
**Mo16**	2.92	4.34	35.2	14.7	42.4	16.3	-0.291789	32.3	60
**Mo17**	2.75	4.03	35.3	14.8	42.4	16.3	-0.295007	31.0	86
**Mo19**	2.58	3.15	33.7	11.7	42.4	9.7	-0.297094	32.9	92
**Mo21**	2.61	3.52	34.2	13.6	42.4	18.5	-0.294178	33.0	88
**Mo22**	2.58	3.29	33.2	11.8	42.4	9.7	-0.296745	34.0	80

Table S3. Summary of calculated "reduced α-selectivities" used in **Figure 6** and **Figure 8**.

Catalyst	$Q_{Ocoord,4r-1\alpha}$	$Q_{Ocoord,4r-1\alpha}$ scaled to [0;1]	α-selectivity (%)		
			experimental	reduced (Mo-complexes)	reduced (Au-complexes)
**Mo1**	-0.297827	0.447387131	90	78.0747987139028	70.6896001916067
**Mo2**	-0.302264	0.184500533	88	77.9629811416364	73.6192273375001
**Mo3**	-0.302324	0.18094561	98	88.0884824081579	83.8112073595340
**Mo4**	-0.292446	0.766204527	99	104.3585985114710	98.2484601673742
**Mo5**	-0.298538	0.405261287	99	86.3958467817956	79.2491211135910
**Mo6**	-0.305378	0	81	81.0000000000000	81.0000000000000
**Mo7**	-0.296007	0.555219813	96	87.5194367971777	79.9565877848546
**Mo8**	-0.2885	1	43	74.6691421759344	72.9519055030536
**Mo9**	-0.291893	0.798969072	68	76.3506305218175	70.6798353508188
**Mo10**	-0.305218	0.009479796	77	76.3091766935921	76.0415850031443
**Mo11**	-0.296061	0.552020382	86	77.3819009846399	69.8153632000020
**Mo13**	-0.293644	0.695224553	37	36.6539592223234	29.7891278167476
**Mo14**	-0.293324	0.714184145	44	45.0736405997937	38.3839996597201
**Mo15**	-0.300298	0.300983529	72	59.3264634023380	53.1786475316573
**Mo16**	-0.291789	0.80513094	60	68.9386427739317	63.3568923317674
**Mo17**	-0.295007	0.614468539	86	80.4569022166701	73.0613901214001
**Mo19**	-0.297094	0.490816447	92	81.1669143934894	73.6353087070038
**Mo21**	-0.294178	0.663585733	88	85.4538267978687	78.3394921912615
**Mo22**	-0.296745	0.511494253	80	69.8268056151065	62.2609700352582

Table S4. All relevant parameters for the fit function used to approximate the α-selectivity (Equation 1).

**Variable**	**Coefficient**
Intercept	71.46
$V_{bur,NHC,2a}$	47.204
$(V_{bur,NHC,2a})^3$	-82.123
$CM5charge_{OCoord,1a}$	73.874
$(CM5charge_{OCoord,1a})^2$	-105.543
RMSE	7.239
R^2	0.854
R^2_{adj}	0.812

Table S5. All relevant parameters for the cross-validated fit function (Equation 3).

**Variable**	**Coefficient**
Intercept	70.771 (4.361)
$V_{bur,NHC,2a}$	49.539 (12.776)
$(V_{bur,NHC,2a})^3$	-84.274 (11.875)
$CM5charge_{OCoord,1a}$	74.745 (16.0)
$(CM5charge_{OCoord,1a})^2$	-106.88 (19.606)
RMSE	9.707 (3.52)
R^2	-0.013 (1.845)
R^2_{adj}	-0.302

Table S6. All relevant parameters for the fit function based partially on the gold complexes (Equation 4).

Variable	**Coefficient**
Intercept	67.44
$V_{bur,[(NHC)AuCl]}$	62.144
$(V_{bur,[(NHC)AuCl]})^3$	-88.582
$CM5charge_{OCoord,1a}$	102.355
$(CM5charge_{OCoord,1a})^2$	-132.307
RMSE	7.21
R^2	0.855
R^2_{adj}	0.814

Table S7. All relevant parameters for the fit function that also includes the buried volume of the imido and X ligand.

**Variable**	**Coefficient**
Intercept	41.16
$V_{bur,NHC,2a}$	99.966
$(V_{bur,NHC,2a})^3$	-117.128
$V_{bur,Imido,2a}$	19.629
$(V_{bur,Imido,2a})^2$	-9.899
$V_{bur,X,2a}$	38.737

$(V_{bur,X,2a})^2$	-6.952
$CM5charge_{OCoord,1a}$	13.455
$(CM5charge_{OCoord,1a})^2$	-34.497
RMSE	6.679
R^2	0.876
R^2_{adj}	0.776

Table S8. All relevant parameters for the fit function that also includes the partial charge of the NHC, imido, and X ligand.

Variable	Coefficient
Intercept	65.67
$V_{bur,NHC,2a}$	46.538
$(V_{bur,NHC,2a})^3$	-78.6
$Q_{OCoord,1a}$	44.857
$(Q_{OCoord,1a})^2$	-77.139
$Q_{NHC,2a}$	57.145
$(Q_{NHC,2a})^2$	-57.01
$Q_{Imido,2a}$	-9.263
$(Q_{Imido,2a})^2$	8.837
$Q_{X,2a}$	10.98
$(Q_{X,2a})^2$	-6.46
RMSE	6.507
R^2	0.882
R^2_{adj}	0.734

Table S9. Calculated partial charges.

Mo1 4r-1α

1	Mo	0.651131
2	N	-0.153037
3	C	-0.009833
4	N	0.028957
5	C	0.010811
6	H	0.074547
7	N	0.019535
8	C	0.006087
9	H	0.073127
10	C	0.020913
11	C	0.000459
12	C	-0.044861
13	H	0.039979
14	C	0.02167
15	C	-0.03452
16	H	0.043749
17	C	0.00224
18	C	0.024251
19	C	0.008598
20	C	-0.040881
21	H	0.040867
22	C	0.021268
23	C	-0.040264
24	H	0.040418
25	C	0.006384
26	C	-0.083119
27	H	0.042441
28	H	0.030531
29	H	0.021315
30	C	-0.073451
31	H	0.037923
32	H	0.04462
33	H	0.034499
34	C	-0.081636
35	H	0.04044
36	H	0.036723
37	H	0.026474
38	C	0.038543
39	C	0.02344
40	C	-0.030971
41	H	0.044808
42	C	-0.011502
43	H	0.051078
44	C	-0.02637

45	H	0.051466
46	C	0.026882
47	C	-0.081867
48	H	0.037727
49	H	0.021783
50	H	0.038327
51	C	-0.07991
52	H	0.040185
53	H	0.02253
54	H	0.040867
55	C	-0.085638
56	H	0.030623
57	C	-0.014295
58	C	-0.071386
59	C	-0.012985
60	C	0.019324
61	C	-0.045657
62	C	0.009115
63	C	-0.057984
64	C	-0.073685
65	C	0.214787
66	C	0.216211
67	O	-0.116798
68	O	-0.254627
69	O	-0.120865
70	O	-0.255234
71	C	0.043457
72	C	-0.078646
73	C	0.048827
74	C	-0.079594
75	H	0.028195
76	H	0.014703
77	H	0.034848
78	H	0.038631
79	H	0.03838
80	H	0.04439
81	H	0.045669
82	H	0.040163
83	H	0.032482
84	H	0.034834
85	H	0.043406
86	H	0.027692
87	H	0.041156
88	H	0.0386
89	H	0.027475
90	H	0.041184

91	H	0.037002
92	C	-0.035911
93	C	0.014491
94	C	0.235235
95	C	0.215296
96	C	-0.024193
97	C	-0.046252
98	C	-0.087511
99	O	-0.246834
100	O	-0.108421
101	O	-0.22401
102	O	-0.084415
103	C	0.046719
104	C	-0.081782
105	C	0.053556
106	C	-0.077423
107	H	0.083333
108	H	0.052374
109	H	0.047068
110	H	0.093352
111	H	0.041726
112	H	0.048528
113	H	0.048883
114	H	0.032113
115	H	0.03567
116	H	0.042102
117	H	0.040952
118	H	0.03019
119	H	0.044096
120	H	0.03201
121	H	0.044196
122	H	0.031885
123	C	-0.072487
124	H	0.042824
125	H	0.037892
126	H	0.03896
127	C	-0.08545
128	H	0.029822
129	H	0.038855
130	H	0.026319
131	C	-0.078262
132	H	0.044628
133	H	0.025041
134	H	0.025264
135	O	-0.311351
136	S	0.503645

137	O	-0.319901
138	O	-0.31142
139	C	0.291446
140	F	-0.084279
141	F	-0.082453
142	F	-0.075205
	Tot	1.000039

Mo2 4r-1α

1	Mo	0.65191
2	N	-0.152278
3	C	-0.008718
4	N	0.025203
5	C	0.008296
6	H	0.073793
7	N	0.018791
8	C	0.004908
9	H	0.0723
10	C	0.02047
11	C	0.000328
12	C	-0.040088
13	H	0.040235
14	C	0.026135
15	C	-0.036153
16	H	0.040182
17	C	0.002798
18	C	0.027159
19	C	0.012758
20	C	-0.03844
21	H	0.041795
22	C	0.022509
23	C	-0.041047
24	H	0.039843
25	C	0.009558
26	C	-0.087576
27	H	0.035695
28	H	0.038401
29	H	0.017181
30	C	-0.071902
31	H	0.039237
32	H	0.045311
33	H	0.038373
34	C	-0.085104
35	H	0.035805
36	H	0.036351
37	H	0.022054

38	C	0.045162
39	C	0.024872
40	C	-0.029978
41	H	0.043129
42	C	-0.013092
43	H	0.051023
44	C	-0.019635
45	H	0.055277
46	C	-0.016637
47	C	0.028134
48	C	-0.098079
49	H	0.02796
50	C	-0.016919
51	C	-0.065629
52	C	-0.0126
53	C	0.013877
54	C	-0.04748
55	C	0.008835
56	C	-0.059643
57	C	-0.07625
58	C	0.214758
59	C	0.216279
60	O	-0.117237
61	O	-0.255512
62	O	-0.120182
63	O	-0.256119
64	C	0.043171
65	C	-0.079042
66	C	0.048233
67	C	-0.079426
68	H	0.027574
69	H	0.013854
70	H	0.034509
71	H	0.035852
72	H	0.035633
73	H	0.042964
74	H	0.045149
75	H	0.039416
76	H	0.033086
77	H	0.034818
78	H	0.043039
79	H	0.027873
80	H	0.041117
81	H	0.038363
82	H	0.025965
83	H	0.041323

84	H	0.033867
85	C	-0.036737
86	C	0.01576
87	C	0.24549
88	C	0.215783
89	C	-0.021431
90	C	-0.046171
91	C	-0.08515
92	O	-0.246733
93	O	-0.106705
94	O	-0.209736
95	O	-0.077061
96	C	0.047931
97	C	-0.082304
98	C	0.057529
99	C	-0.076664
100	H	0.083862
101	H	0.054901
102	H	0.048858
103	H	0.093948
104	H	0.04347
105	H	0.048993
106	H	0.046278
107	H	0.036649
108	H	0.036279
109	H	0.043281
110	H	0.039807
111	H	0.03215
112	H	0.044848
113	H	0.031589
114	H	0.044054
115	H	0.031973
116	C	-0.070739
117	H	0.045753
118	H	0.038846
119	H	0.037823
120	C	-0.087333
121	H	0.032406
122	H	0.036613
123	H	0.023196
124	C	-0.082956
125	H	0.041715
126	H	0.025517
127	H	0.021043
128	O	-0.307423
129	S	0.506326

130	O	-0.305576
131	O	-0.306525
132	C	0.291543
133	F	-0.084749
134	F	-0.0805
135	F	-0.075506
136	C	-0.077525
137	H	0.033909
138	H	0.030114
139	H	0.029361
140	C	-0.083074
141	H	0.032651
142	H	0.030197
143	H	0.016387
144	C	-0.095376
145	H	0.006469
146	H	0.024648
147	H	0.016432
148	H	0.044016
	Tot	1.000148

Mo3 4r-1α

1	Mo	0.645828
2	N	-0.181082
3	C	-0.005569
4	N	0.028696
5	C	0.009225
6	H	0.073261
7	N	0.017257
8	C	0.004749
9	H	0.072536
10	C	0.022157
11	C	-0.001245
12	C	-0.046183
13	H	0.039953
14	C	0.020167
15	C	-0.034669
16	H	0.044364
17	C	0.003722
18	C	0.022838
19	C	0.004565
20	C	-0.046535
21	H	0.039434
22	C	0.016919
23	C	-0.044205
24	H	0.039456

25	C	0.003989
26	C	-0.08564
27	H	0.021581
28	H	0.041427
29	H	0.022346
30	C	-0.074152
31	H	0.037287
32	H	0.045561
33	H	0.034704
34	C	-0.087112
35	H	0.037203
36	H	0.037607
37	H	0.018846
38	C	0.03314
39	C	0.041068
40	C	-0.028889
41	H	0.053709
42	C	-0.00059
43	H	0.060343
44	C	-0.02239
45	H	0.060348
46	C	0.04276
47	C	-0.086445
48	H	0.031361
49	C	-0.019685
50	C	-0.074823
51	C	-0.012773
52	C	0.010671
53	C	-0.04767
54	C	0.008243
55	C	-0.06109
56	C	-0.077823
57	C	0.213149
58	C	0.216519
59	O	-0.117637
60	O	-0.258176
61	O	-0.120907
62	O	-0.25496
63	C	0.044228
64	C	-0.079121
65	C	0.048594
66	C	-0.079673
67	H	0.027935
68	H	0.005947
69	H	0.030366
70	H	0.035999

71	H	0.029771
72	H	0.042602
73	H	0.04508
74	H	0.039938
75	H	0.032474
76	H	0.035079
77	H	0.042975
78	H	0.027878
79	H	0.042497
80	H	0.039879
81	H	0.02454
82	H	0.040633
83	H	0.036422
84	C	-0.038769
85	C	0.014505
86	C	0.244339
87	C	0.215458
88	C	-0.023585
89	C	-0.046676
90	C	-0.086258
91	O	-0.246969
92	O	-0.106146
93	O	-0.210445
94	O	-0.074541
95	C	0.04804
96	C	-0.081175
97	C	0.056864
98	C	-0.07612
99	H	0.077769
100	H	0.054276
101	H	0.048208
102	H	0.093965
103	H	0.036273
104	H	0.050207
105	H	0.051037
106	H	0.032302
107	H	0.037736
108	H	0.044063
109	H	0.041301
110	H	0.03152
111	H	0.045753
112	H	0.032215
113	H	0.045203
114	H	0.032859
115	C	-0.072343
116	H	0.042485

117	H	0.037863
118	H	0.039639
119	C	-0.084076
120	H	0.032185
121	H	0.039904
122	H	0.024062
123	C	-0.085811
124	H	0.042484
125	H	0.019981
126	H	0.017027
127	O	-0.302452
128	S	0.509105
129	O	-0.314609
130	O	-0.306012
131	C	0.291735
132	F	-0.082357
133	F	-0.08237
134	F	-0.076357
135	Cl	0.019483
136	Cl	0.046223
	Tot	0.999778

Mo10 4r-1α

1	Mo	0.638862
2	N	-0.160479
3	C	-0.008007
4	N	0.066683
5	N	0.006301
6	C	0.103762
7	C	0.035528
8	C	-0.033308
9	C	-0.016252
10	H	0.052378
11	C	-0.010346
12	C	-0.014657
13	H	0.049778
14	C	-0.039412
15	C	0.043357
16	C	-0.033656
17	C	-0.019781
18	H	0.049649
19	C	-0.014735
20	C	-0.014704
21	H	0.052345
22	C	-0.027999
23	C	0.034032

24	C	0.021369
25	C	-0.032645
26	H	0.044004
27	C	-0.011128
28	H	0.052106
29	C	-0.026595
30	H	0.05077
31	C	0.02278
32	C	-0.082391
33	H	0.039115
34	H	0.019996
35	H	0.037654
36	C	-0.077804
37	H	0.045349
38	H	0.027117
39	H	0.033027
40	C	-0.094875
41	H	0.033068
42	C	-0.017067
43	C	-0.072914
44	C	-0.010362
45	C	0.007962
46	C	-0.046884
47	C	0.009384
48	C	-0.058107
49	C	-0.076886
50	C	0.212823
51	C	0.216168
52	O	-0.120088
53	O	-0.256243
54	O	-0.120554
55	O	-0.256188
56	C	0.041898
57	C	-0.078206
58	C	0.048608
59	C	-0.079373
60	H	0.028167
61	H	0.013776
62	H	0.033237
63	H	0.036104
64	H	0.034213
65	H	0.042937
66	H	0.045429
67	H	0.040109
68	H	0.032191
69	H	0.034798

70	H	0.043196
71	H	0.026064
72	H	0.040075
73	H	0.038351
74	H	0.028608
75	H	0.041205
76	H	0.034113
77	C	-0.03519
78	C	0.014996
79	C	0.244222
80	C	0.215637
81	C	-0.02299
82	C	-0.047825
83	C	-0.085404
84	O	-0.246409
85	O	-0.106078
86	O	-0.210734
87	O	-0.075139
88	C	0.049366
89	C	-0.080503
90	C	0.056017
91	C	-0.074696
92	H	0.081258
93	H	0.054812
94	H	0.04704
95	H	0.094582
96	H	0.043348
97	H	0.049941
98	H	0.049217
99	H	0.029744
100	H	0.036964
101	H	0.046066
102	H	0.041935
103	H	0.035337
104	H	0.046005
105	H	0.032713
106	H	0.045623
107	H	0.033144
108	O	-0.309875
109	S	0.504915
110	O	-0.313266
111	O	-0.313502
112	C	0.289834
113	F	-0.089671
114	F	-0.083168
115	F	-0.087957

116	H	0.053944
117	H	0.03459
118	H	0.046944
119	H	0.049097
120	H	0.052765
121	H	0.034498
122	N	-0.110308
123	C	-0.01449
124	C	-0.021342
125	C	-0.036458
126	C	-0.020504
127	C	-0.026131
128	C	-0.013264
129	H	0.04782
130	H	0.030094
131	H	0.053072
132	H	0.049139
133	H	0.053305
	Tot	0.999881

Mo13 4r-1α

1	Mo	0.657902
2	N	-0.187222
3	C	0.03079
4	N	-0.004527
5	C	0.013032
6	H	0.040822
7	N	-0.016067
8	C	0.012718
9	H	0.044776
10	C	0.024687
11	C	0.0003
12	C	-0.047365
13	H	0.03814
14	C	0.013992
15	C	-0.041432
16	H	0.041329
17	C	0.00084
18	C	0.023283
19	C	0.005171
20	C	-0.04555
21	H	0.041227
22	C	0.01244
23	C	-0.04798
24	H	0.035074
25	C	-0.000725

26	C	-0.087525
27	H	0.037064
28	H	0.032429
29	H	0.017119
30	C	-0.076225
31	H	0.033866
32	H	0.042274
33	H	0.03539
34	C	-0.084828
35	H	0.038549
36	H	0.030524
37	H	0.022542
38	C	0.05132
39	C	-0.020713
40	C	-0.019938
41	H	0.047962
42	C	-0.008862
43	H	0.05598
44	C	-0.006423
45	H	0.057648
46	C	-0.016928
47	C	-0.089471
48	H	0.026372
49	C	-0.016701
50	C	-0.076617
51	C	-0.019811
52	C	0.008136
53	C	-0.048281
54	C	0.008551
55	C	-0.061887
56	C	-0.077707
57	C	0.21299
58	C	0.216583
59	O	-0.116261
60	O	-0.258686
61	O	-0.119987
62	O	-0.256935
63	C	0.044124
64	C	-0.079376
65	C	0.048502
66	C	-0.079116
67	H	0.027329
68	H	0.007155
69	H	0.031291
70	H	0.03464
71	H	0.034135

72	H	0.041774
73	H	0.044844
74	H	0.0391
75	H	0.033673
76	H	0.035492
77	H	0.042883
78	H	0.028002
79	H	0.041034
80	H	0.038899
81	H	0.025895
82	H	0.040417
83	H	0.030389
84	C	-0.035411
85	C	0.01674
86	C	0.234106
87	C	0.219816
88	C	-0.026532
89	C	-0.048692
90	C	-0.100384
91	O	-0.243175
92	O	-0.101313
93	O	-0.219943
94	O	-0.087329
95	C	0.051566
96	C	-0.080361
97	C	0.048035
98	C	-0.075475
99	H	0.073129
100	H	0.052336
101	H	0.046937
102	H	0.0889
103	H	0.038767
104	H	0.046989
105	H	0.041023
106	H	0.028762
107	H	0.03837
108	H	0.042694
109	H	0.040082
110	H	0.03808
111	H	0.04938
112	H	0.026676
113	H	0.045136
114	H	0.035523
115	C	-0.076178
116	H	0.033275
117	H	0.038854

118	H	0.040049
119	C	-0.083825
120	H	0.035103
121	H	0.037871
122	H	0.023686
123	C	-0.079464
124	H	0.041541
125	H	0.031189
126	H	0.021613
127	O	-0.262152
128	C	0.059974
129	C	0.071443
130	C	0.075113
131	C	0.077831
132	C	0.076636
133	C	0.07605
134	C	0.302914
135	F	-0.080459
136	F	-0.100952
137	F	-0.094772
138	H	0.045269
139	F	-0.069887
140	F	-0.080741
141	F	-0.086465
142	F	-0.088081
143	F	-0.071211
144	C	-0.043598
145	H	0.039189
146	H	0.052425
147	H	0.036416
148	H	0.048472
	Tot	0.999816

Mo14 4r-1α

1	Mo	0.651989
2	N	-0.171199
3	C	0.030354
4	N	-0.004854
5	C	0.012393
6	H	0.048055
7	N	-0.016164
8	C	0.012265
9	H	0.043953
10	C	0.026928
11	C	0.000269
12	C	-0.047814

13	H	0.038225
14	C	0.013209
15	C	-0.042437
16	H	0.040945
17	C	0.000799
18	C	0.0259
19	C	0.00685
20	C	-0.042149
21	H	0.038823
22	C	0.017463
23	C	-0.042314
24	H	0.038058
25	C	0.00326
26	C	-0.086165
27	H	0.038085
28	H	0.031754
29	H	0.017649
30	C	-0.074624
31	H	0.036219
32	H	0.042598
33	H	0.033764
34	C	-0.082427
35	H	0.040696
36	H	0.029565
37	H	0.025894
38	C	0.03832
39	C	0.020808
40	C	-0.032667
41	H	0.042575
42	C	-0.016446
43	H	0.04882
44	C	-0.029225
45	H	0.049126
46	C	0.022194
47	C	-0.090662
48	H	0.025159
49	C	-0.0143
50	C	-0.079083
51	C	-0.020966
52	C	0.012987
53	C	-0.047163
54	C	0.008615
55	C	-0.059594
56	C	-0.077336
57	C	0.21474
58	C	0.216579

59	O	-0.117204
60	O	-0.254669
61	O	-0.12055
62	O	-0.257298
63	C	0.042795
64	C	-0.078748
65	C	0.048251
66	C	-0.079555
67	H	0.025161
68	H	0.015255
69	H	0.033156
70	H	0.035916
71	H	0.036835
72	H	0.041531
73	H	0.045195
74	H	0.039197
75	H	0.032854
76	H	0.034716
77	H	0.042832
78	H	0.027914
79	H	0.039857
80	H	0.037467
81	H	0.028381
82	H	0.040745
83	H	0.029172
84	C	-0.033372
85	C	0.016984
86	C	0.233979
87	C	0.219559
88	C	-0.026089
89	C	-0.049206
90	C	-0.099437
91	O	-0.243661
92	O	-0.102417
93	O	-0.218894
94	O	-0.088365
95	C	0.051094
96	C	-0.079532
97	C	0.04841
98	C	-0.076446
99	H	0.0713
100	H	0.052008
101	H	0.04572
102	H	0.089501
103	H	0.042078
104	H	0.04581

105	H	0.041499
106	H	0.027789
107	H	0.035937
108	H	0.042429
109	H	0.03997
110	H	0.038187
111	H	0.048827
112	H	0.029534
113	H	0.04534
114	H	0.035974
115	C	-0.075839
116	H	0.032587
117	H	0.039124
118	H	0.039895
119	C	-0.083424
120	H	0.034964
121	H	0.038569
122	H	0.022784
123	C	-0.078721
124	H	0.043398
125	H	0.028575
126	H	0.0237
127	O	-0.267015
128	C	0.059032
129	C	0.069558
130	C	0.073071
131	C	0.076682
132	C	0.075651
133	C	0.074603
134	F	-0.068879
135	F	-0.090487
136	F	-0.087547
137	F	-0.068429
138	F	-0.082833
139	C	-0.043863
140	H	0.040389
141	H	0.038865
142	H	0.035817
143	H	0.052049
144	C	-0.083939
145	H	0.020837
146	H	0.035377
147	H	0.035381
148	C	-0.081434
149	H	0.041293
150	H	0.021286

151	H	0.037081
	Tot	1.000171

Mo15 4r-1α

1	Mo	0.657901
2	N	-0.192716
3	C	0.032073
4	N	0.000598
5	C	0.013464
6	H	0.048684
7	N	-0.023746
8	C	0.010926
9	H	0.039803
10	C	0.024487
11	C	0.003985
12	C	-0.04186
13	H	0.041498
14	C	0.0185
15	C	-0.042105
16	H	0.041349
17	C	0.00382
18	C	0.02515
19	C	0.002702
20	C	-0.046067
21	H	0.041205
22	C	0.011894
23	C	-0.048769
24	H	0.038989
25	C	-0.000097
26	C	-0.085024
27	H	0.021956
28	H	0.040105
29	H	0.023017
30	C	-0.077516
31	H	0.034287
32	H	0.044744
33	H	0.037119
34	C	-0.084275
35	H	0.042895
36	H	0.029745
37	H	0.025143
38	C	0.018251
39	C	0.095806
40	C	0.083181
41	C	0.104074
42	C	0.089975

43	C	0.097978
44	C	-0.090122
45	H	0.022665
46	C	-0.018296
47	C	-0.079261
48	C	-0.02875
49	C	0.009292
50	C	-0.050094
51	C	0.008571
52	C	-0.056309
53	C	-0.078209
54	C	0.22031
55	C	0.212855
56	O	-0.111897
57	O	-0.259906
58	O	-0.121508
59	O	-0.257598
60	C	0.048868
61	C	-0.080784
62	C	0.047957
63	C	-0.079825
64	H	0.02921
65	H	0.012154
66	H	0.028847
67	H	0.034832
68	H	0.034754
69	H	0.041143
70	H	0.043794
71	H	0.038796
72	H	0.032912
73	H	0.034924
74	H	0.042398
75	H	0.04053
76	H	0.0452
77	H	0.035037
78	H	0.023606
79	H	0.044061
80	H	0.030608
81	C	-0.034939
82	C	0.015971
83	C	0.233495
84	C	0.214805
85	C	-0.025006
86	C	-0.047186
87	C	-0.090204
88	O	-0.247607

89	O	-0.107973
90	O	-0.22692
91	O	-0.085503
92	C	0.049591
93	C	-0.080089
94	C	0.050859
95	C	-0.075728
96	H	0.069742
97	H	0.052879
98	H	0.046967
99	H	0.092636
100	H	0.043743
101	H	0.046165
102	H	0.049359
103	H	0.026592
104	H	0.038145
105	H	0.042032
106	H	0.044457
107	H	0.035884
108	H	0.046175
109	H	0.033252
110	H	0.044779
111	H	0.035016
112	C	-0.072432
113	H	0.041528
114	H	0.039475
115	H	0.039712
116	C	-0.079269
117	H	0.034382
118	H	0.042852
119	H	0.029072
120	C	-0.078435
121	H	0.043813
122	H	0.032282
123	H	0.021167
124	O	-0.301349
125	C	-0.044246
126	H	0.041318
127	H	0.039232
128	H	0.033624
129	H	0.052454
130	F	-0.071088
131	F	-0.069646
132	F	-0.064897
133	F	-0.077135
134	F	-0.061059

135	C	0.060117
136	C	-0.103485
137	H	0.035738
138	H	0.02964
139	H	0.025768
140	C	0.292003
141	F	-0.092864
142	F	-0.106802
143	F	-0.080769
144	C	0.290407
145	F	-0.089721
146	F	-0.080754
147	F	-0.096054
	Tot	0.999859

Mo11 4r-1α

1	Mo	0.657902
2	N	-0.186589
3	C	-0.014623
4	N	0.018597
5	C	0.002808
6	H	0.068238
7	N	0.021643
8	C	0.004864
9	H	0.068789
10	C	0.034148
11	C	0.012673
12	C	-0.035942
13	H	0.043031
14	C	-0.02096
15	H	0.049511
16	C	-0.028976
17	H	0.048472
18	C	0.017052
19	C	-0.091237
20	H	0.027689
21	C	-0.012464
22	C	-0.066651
23	C	-0.025441
24	C	0.014062
25	C	-0.046643
26	C	0.008251
27	C	-0.05678
28	C	-0.077938
29	C	0.215269
30	C	0.216593

31	O	-0.118193
32	O	-0.253849
33	O	-0.120412
34	O	-0.258597
35	C	0.04249
36	C	-0.079638
37	C	0.04821
38	C	-0.079165
39	H	0.027591
40	H	0.017724
41	H	0.035312
42	H	0.035807
43	H	0.037715
44	H	0.040085
45	H	0.045247
46	H	0.039155
47	H	0.03316
48	H	0.035142
49	H	0.043192
50	H	0.026484
51	H	0.039544
52	H	0.036476
53	H	0.026982
54	H	0.040492
55	H	0.028606
56	C	-0.033678
57	C	0.015483
58	C	0.225425
59	C	0.220702
60	C	-0.022513
61	C	-0.04416
62	C	-0.094461
63	O	-0.241274
64	O	-0.103272
65	O	-0.227431
66	O	-0.092012
67	C	0.049578
68	C	-0.075807
69	C	0.046827
70	C	-0.076002
71	H	0.076428
72	H	0.05353
73	H	0.047048
74	H	0.090292
75	H	0.04181
76	H	0.047634

77	H	0.036262
78	H	0.031429
79	H	0.036457
80	H	0.041579
81	H	0.038982
82	H	0.041482
83	H	0.041771
84	H	0.033631
85	H	0.046221
86	H	0.034475
87	O	-0.251343
88	C	0.046495
89	H	0.026763
90	C	-0.081462
91	H	0.0413
92	H	0.033263
93	H	0.032699
94	C	-0.08212
95	H	0.040927
96	H	0.028442
97	H	0.032355
98	C	0.045709
99	H	0.0254
100	C	-0.086562
101	H	0.026115
102	H	0.034555
103	H	0.029494
104	C	-0.085446
105	H	0.038538
106	H	0.022329
107	H	0.031766
108	C	-0.084311
109	H	0.042065
110	H	0.024293
111	H	0.022351
112	C	-0.08127
113	H	0.023064
114	H	0.033471
115	H	0.038547
116	C	0.08069
117	C	-0.010384
118	C	-0.009317
119	C	-0.032213
120	C	-0.032789
121	C	-0.042552
122	H	0.042729

123	H	0.041508
124	H	0.044394
125	C	-0.010294
126	C	0.012266
127	C	0.006109
128	C	-0.047134
129	C	-0.046907
130	C	0.011352
131	H	0.035523
132	H	0.034602
133	C	-0.009998
134	C	0.006109
135	C	0.011473
136	C	-0.049955
137	C	-0.05117
138	C	0.00661
139	H	0.03286
140	H	0.028008
141	C	-0.081824
142	H	0.039938
143	H	0.024091
144	H	0.032589
145	C	-0.086905
146	H	0.017684
147	H	0.033394
148	H	0.034551
149	C	-0.07536
150	H	0.035276
151	H	0.038824
152	H	0.036768
153	C	-0.088577
154	H	0.024028
155	H	0.031737
156	H	0.019725
157	C	-0.086276
158	H	0.033309
159	H	0.031945
160	H	0.01499
161	C	-0.07554
162	H	0.031411
163	H	0.040352
164	H	0.033761
	Tot	1.000181

Mo4 4r-1α

1	Mo	0.657634

2	N	-0.186932
3	C	-0.008673
4	N	0.022892
5	C	0.00908
6	H	0.073801
7	N	0.023894
8	C	0.007336
9	H	0.073761
10	C	0.025075
11	C	0.015114
12	C	-0.035301
13	H	0.04344
14	C	0.022049
15	C	-0.043807
16	H	0.038513
17	C	0.0027
18	C	0.026311
19	C	0.013734
20	C	-0.042737
21	H	0.038419
22	C	0.021239
23	C	-0.040593
24	H	0.039093
25	C	0.00974
26	C	-0.086428
27	H	0.028865
28	H	0.037271
29	H	0.031432
30	C	-0.072216
31	H	0.039489
32	H	0.041592
33	H	0.036265
34	C	-0.084208
35	H	0.03768
36	H	0.03575
37	H	0.023044
38	C	0.034838
39	C	0.01589
40	C	-0.037561
41	H	0.040733
42	C	-0.02192
43	H	0.047966
44	C	-0.033981
45	H	0.046595
46	C	0.01921
47	C	-0.089174

48	H	0.027979
49	H	0.017947
50	H	0.032239
51	C	-0.086308
52	H	0.033657
53	H	0.021079
54	H	0.037617
55	C	-0.085848
56	H	0.035379
57	C	-0.011317
58	C	-0.070444
59	C	-0.034097
60	C	0.018132
61	C	-0.044295
62	C	0.008158
63	C	-0.058825
64	C	-0.074684
65	C	0.215634
66	C	0.216779
67	O	-0.115948
68	O	-0.252746
69	O	-0.119971
70	O	-0.258677
71	C	0.044092
72	C	-0.080307
73	C	0.048551
74	C	-0.079465
75	H	0.026953
76	H	0.017463
77	H	0.037621
78	H	0.037203
79	H	0.038915
80	H	0.043335
81	H	0.045727
82	H	0.039239
83	H	0.03329
84	H	0.034809
85	H	0.043072
86	H	0.029917
87	H	0.040223
88	H	0.03629
89	H	0.026162
90	H	0.040142
91	H	0.031376
92	C	-0.032474
93	C	0.012506

94	C	0.230207
95	C	0.217407
96	C	-0.025521
97	C	-0.048423
98	C	-0.09412
99	O	-0.242036
100	O	-0.111971
101	O	-0.22056
102	O	-0.095757
103	C	0.040889
104	C	-0.080044
105	C	0.047627
106	C	-0.077409
107	H	0.077893
108	H	0.049055
109	H	0.047868
110	H	0.090995
111	H	0.041905
112	H	0.045666
113	H	0.04607
114	H	0.028627
115	H	0.037918
116	H	0.040447
117	H	0.040842
118	H	0.026406
119	H	0.036495
120	H	0.036029
121	H	0.042377
122	H	0.033244
123	C	-0.071609
124	H	0.039739
125	H	0.041674
126	H	0.039221
127	C	-0.083077
128	H	0.036282
129	H	0.03824
130	H	0.026026
131	C	-0.082988
132	H	0.041346
133	H	0.030083
134	H	0.020174
135	O	-0.250479
136	C	0.065026
137	C	-0.006305
138	C	0.001565
139	C	-0.050926

140	C	-0.042588
141	C	0.002926
142	H	0.033375
143	H	0.037604
144	C	-0.090079
145	H	0.031492
146	H	0.024293
147	H	0.01156
148	C	-0.082902
149	H	0.028934
150	H	0.0339
151	H	0.020639
152	C	-0.075829
153	H	0.034765
154	H	0.036842
155	H	0.035925
	Tot	0.999975

Mo5 4r-1α

1	Mo	0.650286
2	N	-0.200699
3	C	-0.010536
4	N	0.027493
5	C	0.009937
6	H	0.073493
7	N	0.024146
8	C	0.009764
9	H	0.073887
10	C	0.025838
11	C	0.004511
12	C	-0.04575
13	H	0.040234
14	C	0.015087
15	C	-0.04185
16	H	0.043097
17	C	0.003693
18	C	0.022455
19	C	0.005988
20	C	-0.046911
21	H	0.038357
22	C	0.016022
23	C	-0.04587
24	H	0.038196
25	C	0.002948
26	C	-0.088112
27	H	0.027417

28	H	0.038148
29	H	0.020315
30	C	-0.074515
31	H	0.036002
32	H	0.044584
33	H	0.035035
34	C	-0.08752
35	H	0.036813
36	H	0.037248
37	H	0.017397
38	C	0.030313
39	C	0.037955
40	C	-0.029526
41	H	0.052547
42	C	-0.005435
43	H	0.058593
44	C	-0.02536
45	H	0.058309
46	C	0.037714
47	C	-0.077426
48	H	0.031843
49	C	-0.018793
50	C	-0.074052
51	C	-0.021286
52	C	0.016844
53	C	-0.046356
54	C	0.007955
55	C	-0.060331
56	C	-0.07956
57	C	0.212253
58	C	0.217141
59	O	-0.118255
60	O	-0.256484
61	O	-0.119975
62	O	-0.256357
63	C	0.043516
64	C	-0.080134
65	C	0.048646
66	C	-0.07951
67	H	0.027398
68	H	0.009122
69	H	0.033091
70	H	0.037463
71	H	0.032282
72	H	0.043023
73	H	0.045645

74	H	0.039711
75	H	0.032722
76	H	0.035043
77	H	0.043247
78	H	0.028333
79	H	0.04122
80	H	0.038636
81	H	0.022609
82	H	0.040489
83	H	0.029719
84	C	-0.033187
85	C	0.015345
86	C	0.224423
87	C	0.217978
88	C	-0.025721
89	C	-0.04805
90	C	-0.099645
91	O	-0.252792
92	O	-0.103832
93	O	-0.234785
94	O	-0.095677
95	C	0.050833
96	C	-0.079285
97	C	0.046368
98	C	-0.0788
99	H	0.079687
100	H	0.050642
101	H	0.047024
102	H	0.089113
103	H	0.040627
104	H	0.045575
105	H	0.04124
106	H	0.028542
107	H	0.03084
108	H	0.041024
109	H	0.037837
110	H	0.037612
111	H	0.048167
112	H	0.033054
113	H	0.044603
114	H	0.035955
115	C	-0.076131
116	H	0.033181
117	H	0.038701
118	H	0.042881
119	C	-0.08348

120	H	0.036004
121	H	0.039186
122	H	0.027282
123	C	-0.08571
124	H	0.042565
125	H	0.024988
126	H	0.018636
127	O	-0.254207
128	C	0.057434
129	C	0.07039
130	C	0.077759
131	C	0.076296
132	C	0.077866
133	C	0.078236
134	Cl	0.014309
135	Cl	0.034931
136	F	-0.060635
137	F	-0.087817
138	F	-0.08437
139	F	-0.083666
140	F	-0.070529
	Tot	0.999986

Mo19 4r-1α

1	Mo	0.60974
2	N	-0.151
3	C	0.021626
4	N	0.03827
5	C	0.076891
6	C	-0.048108
7	H	0.033433
8	C	0.029004
9	C	0.010061
10	C	-0.032564
11	H	0.043632
12	C	-0.022963
13	C	-0.033994
14	H	0.042602
15	C	0.006762
16	C	0.043895
17	C	0.023012
18	C	-0.031517
19	H	0.043732
20	C	-0.013904
21	H	0.05055
22	C	-0.026762

23	H	0.048314
24	C	0.022839
25	C	-0.012499
26	C	-0.0905
27	H	0.02354
28	C	-0.011516
29	C	-0.079681
30	C	-0.017188
31	C	0.018425
32	C	-0.047339
33	C	0.007996
34	C	-0.060232
35	C	-0.072753
36	C	0.216974
37	C	0.215489
38	O	-0.116427
39	O	-0.252898
40	O	-0.121098
41	O	-0.258058
42	C	0.043601
43	C	-0.078091
44	C	0.047858
45	C	-0.079827
46	H	0.023896
47	H	0.014005
48	H	0.036223
49	H	0.035031
50	H	0.04112
51	H	0.041045
52	H	0.044826
53	H	0.038532
54	H	0.033137
55	H	0.034344
56	H	0.042666
57	H	0.0291
58	H	0.041155
59	H	0.037946
60	H	0.028577
61	H	0.041657
62	H	0.037605
63	C	-0.036679
64	C	0.01432
65	C	0.241174
66	C	0.212172
67	C	-0.023167
68	C	-0.048581

69	C	-0.088735
70	O	-0.239729
71	O	-0.109085
72	O	-0.213809
73	O	-0.07563
74	C	0.048012
75	C	-0.078747
76	C	0.054893
77	C	-0.073753
78	H	0.069579
79	H	0.052746
80	H	0.048532
81	H	0.09288
82	H	0.042216
83	H	0.047966
84	H	0.044142
85	H	0.037298
86	H	0.042402
87	H	0.044379
88	H	0.041917
89	H	0.035788
90	H	0.042119
91	H	0.036634
92	H	0.04504
93	H	0.035413
94	C	-0.011034
95	H	0.011274
96	C	-0.011736
97	H	0.013316
98	C	-0.078431
99	H	0.029111
100	H	0.027458
101	H	0.031907
102	C	-0.078357
103	H	0.028447
104	H	0.030413
105	H	0.031617
106	C	-0.010785
107	H	0.015344
108	C	-0.079018
109	H	0.032855
110	H	0.024116
111	H	0.029479
112	C	-0.077863
113	H	0.031242
114	H	0.02176

115	H	0.033049
116	H	0.011025
117	Br	-0.215579
118	C	0.019209
119	H	0.049323
120	H	0.042478
121	C	-0.089031
122	H	0.006959
123	H	0.023827
124	H	0.029867
125	C	-0.079464
126	H	0.032436
127	H	0.029588
128	H	0.026243
129	C	-0.084959
130	H	0.028103
131	H	0.009984
132	H	0.029755
133	C	-0.078018
134	H	0.032697
135	H	0.028523
136	H	0.030783
137	C	-0.07983
138	H	0.032716
139	H	0.03587
140	H	0.044641
141	C	-0.079354
142	H	0.035673
143	H	0.033396
144	H	0.04375
145	C	-0.086539
146	H	0.036376
147	H	0.024674
148	H	0.022779
149	C	-0.079769
150	H	0.02373
151	H	0.036893
152	H	0.025148
	Tot	0.999965

Mo16 4r-1α

1	Mo	0.665545
2	N	-0.170673
3	C	0.020974
4	N	0.036474
5	C	0.077905

6	C	-0.048436
7	H	0.042395
8	C	0.032491
9	C	0.010467
10	C	-0.035282
11	H	0.043389
12	C	-0.025953
13	C	-0.031367
14	H	0.045037
15	C	0.007997
16	C	0.042056
17	C	0.019538
18	C	-0.032386
19	H	0.043496
20	C	-0.017275
21	H	0.049974
22	C	-0.02986
23	H	0.046874
24	C	0.018977
25	C	-0.014008
26	C	-0.084561
27	H	0.029575
28	C	-0.01063
29	C	-0.073151
30	C	-0.021937
31	C	0.02524
32	C	-0.045738
33	C	0.006762
34	C	-0.061033
35	C	-0.074376
36	C	0.217393
37	C	0.215242
38	O	-0.114957
39	O	-0.253978
40	O	-0.120041
41	O	-0.262675
42	C	0.044388
43	C	-0.079028
44	C	0.048118
45	C	-0.079477
46	H	0.023017
47	H	0.016009
48	H	0.039316
49	H	0.036344
50	H	0.043374
51	H	0.042395

52	H	0.045107
53	H	0.038008
54	H	0.03428
55	H	0.034311
56	H	0.042674
57	H	0.029523
58	H	0.041867
59	H	0.037472
60	H	0.027764
61	H	0.041006
62	H	0.032102
63	C	-0.030657
64	C	0.017469
65	C	0.230472
66	C	0.220786
67	C	-0.029135
68	C	-0.048013
69	C	-0.103278
70	O	-0.240486
71	O	-0.100207
72	O	-0.218985
73	O	-0.088981
74	C	0.050147
75	C	-0.08054
76	C	0.050418
77	C	-0.076414
78	H	0.076696
79	H	0.048975
80	H	0.045032
81	H	0.087967
82	H	0.041924
83	H	0.047122
84	H	0.0445
85	H	0.032123
86	H	0.035436
87	H	0.041824
88	H	0.040268
89	H	0.036488
90	H	0.048287
91	H	0.026748
92	H	0.044176
93	H	0.035179
94	C	-0.012948
95	H	0.014831
96	C	-0.011763
97	H	0.015655

98	C	-0.077648
99	H	0.030828
100	H	0.028742
101	H	0.029772
102	C	0.019773
103	H	0.049501
104	H	0.034419
105	C	-0.091623
106	H	0.012805
107	H	0.02738
108	H	0.021229
109	C	-0.077094
110	H	0.033146
111	H	0.03316
112	H	0.0296
113	C	-0.083437
114	H	0.029822
115	H	0.019265
116	H	0.029057
117	C	-0.076109
118	H	0.034597
119	H	0.027319
120	H	0.033115
121	C	-0.081904
122	H	0.028819
123	H	0.036039
124	H	0.043058
125	C	-0.080738
126	H	0.036274
127	H	0.035026
128	H	0.045321
129	C	-0.082597
130	H	0.037275
131	H	0.023792
132	H	0.02623
133	C	-0.078491
134	H	0.027002
135	H	0.038297
136	H	0.029181
137	C	-0.078112
138	H	0.028156
139	H	0.030611
140	H	0.031266
141	O	-0.264134
142	C	0.060595
143	C	0.064724

144	C	0.074559
145	C	0.075114
146	C	0.074821
147	C	0.072684
148	F	-0.069423
149	F	-0.086445
150	F	-0.087544
151	F	-0.089322
152	F	-0.064705
153	H	0.011162
154	C	-0.010521
155	H	0.014074
156	C	-0.077116
157	H	0.031632
158	H	0.025268
159	H	0.034108
160	C	-0.08162
161	H	0.030118
162	H	0.021399
163	H	0.029532
	Tot	1.00026

Mo17 4r-1α

1	Mo	0.658792
2	N	-0.164783
3	C	0.02252
4	N	0.036301
5	C	0.07916
6	C	-0.049256
7	H	0.042317
8	C	0.032307
9	C	0.01365
10	C	-0.033144
11	H	0.044911
12	C	-0.027337
13	C	-0.033812
14	H	0.044099
15	C	0.007633
16	C	0.046555
17	C	0.020768
18	C	-0.029476
19	H	0.043157
20	C	-0.014566
21	H	0.052149
22	C	-0.021513
23	H	0.053757

24	C	-0.026947
25	C	0.028819
26	C	-0.091508
27	H	0.021045
28	C	-0.014463
29	C	-0.073738
30	C	-0.023151
31	C	0.021371
32	C	-0.046581
33	C	0.007534
34	C	-0.062074
35	C	-0.076029
36	C	0.214306
37	C	0.215942
38	O	-0.117121
39	O	-0.256664
40	O	-0.120632
41	O	-0.25945
42	C	0.043574
43	C	-0.077209
44	C	0.048322
45	C	-0.079232
46	H	0.025155
47	H	0.012526
48	H	0.037381
49	H	0.035719
50	H	0.040557
51	H	0.041913
52	H	0.045388
53	H	0.038914
54	H	0.033415
55	H	0.034569
56	H	0.04339
57	H	0.029256
58	H	0.04117
59	H	0.037675
60	H	0.030722
61	H	0.042603
62	H	0.030531
63	C	-0.037101
64	C	0.014279
65	C	0.237162
66	C	0.214537
67	C	-0.023759
68	C	-0.049463
69	C	-0.090744

70	O	-0.2415
71	O	-0.111121
72	O	-0.218961
73	O	-0.082722
74	C	0.044724
75	C	-0.078907
76	C	0.053022
77	C	-0.074308
78	H	0.075527
79	H	0.050925
80	H	0.048362
81	H	0.092358
82	H	0.042687
83	H	0.046835
84	H	0.047424
85	H	0.032689
86	H	0.042864
87	H	0.042656
88	H	0.040786
89	H	0.02179
90	H	0.04194
91	H	0.038277
92	H	0.045794
93	H	0.036318
94	C	-0.0118
95	H	0.013369
96	C	-0.013685
97	H	0.014833
98	C	-0.083965
99	H	0.029186
100	H	0.01707
101	H	0.031408
102	C	-0.077568
103	H	0.031034
104	H	0.034133
105	H	0.02944
106	C	0.020816
107	H	0.046668
108	H	0.034059
109	C	-0.091123
110	H	0.013969
111	H	0.025313
112	H	0.026861
113	C	-0.077266
114	H	0.033579
115	H	0.033058

116	H	0.028744
117	C	-0.083607
118	H	0.030532
119	H	0.018505
120	H	0.030155
121	C	-0.076223
122	H	0.034775
123	H	0.026862
124	H	0.034015
125	C	-0.079503
126	H	0.029935
127	H	0.037537
128	H	0.043623
129	C	-0.079661
130	H	0.036302
131	H	0.036107
132	H	0.046124
133	C	-0.085717
134	H	0.036447
135	H	0.021404
136	H	0.024948
137	C	-0.078417
138	H	0.026532
139	H	0.038488
140	H	0.028542
141	H	0.039983
142	C	-0.085991
143	H	0.01268
144	H	0.02934
145	H	0.030367
146	O	-0.271731
147	C	0.052258
148	C	0.064421
149	C	0.072007
150	C	0.073903
151	C	0.080285
152	C	0.074062
153	F	-0.060781
154	F	-0.083997
155	F	-0.086149
156	F	-0.088718
157	F	-0.061354
	Tot	0.999982

Mo21 4r-1α

1	Mo	0.660493

2	N	-0.141222
3	C	0.021754
4	N	0.040191
5	C	0.078459
6	C	-0.047769
7	H	0.034858
8	C	0.028173
9	C	0.009822
10	C	-0.035934
11	H	0.042725
12	C	-0.022311
13	C	-0.027523
14	H	0.046739
15	C	0.008796
16	C	0.04415
17	C	0.023808
18	C	-0.030942
19	H	0.044556
20	C	-0.010682
21	H	0.051869
22	C	-0.026047
23	H	0.049824
24	C	0.025165
25	C	-0.012009
26	C	-0.087335
27	H	0.024912
28	C	-0.011335
29	C	-0.073116
30	C	-0.011672
31	C	0.027307
32	C	-0.045452
33	C	0.008158
34	C	-0.060221
35	C	-0.072926
36	C	0.215852
37	C	0.215965
38	O	-0.116158
39	O	-0.250097
40	O	-0.120748
41	O	-0.256932
42	C	0.043825
43	C	-0.077953
44	C	0.048698
45	C	-0.079682
46	H	0.024564
47	H	0.013027

48	H	0.037629
49	H	0.037848
50	H	0.040948
51	H	0.043142
52	H	0.04574
53	H	0.039756
54	H	0.032698
55	H	0.0343
56	H	0.04328
57	H	0.029246
58	H	0.041579
59	H	0.038209
60	H	0.028181
61	H	0.042196
62	H	0.037825
63	C	-0.034241
64	C	0.013412
65	C	0.242475
66	C	0.215127
67	C	-0.026184
68	C	-0.046058
69	C	-0.087545
70	O	-0.242923
71	O	-0.10806
72	O	-0.212956
73	O	-0.075289
74	C	0.046036
75	C	-0.082514
76	C	0.054316
77	C	-0.073205
78	H	0.080288
79	H	0.05247
80	H	0.046144
81	H	0.093469
82	H	0.042563
83	H	0.050666
84	H	0.043979
85	H	0.036848
86	H	0.042238
87	H	0.04515
88	H	0.042587
89	H	0.02635
90	H	0.044996
91	H	0.031277
92	H	0.044071
93	H	0.031378

94	C	-0.011762
95	H	0.01066
96	C	-0.013675
97	H	0.011501
98	C	-0.077691
99	H	0.030119
100	H	0.028301
101	H	0.03276
102	C	-0.077865
103	H	0.029118
104	H	0.031164
105	H	0.032653
106	C	-0.01218
107	H	0.016411
108	C	-0.07888
109	H	0.031909
110	H	0.025897
111	H	0.030598
112	C	-0.077951
113	H	0.033215
114	H	0.018843
115	H	0.031495
116	H	0.011996
117	C	0.019442
118	H	0.050213
119	H	0.042835
120	C	-0.091606
121	H	0.011224
122	H	0.021952
123	H	0.024536
124	C	-0.078729
125	H	0.032754
126	H	0.030161
127	H	0.026609
128	C	-0.082967
129	H	0.026903
130	H	0.011406
131	H	0.033581
132	C	-0.077537
133	H	0.033963
134	H	0.027847
135	H	0.030856
136	C	-0.078774
137	H	0.033898
138	H	0.037098
139	H	0.045185

140	C	-0.07835
141	H	0.036791
142	H	0.033833
143	H	0.044308
144	C	-0.086219
145	H	0.037346
146	H	0.024443
147	H	0.023764
148	C	-0.079099
149	H	0.023862
150	H	0.037663
151	H	0.026284
152	O	-0.307626
153	S	0.506604
154	O	-0.307655
155	O	-0.308047
156	C	0.29229
157	F	-0.086481
158	F	-0.083213
159	F	-0.062967
	Tot	1.000075

Mo6 4r-1α

1	Mo	0.636319
2	N	-0.157899
3	C	0.030318
4	N	-0.005363
5	C	0.014934
6	H	0.044413
7	N	-0.021316
8	C	0.013292
9	H	0.049929
10	C	0.018346
11	C	0.000375
12	C	-0.045806
13	H	0.038506
14	C	0.022616
15	C	-0.035805
16	H	0.042453
17	C	0.000863
18	C	0.024818
19	C	0.005165
20	C	-0.044348
21	H	0.038498
22	C	0.019962
23	C	-0.040446

24	H	0.040685
25	C	0.005164
26	C	-0.082861
27	H	0.021836
28	H	0.041028
29	H	0.02569
30	C	-0.072628
31	C	-0.081695
32	H	0.041442
33	H	0.034259
34	H	0.025781
35	C	0.043758
36	C	-0.030143
37	C	0.017264
38	C	-0.073929
39	C	-0.023052
40	H	0.044941
41	C	0.023722
42	C	-0.0759
43	C	-0.030081
44	C	-0.089676
45	H	0.030336
46	C	-0.013719
47	C	-0.078413
48	C	-0.012563
49	C	0.006336
50	C	-0.047235
51	C	0.008455
52	C	-0.06084
53	C	-0.076024
54	C	0.213778
55	C	0.217346
56	O	-0.118371
57	O	-0.248754
58	O	-0.120192
59	O	-0.255023
60	C	0.044206
61	C	0.048569
62	C	-0.079597
63	H	0.026413
64	H	0.012056
65	H	0.030597
66	H	0.035237
67	H	0.032948
68	H	0.042228
69	H	0.045366

70	H	0.040027
71	H	0.03268
72	H	0.034701
73	H	0.043188
74	H	0.036316
75	C	-0.037414
76	C	0.014987
77	C	0.247164
78	C	0.215971
79	C	-0.023485
80	C	-0.047322
81	C	-0.085412
82	O	-0.245477
83	O	-0.105165
84	O	-0.204256
85	O	-0.071899
86	C	0.048637
87	C	-0.081321
88	C	0.056843
89	C	-0.075682
90	H	0.073313
91	H	0.054725
92	H	0.047743
93	H	0.094432
94	H	0.041962
95	H	0.049258
96	H	0.050977
97	H	0.031059
98	H	0.037821
99	H	0.044285
100	H	0.042689
101	H	0.032871
102	H	0.045959
103	H	0.031998
104	H	0.045347
105	H	0.032462
106	C	-0.072789
107	H	0.042896
108	H	0.037306
109	H	0.038409
110	C	-0.083071
111	H	0.032315
112	H	0.038663
113	H	0.023305
114	C	-0.080656
115	H	0.042004

116	H	0.026313
117	H	0.020528
118	O	-0.306951
119	S	0.506727
120	O	-0.31567
121	O	-0.309861
122	C	0.290258
123	F	-0.084534
124	F	-0.08401
125	F	-0.077749
126	H	0.042004
127	H	0.037234
128	H	0.037131
129	H	0.044395
130	C	-0.076699
131	H	0.030497
132	H	0.040894
133	H	0.032114
134	H	0.042265
135	H	0.038752
136	H	0.037929
137	H	0.036045
138	H	0.039096
139	H	0.038786
140	H	0.035197
141	H	0.043373
142	H	0.038595
143	H	0.040317
144	H	0.050057
	Tot	0.999972

Mo8 4r-1α

1	Mo	0.666057
2	N	-0.148582
3	C	-0.020969
4	N	0.028291
5	C	0.011433
6	H	0.070068
7	N	0.017962
8	C	0.005285
9	H	0.069219
10	C	0.037515
11	C	-0.052645
12	C	-0.044505
13	C	-0.042034
14	C	-0.045164

15	C	-0.052418
16	C	0.037562
17	C	-0.046064
18	C	-0.041595
19	C	-0.043377
20	C	-0.039816
21	C	-0.052146
22	C	0.04148
23	C	0.020001
24	C	-0.029943
25	H	0.045265
26	C	-0.012795
27	H	0.05321
28	C	-0.019156
29	H	0.052753
30	C	-0.023719
31	C	0.027838
32	C	-0.089371
33	C	-0.088725
34	C	-0.077637
35	C	-0.088922
36	H	0.033951
37	C	-0.017207
38	C	-0.074921
39	C	-0.015092
40	C	0.024575
41	C	-0.046813
42	C	0.007583
43	C	-0.057574
44	C	-0.075192
45	C	0.21957
46	C	0.216517
47	O	-0.117695
48	O	-0.247546
49	O	-0.119724
50	O	-0.255954
51	C	0.04742
52	C	-0.083254
53	C	0.048197
54	C	-0.07942
55	H	0.026864
56	H	0.020431
57	H	0.036263
58	H	0.038379
59	H	0.040331
60	H	0.045982

61	H	0.045327
62	H	0.038843
63	H	0.034178
64	H	0.034911
65	H	0.043425
66	H	0.039998
67	H	0.04503
68	H	0.031681
69	H	0.02214
70	H	0.041929
71	H	0.03533
72	C	-0.031014
73	C	0.01238
74	C	0.233366
75	C	0.218102
76	C	-0.026243
77	C	-0.045563
78	C	-0.088137
79	O	-0.223415
80	O	-0.108482
81	O	-0.211875
82	O	-0.085763
83	C	0.04508
84	C	-0.079096
85	C	0.052158
86	C	-0.07472
87	H	0.087255
88	H	0.050645
89	H	0.049876
90	H	0.093506
91	H	0.050241
92	H	0.050258
93	H	0.040363
94	H	0.036599
95	H	0.037869
96	H	0.045271
97	H	0.041172
98	H	0.026669
99	H	0.043789
100	H	0.033409
101	H	0.045905
102	H	0.036394
103	O	-0.301786
104	H	0.007918
105	H	0.026871
106	H	0.026466

107	H	0.009461
108	H	0.02335
109	H	0.027901
110	H	0.019835
111	H	0.027977
112	H	0.01969
113	H	0.023231
114	H	0.022814
115	H	0.014906
116	H	0.019411
117	H	0.031436
118	H	0.023299
119	H	0.031981
120	H	0.022252
121	H	0.017857
122	H	0.031336
123	H	0.031991
124	H	0.032267
125	H	0.036148
126	H	0.029935
127	H	0.03518
128	H	0.03548
129	H	0.018853
130	H	0.030433
131	H	0.018352
132	H	0.030168
133	H	0.029271
134	H	0.02201
135	H	0.033172
136	S	0.513313
137	O	-0.296428
138	O	-0.29301
139	C	0.290571
140	F	-0.076001
141	F	-0.084679
142	F	-0.072201
	Tot	0.999848

Mo9 4r-1α

1	Mo	0.668814
2	N	-0.164916
3	C	-0.018234
4	N	0.022755
5	C	0.002357
6	H	0.067646
7	N	0.018981

8	C	-0.001079
9	H	0.068517
10	C	0.033736
11	C	-0.051178
12	C	-0.045989
13	C	-0.041496
14	C	-0.044647
15	C	-0.05118
16	C	0.037236
17	C	-0.049897
18	C	-0.044884
19	C	-0.04408
20	C	-0.041682
21	C	-0.049471
22	C	0.043346
23	C	0.018697
24	C	-0.028058
25	H	0.04451
26	C	-0.011243
27	H	0.053464
28	C	-0.016718
29	H	0.052536
30	C	-0.02453
31	C	0.027913
32	C	-0.089406
33	C	-0.086213
34	C	-0.077881
35	C	-0.094622
36	H	0.030294
37	C	-0.014281
38	C	-0.083503
39	C	-0.018332
40	C	0.014629
41	C	-0.047371
42	C	0.007114
43	C	-0.05756
44	C	-0.076525
45	C	0.218547
46	C	0.216702
47	O	-0.119774
48	O	-0.249066
49	O	-0.119635
50	O	-0.257288
51	C	0.046823
52	C	-0.083791
53	C	0.047777

54	C	-0.079429
55	H	0.026091
56	H	0.020547
57	H	0.034687
58	H	0.036566
59	H	0.040781
60	H	0.043809
61	H	0.044875
62	H	0.038247
63	H	0.034426
64	H	0.034969
65	H	0.043007
66	H	0.039822
67	H	0.044476
68	H	0.030438
69	H	0.022765
70	H	0.041348
71	H	0.024122
72	C	-0.033705
73	C	0.013223
74	C	0.237776
75	C	0.220581
76	C	-0.027454
77	C	-0.046213
78	C	-0.089956
79	O	-0.215736
80	O	-0.106873
81	O	-0.214772
82	O	-0.084549
83	C	0.04828
84	C	-0.079794
85	C	0.051593
86	C	-0.075781
87	H	0.078713
88	H	0.050994
89	H	0.049169
90	H	0.093234
91	H	0.047772
92	H	0.048297
93	H	0.045197
94	H	0.035158
95	H	0.036626
96	H	0.045534
97	H	0.040886
98	H	0.032444
99	H	0.045583

100	H	0.033524
101	H	0.045859
102	H	0.035815
103	O	-0.258373
104	H	0.013209
105	H	0.027633
106	H	0.026803
107	H	0.009847
108	H	0.024611
109	H	0.028655
110	H	0.01966
111	H	0.028158
112	H	0.019674
113	H	0.021649
114	H	0.021674
115	H	0.014103
116	H	0.017831
117	H	0.032356
118	H	0.022695
119	H	0.031514
120	H	0.023329
121	H	0.019182
122	H	0.031544
123	H	0.032249
124	H	0.031873
125	H	0.037034
126	H	0.026117
127	H	0.035934
128	H	0.033958
129	H	0.018829
130	H	0.027067
131	H	0.025514
132	H	0.026252
133	H	0.025391
134	H	0.014905
135	H	0.03169
136	C	0.051633
137	C	0.074716
138	C	0.059957
139	C	0.082387
140	F	-0.072501
141	C	0.076207
142	F	-0.080019
143	C	0.078228
144	F	-0.071357
145	F	-0.082714

146	F	-0.080208
	Tot	0.99974

Mo7 4r-1α

1	Mo	0.643598
2	N	-0.176886
3	C	0.028724
4	N	-0.008616
5	C	0.0135
6	H	0.042829
7	N	-0.015844
8	C	0.013189
9	H	0.049393
10	C	0.029861
11	C	0.008424
12	C	-0.041206
13	H	0.042387
14	C	0.018381
15	C	-0.042066
16	H	0.0424
17	C	0.004923
18	C	0.025449
19	C	0.005993
20	C	-0.041629
21	H	0.038954
22	C	0.01948
23	C	-0.040445
24	H	0.039783
25	C	0.00471
26	C	-0.082473
27	H	0.041202
28	H	0.027997
29	H	0.020098
30	C	-0.073662
31	H	0.037083
32	H	0.042872
33	H	0.035285
34	C	-0.082094
35	H	0.040302
36	H	0.033364
37	H	0.025763
38	C	0.036837
39	C	0.018586
40	C	-0.034565
41	H	0.04109
42	C	-0.015836

43	H	0.049781
44	C	-0.029068
45	H	0.048359
46	C	0.020285
47	C	-0.084166
48	C	-0.087547
49	H	0.019029
50	C	-0.012519
51	C	-0.080759
52	C	-0.021292
53	C	0.016797
54	C	-0.047121
55	C	0.008021
56	C	-0.060421
57	C	-0.078247
58	C	0.21528
59	C	0.217635
60	O	-0.119975
61	O	-0.250892
62	O	-0.120439
63	O	-0.254269
64	C	0.042517
65	C	-0.078192
66	C	0.04743
67	C	-0.079993
68	H	0.025455
69	H	0.014744
70	H	0.033523
71	H	0.035697
72	H	0.0419
73	H	0.024709
74	H	0.044832
75	H	0.038745
76	H	0.032836
77	H	0.03448
78	H	0.042721
79	H	0.029468
80	H	0.040213
81	H	0.037838
82	H	0.028872
83	H	0.041137
84	H	0.037084
85	C	-0.03931
86	C	0.014382
87	C	0.235326
88	C	0.213908

89	C	-0.023436
90	C	-0.047264
91	C	-0.089713
92	O	-0.244318
93	O	-0.110194
94	O	-0.222826
95	O	-0.084595
96	C	0.04811
97	C	-0.079823
98	C	0.052206
99	C	-0.077428
100	H	0.074727
101	H	0.052153
102	H	0.046823
103	H	0.092352
104	H	0.039525
105	H	0.043761
106	H	0.048365
107	H	0.029094
108	H	0.036517
109	H	0.041076
110	H	0.041859
111	H	0.036279
112	H	0.044606
113	H	0.033934
114	H	0.044911
115	H	0.034951
116	C	-0.072449
117	H	0.041349
118	H	0.039919
119	H	0.041308
120	C	-0.083875
121	H	0.031716
122	H	0.04049
123	H	0.02502
124	C	-0.080196
125	H	0.04307
126	H	0.028178
127	H	0.02741
128	O	-0.303541
129	C	-0.080159
130	H	0.040028
131	H	0.021023
132	H	0.03669
133	C	0.013011
134	H	0.020328

135	C	0.293593
136	F	-0.0819
137	F	-0.093166
138	F	-0.095483
139	C	0.290373
140	F	-0.089701
141	F	-0.101221
142	F	-0.099261
143	H	0.035989
144	H	0.034774
145	H	0.019387
146	H	0.041197
147	H	0.048175
	Tot	0.999652

Mo22 4r-1α

1	Mo	0.600204
2	N	-0.172792
3	C	0.020546
4	N	0.038382
5	C	0.078202
6	H	0.042234
7	C	-0.049693
8	H	0.034691
9	C	0.031247
10	C	0.010581
11	C	-0.03089
12	H	0.044342
13	C	-0.021669
14	C	-0.033991
15	H	0.042507
16	C	0.005577
17	C	0.052753
18	C	-0.022552
19	C	-0.021912
20	H	0.048774
21	C	-0.005986
22	H	0.058948
23	C	-0.008362
24	H	0.057058
25	C	-0.017827
26	C	0.301834
27	C	-0.080141
28	H	0.02595
29	C	-0.013231
30	C	-0.079688

31	C	-0.017147
32	C	0.014659
33	C	-0.047608
34	C	0.008182
35	C	-0.063063
36	C	-0.073754
37	C	0.210879
38	C	0.21764
39	O	-0.117062
40	O	-0.256995
41	O	-0.118504
42	O	-0.25624
43	C	0.044413
44	C	-0.078769
45	C	0.048491
46	C	-0.07896
47	H	0.027772
48	H	0.006567
49	H	0.030611
50	H	0.036595
51	H	0.037534
52	H	0.042672
53	H	0.045524
54	H	0.039051
55	H	0.03422
56	H	0.035295
57	H	0.043344
58	H	0.030466
59	H	0.041596
60	H	0.038485
61	H	0.027848
62	H	0.042091
63	H	0.037589
64	C	-0.037546
65	C	0.015229
66	C	0.241771
67	C	0.213831
68	C	-0.023072
69	C	-0.048527
70	C	-0.088773
71	O	-0.233637
72	O	-0.107204
73	O	-0.216931
74	O	-0.075446
75	C	0.049888
76	C	-0.078315

77	C	0.052947
78	C	-0.07237
79	H	0.06635
80	H	0.053569
81	H	0.049671
82	H	0.093333
83	H	0.044397
84	H	0.048487
85	H	0.041287
86	H	0.034895
87	H	0.042741
88	H	0.045027
89	H	0.043279
90	H	0.039175
91	H	0.044361
92	H	0.036436
93	H	0.045699
94	H	0.036596
95	C	-0.012721
96	H	0.012674
97	C	-0.012039
98	H	0.013955
99	Br	-0.213841
100	F	-0.095271
101	F	-0.090067
102	F	-0.095123
103	H	0.043631
104	C	0.017828
105	C	-0.079365
106	H	0.028304
107	H	0.011848
108	H	0.03372
109	C	-0.076947
110	H	0.033249
111	H	0.029628
112	H	0.032757
113	C	-0.07776
114	H	0.034607
115	H	0.030933
116	H	0.025738
117	C	-0.087481
118	H	0.011068
119	H	0.025915
120	H	0.029214
121	C	-0.081969
122	H	0.021519

123	H	0.022197
124	H	0.03866
125	C	-0.086361
126	H	0.013083
127	H	0.025282
128	H	0.037855
129	C	-0.080452
130	H	0.035672
131	H	0.029362
132	H	0.042364
133	C	-0.080932
134	H	0.035695
135	H	0.044731
136	H	0.0332
137	H	0.04999
	Tot	1.000014

Mo1 4r-1β

1	Mo	0.650261
2	N	-0.15738
3	C	-0.019093
4	N	0.025432
5	C	0.013302
6	H	0.076624
7	N	0.022358
8	C	0.009632
9	H	0.074807
10	C	0.020671
11	C	0.011994
12	C	-0.030182
13	H	0.046009
14	C	0.025628
15	C	-0.041131
16	H	0.038728
17	C	-0.002971
18	C	0.021811
19	C	0.006251
20	C	-0.038204
21	H	0.042017
22	C	0.022392
23	C	-0.039409
24	H	0.041593
25	C	0.006942
26	C	-0.083845
27	H	0.017489
28	H	0.027251

29	H	0.044684
30	C	-0.073562
31	H	0.037792
32	H	0.044664
33	H	0.034979
34	C	-0.078861
35	H	0.040605
36	H	0.025159
37	H	0.042089
38	C	0.035618
39	C	0.022825
40	C	-0.032243
41	H	0.045207
42	C	-0.015339
43	H	0.049907
44	C	-0.030086
45	H	0.048336
46	C	0.021738
47	C	-0.078951
48	H	0.040121
49	H	0.040909
50	H	0.027643
51	C	-0.078591
52	H	0.024921
53	H	0.035309
54	H	0.042416
55	C	-0.083215
56	H	0.040211
57	C	-0.011718
58	C	0.021185
59	C	-0.111929
60	C	0.024256
61	C	-0.046127
62	C	0.008161
63	C	-0.056936
64	C	-0.073071
65	C	0.217832
66	C	0.215807
67	O	-0.119017
68	O	-0.256768
69	O	-0.120845
70	O	-0.253814
71	C	0.04416
72	C	-0.079305
73	C	0.048694
74	C	-0.080095

75	H	0.03044
76	H	0.016768
77	H	0.037405
78	H	0.036265
79	H	0.040244
80	H	0.043871
81	H	0.040182
82	H	0.044929
83	H	0.03412
84	H	0.033481
85	H	0.043978
86	H	0.041094
87	H	0.027709
88	H	0.035777
89	H	0.041833
90	H	0.037165
91	H	0.036719
92	C	-0.028482
93	C	0.017141
94	C	0.22052
95	C	0.215541
96	C	-0.021763
97	C	-0.037969
98	C	-0.098765
99	O	-0.246383
100	O	-0.100876
101	O	-0.253503
102	O	-0.106641
103	C	0.04407
104	C	-0.075728
105	C	0.049902
106	C	-0.079593
107	H	0.067792
108	H	0.054418
109	H	0.051133
110	H	0.088704
111	H	0.047943
112	H	0.032349
113	H	0.037678
114	H	0.044552
115	H	0.034634
116	H	0.034966
117	H	0.042621
118	H	0.030369
119	H	0.034264
120	H	0.037954

121	H	0.040914
122	H	0.036185
123	C	-0.070936
124	H	0.04202
125	H	0.038984
126	H	0.040283
127	C	-0.088213
128	H	0.01474
129	H	0.038876
130	H	0.039397
131	C	-0.082403
132	H	0.019054
133	H	0.042879
134	H	0.02869
135	O	-0.294886
136	S	0.514945
137	O	-0.292355
138	O	-0.298645
139	C	0.284398
140	F	-0.078566
141	F	-0.082013
142	F	-0.085875
	Tot	1.000035

Mo2 4r-1β

1	Mo	0.655521
2	N	-0.155126
3	C	-0.020784
4	N	0.024665
5	C	0.013344
6	H	0.076201
7	N	0.023496
8	C	0.01051
9	H	0.074919
10	C	0.018841
11	C	0.014379
12	C	-0.030097
13	H	0.046675
14	C	0.026419
15	C	-0.043205
16	H	0.037315
17	C	-0.000397
18	C	0.021353
19	C	0.009644
20	C	-0.036199
21	H	0.043006

22	C	0.021423
23	C	-0.041089
24	H	0.039743
25	C	0.005892
26	C	-0.082316
27	H	0.025208
28	H	0.02803
29	H	0.044552
30	C	-0.072632
31	H	0.04065
32	H	0.041516
33	H	0.03485
34	C	-0.081236
35	H	0.037987
36	H	0.024967
37	H	0.039591
38	C	0.045383
39	C	0.026736
40	C	-0.028201
41	H	0.042998
42	C	-0.014973
43	H	0.05028
44	C	-0.023386
45	H	0.052841
46	C	-0.022443
47	C	0.028958
48	C	-0.084006
49	H	0.03835
50	C	-0.011542
51	C	0.020106
52	C	-0.106071
53	C	0.026648
54	C	-0.04469
55	C	0.007488
56	C	-0.06003
57	C	-0.074279
58	C	0.215244
59	C	0.217359
60	O	-0.119168
61	O	-0.258261
62	O	-0.119239
63	O	-0.252524
64	C	0.04252
65	C	-0.078967
66	C	0.048718
67	C	-0.080235

68	H	0.03018
69	H	0.014466
70	H	0.039461
71	H	0.036121
72	H	0.03943
73	H	0.042859
74	H	0.040017
75	H	0.045685
76	H	0.032981
77	H	0.03378
78	H	0.044238
79	H	0.036009
80	H	0.029957
81	H	0.035392
82	H	0.041862
83	H	0.038243
84	H	0.036946
85	C	-0.029081
86	C	0.017228
87	C	0.222248
88	C	0.217314
89	C	-0.021098
90	C	-0.035958
91	C	-0.09723
92	O	-0.246007
93	O	-0.100771
94	O	-0.24803
95	O	-0.106719
96	C	0.045972
97	C	-0.074963
98	C	0.05046
99	C	-0.080478
100	H	0.066937
101	H	0.054742
102	H	0.051384
103	H	0.088535
104	H	0.049
105	H	0.030182
106	H	0.037709
107	H	0.045334
108	H	0.033773
109	H	0.034774
110	H	0.042857
111	H	0.032295
112	H	0.034038
113	H	0.038819

114	H	0.042135
115	H	0.03667
116	C	-0.071583
117	H	0.037848
118	H	0.040109
119	H	0.041229
120	C	-0.086633
121	H	0.017194
122	H	0.034148
123	H	0.041941
124	C	-0.08384
125	H	0.02171
126	H	0.042778
127	H	0.02679
128	O	-0.297729
129	S	0.514428
130	O	-0.295476
131	O	-0.299363
132	C	0.283195
133	F	-0.083191
134	F	-0.081656
135	F	-0.088243
136	C	-0.076935
137	H	0.036443
138	H	0.030839
139	H	0.027963
140	C	-0.089311
141	H	0.007451
142	H	0.032739
143	H	0.021449
144	C	-0.081918
145	H	0.017534
146	H	0.029613
147	H	0.035196
148	H	0.042175
	Tot	0.99982

Mo3 4r-1β

1	Mo	0.657901
2	N	-0.179403
3	C	-0.017268
4	N	0.026224
5	C	0.014015
6	H	0.076613
7	N	0.022933
8	C	0.010817

9	H	0.07486
10	C	0.019953
11	C	0.012149
12	C	-0.030697
13	H	0.045819
14	C	0.025546
15	C	-0.041674
16	H	0.03882
17	C	-0.002503
18	C	0.020731
19	C	0.004854
20	C	-0.04433
21	H	0.040063
22	C	0.017619
23	C	-0.044418
24	H	0.039548
25	C	0.003355
26	C	-0.086501
27	H	0.017562
28	H	0.023481
29	H	0.044645
30	C	-0.074454
31	H	0.037759
32	H	0.04567
33	H	0.033767
34	C	-0.085829
35	H	0.038347
36	H	0.019723
37	H	0.038128
38	C	0.031821
39	C	0.040051
40	C	-0.028322
41	H	0.056353
42	C	-0.007318
43	H	0.057829
44	C	-0.026494
45	H	0.05987
46	C	0.039711
47	C	-0.081077
48	H	0.040954
49	C	-0.013676
50	C	0.022389
51	C	-0.111066
52	C	0.026383
53	C	-0.046177
54	C	0.007843

55	C	-0.057955
56	C	-0.072785
57	C	0.216308
58	C	0.21593
59	O	-0.12001
60	O	-0.258003
61	O	-0.120767
62	O	-0.252828
63	C	0.043726
64	C	-0.079261
65	C	0.048761
66	C	-0.080262
67	H	0.031286
68	H	0.011401
69	H	0.037254
70	H	0.036426
71	H	0.041116
72	H	0.043874
73	H	0.04021
74	H	0.044829
75	H	0.034056
76	H	0.033456
77	H	0.043883
78	H	0.041397
79	H	0.025984
80	H	0.036286
81	H	0.041877
82	H	0.037493
83	H	0.036471
84	C	-0.027558
85	C	0.017194
86	C	0.220892
87	C	0.216023
88	C	-0.021694
89	C	-0.038117
90	C	-0.098064
91	O	-0.245727
92	O	-0.100622
93	O	-0.25096
94	O	-0.106289
95	C	0.04455
96	C	-0.075563
97	C	0.050145
98	C	-0.079143
99	H	0.06659
100	H	0.054511

101	H	0.051263
102	H	0.088994
103	H	0.049655
104	H	0.03275
105	H	0.037767
106	H	0.044653
107	H	0.035088
108	H	0.035212
109	H	0.043023
110	H	0.030875
111	H	0.034651
112	H	0.038222
113	H	0.041418
114	H	0.036465
115	C	-0.071139
116	H	0.042037
117	H	0.03904
118	H	0.040041
119	C	-0.087577
120	H	0.015146
121	H	0.038492
122	H	0.040257
123	C	-0.090304
124	H	0.010579
125	H	0.041048
126	H	0.024518
127	O	-0.294782
128	S	0.516451
129	O	-0.29259
130	O	-0.299648
131	C	0.28507
132	F	-0.079704
133	F	-0.082142
134	F	-0.08564
135	Cl	0.01777
136	Cl	0.033852
	Tot	1.000031

Mo10 4r-1β

1	Mo	0.634693
2	N	-0.164851
3	C	-0.021115
4	N	0.063014
5	N	0.011212
6	C	0.107781
7	C	0.039331

8	C	-0.041559
9	C	-0.020095
10	H	0.046437
11	C	-0.012509
12	C	-0.005268
13	H	0.057412
14	C	-0.021848
15	C	0.041219
16	C	-0.032153
17	C	-0.015655
18	H	0.053956
19	C	-0.014655
20	C	-0.015711
21	H	0.051975
22	C	-0.029419
23	C	0.032869
24	C	0.017016
25	C	-0.033752
26	H	0.047389
27	C	-0.015626
28	H	0.05179
29	C	-0.034168
30	H	0.045516
31	C	0.022953
32	C	-0.07796
33	H	0.035742
34	H	0.044257
35	H	0.023382
36	C	-0.079676
37	H	0.03973
38	H	0.04166
39	H	0.026094
40	C	-0.082947
41	H	0.038454
42	C	-0.013342
43	C	0.027178
44	C	-0.113457
45	C	0.028373
46	C	-0.04458
47	C	0.006747
48	C	-0.056806
49	C	-0.072579
50	C	0.215937
51	C	0.216225
52	O	-0.121992
53	O	-0.256776

54	O	-0.118839
55	O	-0.252716
56	C	0.046024
57	C	-0.079175
58	C	0.049141
59	C	-0.080658
60	H	0.029145
61	H	0.018558
62	H	0.040805
63	H	0.037116
64	H	0.044713
65	H	0.038043
66	H	0.04022
67	H	0.045691
68	H	0.044128
69	H	0.031443
70	H	0.033419
71	H	0.040982
72	H	0.034339
73	H	0.035194
74	H	0.042491
75	H	0.037827
76	H	0.042658
77	C	-0.029039
78	C	0.017189
79	C	0.220016
80	C	0.219756
81	C	-0.021664
82	C	-0.034507
83	C	-0.09718
84	O	-0.237629
85	O	-0.101715
86	O	-0.254453
87	O	-0.10328
88	C	0.048613
89	C	-0.081305
90	C	0.048533
91	C	-0.076363
92	H	0.069468
93	H	0.054961
94	H	0.05191
95	H	0.088667
96	H	0.049517
97	H	0.030398
98	H	0.039439
99	H	0.036813

100	H	0.037006
101	H	0.041623
102	H	0.036087
103	H	0.045778
104	H	0.033518
105	H	0.02897
106	H	0.034373
107	H	0.041344
108	O	-0.297919
109	S	0.5075
110	O	-0.306647
111	O	-0.306766
112	C	0.278763
113	F	-0.081175
114	F	-0.099567
115	F	-0.089112
116	H	0.052827
117	H	0.050366
118	H	0.035744
119	H	0.048661
120	H	0.052824
121	H	0.041995
122	N	-0.108677
123	C	-0.0141
124	C	-0.022068
125	C	-0.031688
126	C	-0.021021
127	C	-0.023642
128	C	-0.01247
129	H	0.047242
130	H	0.035013
131	H	0.052552
132	H	0.05083
133	H	0.053361
	Tot	1.000061

Mo13 4r-1β

1	Mo	0.654185
2	N	-0.198453
3	C	0.026688
4	N	-0.012693
5	C	0.014032
6	H	0.046327
7	N	-0.009892
8	C	0.015475
9	H	0.044229

10	C	0.018283
11	C	0.006137
12	C	-0.042423
13	H	0.040708
14	C	0.019754
15	C	-0.042241
16	H	0.038661
17	C	0.00181
18	C	0.018499
19	C	0.005234
20	C	-0.042123
21	H	0.040747
22	C	0.019545
23	C	-0.046717
24	H	0.039393
25	C	0.001526
26	C	-0.085984
27	H	0.021568
28	H	0.02831
29	H	0.043468
30	C	-0.07322
31	H	0.039452
32	H	0.04072
33	H	0.037752
34	C	-0.085554
35	H	0.018332
36	H	0.032552
37	H	0.038011
38	C	0.051458
39	C	-0.020298
40	C	-0.017201
41	H	0.050973
42	C	-0.011381
43	H	0.056573
44	C	-0.009374
45	H	0.056538
46	C	-0.021823
47	C	-0.086
48	H	0.024907
49	C	-0.010602
50	C	0.012933
51	C	-0.115646
52	C	0.019486
53	C	-0.047273
54	C	0.006507
55	C	-0.057559

56	C	-0.078582
57	C	0.217645
58	C	0.2184
59	O	-0.117497
60	O	-0.260707
61	O	-0.1186
62	O	-0.24573
63	C	0.04935
64	C	-0.084971
65	C	0.04814
66	C	-0.078916
67	H	0.02719
68	H	0.015689
69	H	0.037805
70	H	0.035209
71	H	0.028615
72	H	0.037627
73	H	0.04528
74	H	0.037094
75	H	0.035203
76	H	0.035388
77	H	0.043514
78	H	0.041466
79	H	0.045545
80	H	0.023405
81	H	0.041763
82	H	0.029136
83	H	0.039848
84	C	-0.029317
85	C	0.014735
86	C	0.217702
87	C	0.212297
88	C	-0.026197
89	C	-0.049604
90	C	-0.096366
91	O	-0.251474
92	O	-0.103285
93	O	-0.249869
94	O	-0.105245
95	C	0.045851
96	C	-0.078148
97	C	0.048566
98	C	-0.078768
99	H	0.065675
100	H	0.050847
101	H	0.048142

102	H	0.090869
103	H	0.043791
104	H	0.034455
105	H	0.045226
106	H	0.03442
107	H	0.034236
108	H	0.042571
109	H	0.038069
110	H	0.04303
111	H	0.031179
112	H	0.041674
113	H	0.027202
114	H	0.038784
115	C	-0.073735
116	H	0.042184
117	H	0.037544
118	H	0.037605
119	C	-0.084244
120	H	0.03991
121	H	0.025222
122	H	0.032079
123	C	-0.081196
124	H	0.041852
125	H	0.026599
126	H	0.027342
127	O	-0.251829
128	C	0.057644
129	C	0.068419
130	C	0.079026
131	C	0.081335
132	C	0.082172
133	C	0.08093
134	C	0.301234
135	F	-0.083283
136	F	-0.105348
137	F	-0.096729
138	H	0.043429
139	F	-0.060278
140	F	-0.083215
141	F	-0.085241
142	F	-0.088039
143	F	-0.076158
144	C	-0.041282
145	H	0.050471
146	H	0.036535
147	H	0.054098

148	H	0.041349

Mo14 4r-1β

1	Mo	0.650671
2	N	-0.180486
3	C	0.026449
4	N	-0.013061
5	C	0.01367
6	H	0.046591
7	N	-0.010088
8	C	0.014918
9	H	0.043535
10	C	0.018422
11	C	0.005682
12	C	-0.043958
13	H	0.039369
14	C	0.018139
15	C	-0.043681
16	H	0.039071
17	C	0.000185
18	C	0.021072
19	C	0.005799
20	C	-0.042341
21	H	0.040891
22	C	0.021558
23	C	-0.039071
24	H	0.040821
25	C	0.008114
26	C	-0.085947
27	H	0.018749
28	H	0.031346
29	H	0.043308
30	C	-0.071688
31	H	0.041193
32	H	0.041343
33	H	0.038125
34	C	-0.082728
35	H	0.022209
36	H	0.035022
37	H	0.038502
38	C	0.036175
39	C	0.02184
40	C	-0.034251
41	H	0.04417
42	C	-0.0145
43	H	0.051647

44	C	-0.031987
45	H	0.047374
46	C	0.019349
47	C	-0.089013
48	H	0.02451
49	C	-0.011029
50	C	0.011634
51	C	-0.114662
52	C	0.016737
53	C	-0.047379
54	C	0.006521
55	C	-0.054654
56	C	-0.078316
57	C	0.217715
58	C	0.218605
59	O	-0.119493
60	O	-0.257899
61	O	-0.117887
62	O	-0.245398
63	C	0.048492
64	C	-0.084315
65	C	0.048477
66	C	-0.079142
67	H	0.029363
68	H	0.0203
69	H	0.037342
70	H	0.035313
71	H	0.029423
72	H	0.038238
73	H	0.045745
74	H	0.037404
75	H	0.035045
76	H	0.034863
77	H	0.04363
78	H	0.041444
79	H	0.044276
80	H	0.025833
81	H	0.041797
82	H	0.028579
83	H	0.039668
84	C	-0.031358
85	C	0.014795
86	C	0.217546
87	C	0.212561
88	C	-0.026575
89	C	-0.049605

90	C	-0.096793
91	O	-0.250919
92	O	-0.103022
93	O	-0.247752
94	O	-0.106379
95	C	0.045714
96	C	-0.078429
97	C	0.048891
98	C	-0.079123
99	H	0.064151
100	H	0.050818
101	H	0.046992
102	H	0.09074
103	H	0.040096
104	H	0.033273
105	H	0.045193
106	H	0.034177
107	H	0.032179
108	H	0.042599
109	H	0.038784
110	H	0.043005
111	H	0.030832
112	H	0.041686
113	H	0.026505
114	H	0.038391
115	C	-0.075106
116	H	0.04066
117	H	0.038286
118	H	0.034285
119	C	-0.08491
120	H	0.040831
121	H	0.025728
122	H	0.028511
123	C	-0.080141
124	H	0.040947
125	H	0.029036
126	H	0.027661
127	O	-0.25556
128	C	0.057114
129	C	0.0676
130	C	0.077584
131	C	0.080703
132	C	0.081014
133	C	0.080734
134	F	-0.061026
135	F	-0.081099

136	F	-0.085745
137	F	-0.088622
138	F	-0.076532
139	C	-0.041045
140	H	0.049685
141	H	0.036659
142	H	0.054285
143	H	0.041374
144	C	-0.08365
145	H	0.02274
146	H	0.033481
147	H	0.035651
148	C	-0.077598
149	H	0.041838
150	H	0.037758
151	H	0.024549
	Tot	0.999948

Mo15 4r-1β

1	Mo	0.65815
2	N	-0.196466
3	C	0.02658
4	N	-0.010866
5	C	0.014849
6	H	0.049793
7	N	-0.011012
8	C	0.015473
9	H	0.048838
10	C	0.02444
11	C	0.008386
12	C	-0.038983
13	H	0.04308
14	C	0.020508
15	C	-0.03939
16	H	0.040543
17	C	0.005627
18	C	0.018461
19	C	0.00805
20	C	-0.041449
21	H	0.04037
22	C	0.018769
23	C	-0.049455
24	H	0.036526
25	C	0.000835
26	C	-0.088486
27	H	0.042014

28	H	0.020874
29	H	0.022817
30	C	-0.07482
31	H	0.039612
32	H	0.04128
33	H	0.032975
34	C	-0.084418
35	H	0.037293
36	H	0.035045
37	H	0.022265
38	C	0.025848
39	C	0.104145
40	C	0.082422
41	C	0.095642
42	C	0.081311
43	C	0.097284
44	C	-0.084894
45	H	0.035232
46	C	-0.010713
47	C	0.012938
48	C	-0.126118
49	C	0.021483
50	C	-0.044899
51	C	0.00597
52	C	-0.065302
53	C	-0.0744
54	C	0.214373
55	C	0.224102
56	O	-0.125495
57	O	-0.254115
58	O	-0.113979
59	O	-0.233879
60	C	0.041422
61	C	-0.079475
62	C	0.04863
63	C	-0.079016
64	H	0.01911
65	H	0.013173
66	H	0.039026
67	H	0.03625
68	H	0.044958
69	H	0.033092
70	H	0.037495
71	H	0.047282
72	H	0.045491
73	H	0.033819

74	H	0.036561
75	H	0.032193
76	H	0.03717
77	H	0.032901
78	H	0.043882
79	H	0.037613
80	H	0.039166
81	C	-0.037031
82	C	0.016243
83	C	0.223893
84	C	0.215989
85	C	-0.025735
86	C	-0.042192
87	C	-0.098972
88	O	-0.247133
89	O	-0.117339
90	O	-0.232167
91	O	-0.105754
92	C	0.049873
93	C	-0.081969
94	C	0.047075
95	C	-0.084476
96	H	0.050688
97	H	0.045093
98	H	0.047762
99	H	0.089135
100	H	0.028557
101	H	0.040476
102	H	0.02716
103	H	0.046157
104	H	0.034135
105	H	0.039814
106	H	0.026411
107	H	0.043858
108	H	0.045558
109	H	0.029546
110	H	0.044416
111	H	0.029431
112	C	-0.07152
113	H	0.043892
114	H	0.03897
115	H	0.040202
116	C	-0.082062
117	H	0.020032
118	H	0.040024
119	H	0.042641

120	C	-0.088237
121	H	0.044293
122	H	0.014843
123	H	0.031214
124	O	-0.292028
125	C	-0.039925
126	H	0.042116
127	H	0.039836
128	H	0.039086
129	H	0.053359
130	F	-0.053985
131	F	-0.071101
132	F	-0.0725
133	F	-0.071864
134	F	-0.058484
135	C	0.057435
136	C	-0.09731
137	H	0.029861
138	H	0.033835
139	H	0.041059
140	C	0.290831
141	F	-0.097701
142	F	-0.082428
143	F	-0.083032
144	C	0.29512
145	F	-0.092333
146	F	-0.085164
147	F	-0.093234
	Tot	1.000044

Mo11 4r-1β

1	Mo	0.648262
2	N	-0.181081
3	C	-0.016373
4	N	0.019855
5	C	0.000029
6	H	0.067244
7	N	0.020504
8	C	0.001488
9	H	0.067653
10	C	0.03297
11	C	0.01821
12	C	-0.040403
13	H	0.041942
14	C	-0.025152
15	H	0.048038

16	C	-0.038449
17	H	0.044798
18	C	0.011191
19	C	-0.082418
20	H	0.033987
21	C	-0.01223
22	C	0.023099
23	C	-0.114123
24	C	0.01515
25	C	-0.046182
26	C	0.005068
27	C	-0.062853
28	C	-0.080881
29	C	0.216222
30	C	0.222444
31	O	-0.121561
32	O	-0.256194
33	O	-0.114311
34	O	-0.241965
35	C	0.045564
36	C	-0.080269
37	C	0.048142
38	C	-0.079293
39	H	0.019867
40	H	0.018727
41	H	0.038241
42	H	0.034587
43	H	0.038507
44	H	0.026119
45	H	0.037143
46	H	0.046411
47	H	0.032813
48	H	0.036475
49	H	0.044378
50	H	0.039791
51	H	0.0359
52	H	0.042222
53	H	0.037686
54	H	0.032053
55	H	0.038011
56	C	-0.03368
57	C	0.01601
58	C	0.22323
59	C	0.219407
60	C	-0.026824
61	C	-0.035084

62	C	-0.088078
63	O	-0.234379
64	O	-0.114827
65	O	-0.231776
66	O	-0.098206
67	C	0.04849
68	C	-0.07818
69	C	0.048599
70	C	-0.080059
71	H	0.060947
72	H	0.051017
73	H	0.046761
74	H	0.092383
75	H	0.030272
76	H	0.040247
77	H	0.044121
78	H	0.036725
79	H	0.041452
80	H	0.035929
81	H	0.033188
82	H	0.044706
83	H	0.037569
84	H	0.036778
85	H	0.044377
86	H	0.037975
87	O	-0.254084
88	C	0.045271
89	H	0.028005
90	C	-0.078831
91	H	0.041825
92	H	0.035405
93	H	0.023335
94	C	-0.074226
95	H	0.035712
96	H	0.046453
97	H	0.039358
98	C	0.04514
99	H	0.025196
100	C	-0.08613
101	H	0.040121
102	H	0.022282
103	H	0.015139
104	C	-0.073112
105	H	0.038167
106	H	0.036815
107	H	0.048154

108	C	-0.08071
109	H	0.040081
110	H	0.031215
111	H	0.023548
112	C	-0.087203
113	H	0.03246
114	H	0.034113
115	H	0.026407
116	C	0.076046
117	C	-0.01131
118	C	-0.01205
119	C	-0.033762
120	C	-0.03381
121	C	-0.044354
122	H	0.041803
123	H	0.040847
124	H	0.044219
125	C	-0.012038
126	C	0.010594
127	C	0.003768
128	C	-0.050472
129	C	-0.049194
130	C	0.00915
131	H	0.026622
132	H	0.034267
133	C	-0.01177
134	C	0.004297
135	C	0.012186
136	C	-0.053116
137	C	-0.047108
138	C	0.009797
139	H	0.032116
140	H	0.034352
141	C	-0.081866
142	H	0.023799
143	H	0.030866
144	H	0.039884
145	C	-0.08836
146	H	0.026749
147	H	0.023594
148	H	0.032121
149	C	-0.07536
150	H	0.032483
151	H	0.038716
152	H	0.035331
153	C	-0.082994

154	H	0.036216
155	H	0.02329
156	H	0.032413
157	C	-0.08715
158	H	0.017867
159	H	0.037475
160	H	0.024723
161	C	-0.075382
162	H	0.039895
163	H	0.032915
164	H	0.03548
	Tot	0.99983

Mo4 4r-1β

1	Mo	0.640588
2	N	-0.193604
3	C	-0.016704
4	N	0.025037
5	C	0.009367
6	H	0.073266
7	N	0.022032
8	C	0.006801
9	H	0.073418
10	C	0.027613
11	C	0.00966
12	C	-0.035597
13	H	0.042655
14	C	0.022485
15	C	-0.036807
16	H	0.040035
17	C	0.004969
18	C	0.024191
19	C	0.00768
20	C	-0.038886
21	H	0.040663
22	C	0.020885
23	C	-0.039718
24	H	0.040371
25	C	0.0073
26	C	-0.07874
27	H	0.026917
28	H	0.033218
29	H	0.043146
30	C	-0.072977
31	H	0.037611
32	H	0.043717

33	H	0.036289
34	C	-0.081798
35	H	0.036152
36	H	0.026631
37	H	0.039258
38	C	0.034207
39	C	0.017093
40	C	-0.035789
41	H	0.041613
42	C	-0.023555
43	H	0.046001
44	C	-0.032795
45	H	0.046788
46	C	0.017308
47	C	-0.083138
48	H	0.035364
49	H	0.034893
50	H	0.023103
51	C	-0.083825
52	H	0.019466
53	H	0.038165
54	H	0.035764
55	C	-0.083585
56	H	0.034221
57	C	-0.013778
58	C	0.012636
59	C	-0.118171
60	C	0.019526
61	C	-0.047014
62	C	0.008009
63	C	-0.056742
64	C	-0.077724
65	C	0.217306
66	C	0.218794
67	O	-0.116066
68	O	-0.251289
69	O	-0.118566
70	O	-0.247527
71	C	0.043525
72	C	-0.079331
73	C	0.048685
74	C	-0.078419
75	H	0.028114
76	H	0.017997
77	H	0.037839
78	H	0.033052

79	H	0.037142
80	H	0.039039
81	H	0.045578
82	H	0.036931
83	H	0.035177
84	H	0.036227
85	H	0.04387
86	H	0.028188
87	H	0.040391
88	H	0.040766
89	H	0.036849
90	H	0.028308
91	H	0.036805
92	C	-0.028439
93	C	0.01485
94	C	0.216139
95	C	0.222039
96	C	-0.025533
97	C	-0.040735
98	C	-0.094297
99	O	-0.236165
100	O	-0.097866
101	O	-0.234554
102	O	-0.116059
103	C	0.047197
104	C	-0.080186
105	C	0.044252
106	C	-0.082878
107	H	0.065388
108	H	0.050545
109	H	0.051515
110	H	0.091271
111	H	0.039364
112	H	0.040404
113	H	0.025514
114	H	0.042294
115	H	0.039711
116	H	0.031125
117	H	0.032878
118	H	0.039947
119	H	0.030055
120	H	0.030632
121	H	0.037826
122	H	0.040087
123	C	-0.072026
124	H	0.03748

125	H	0.038915
126	H	0.042404
127	C	-0.082221
128	H	0.034283
129	H	0.039501
130	H	0.025693
131	C	-0.077023
132	H	0.0302
133	H	0.025277
134	H	0.043448
135	O	-0.243035
136	C	0.067314
137	C	-0.003249
138	C	-0.000937
139	C	-0.047648
140	C	-0.047768
141	C	0.000394
142	H	0.033481
143	H	0.033782
144	C	-0.081609
145	H	0.017376
146	H	0.034107
147	H	0.033368
148	C	-0.09073
149	H	0.025996
150	H	0.03013
151	H	0.01676
152	C	-0.07728
153	H	0.03474
154	H	0.033608
155	H	0.034411
	Tot	1.000019

Mo5 4r-1β

1	Mo	0.652179
2	N	-0.205565
3	C	-0.014556
4	N	0.025929
5	C	0.011205
6	H	0.074731
7	N	0.023631
8	C	0.009965
9	H	0.074716
10	C	0.02219
11	C	0.002287
12	C	-0.045502

13	H	0.040632
14	C	0.018432
15	C	-0.040037
16	H	0.042442
17	C	0.003309
18	C	0.021126
19	C	0.005412
20	C	-0.042852
21	H	0.04178
22	C	0.016763
23	C	-0.045319
24	H	0.037739
25	C	0.002937
26	C	-0.078685
27	H	0.02624
28	H	0.032994
29	H	0.045404
30	C	-0.07397
31	H	0.034678
32	H	0.043974
33	H	0.037457
34	C	-0.086596
35	H	0.037425
36	H	0.018016
37	H	0.036673
38	C	0.030052
39	C	0.037699
40	C	-0.030139
41	H	0.053574
42	C	-0.010399
43	H	0.056726
44	C	-0.026267
45	H	0.059504
46	C	0.038081
47	C	-0.082525
48	H	0.030046
49	C	-0.015821
50	C	0.0179
51	C	-0.116317
52	C	0.023793
53	C	-0.04726
54	C	0.007023
55	C	-0.059131
56	C	-0.075776
57	C	0.213356
58	C	0.218618

59	O	-0.117456
60	O	-0.257286
61	O	-0.118821
62	O	-0.243547
63	C	0.042994
64	C	-0.078529
65	C	0.049246
66	C	-0.079112
67	H	0.028663
68	H	0.011487
69	H	0.03668
70	H	0.03459
71	H	0.038246
72	H	0.041908
73	H	0.046713
74	H	0.037744
75	H	0.034034
76	H	0.035407
77	H	0.044407
78	H	0.025885
79	H	0.04121
80	H	0.041418
81	H	0.038667
82	H	0.028879
83	H	0.036034
84	C	-0.026094
85	C	0.013985
86	C	0.214987
87	C	0.217337
88	C	-0.026353
89	C	-0.041096
90	C	-0.093112
91	O	-0.249464
92	O	-0.098173
93	O	-0.250906
94	O	-0.11191
95	C	0.045795
96	C	-0.080352
97	C	0.045459
98	C	-0.080805
99	H	0.069467
100	H	0.050468
101	H	0.051879
102	H	0.092201
103	H	0.041291
104	H	0.043649

105	H	0.019876
106	H	0.04655
107	H	0.042913
108	H	0.032327
109	H	0.035425
110	H	0.039548
111	H	0.026915
112	H	0.030835
113	H	0.037622
114	H	0.039979
115	C	-0.075217
116	H	0.040192
117	H	0.034229
118	H	0.040459
119	C	-0.084477
120	H	0.03645
121	H	0.040332
122	H	0.025837
123	C	-0.083185
124	H	0.024623
125	H	0.018183
126	H	0.042271
127	O	-0.250456
128	C	0.058161
129	C	0.076264
130	C	0.080104
131	C	0.081259
132	C	0.082078
133	C	0.077586
134	Cl	0.024691
135	Cl	0.005983
136	F	-0.06855
137	F	-0.078077
138	F	-0.086559
139	F	-0.085908
140	F	-0.085799
	Tot	1.000095

Mo19 4r-1β

1	Mo	0.609751
2	N	-0.164178
3	C	0.005604
4	N	0.029636
5	C	0.079326
6	C	-0.045714
7	H	0.03425

8	C	0.034445
9	C	0.012672
10	C	-0.029291
11	H	0.044848
12	C	-0.016212
13	C	-0.028974
14	H	0.044415
15	C	0.012721
16	C	0.04263
17	C	0.025053
18	C	-0.03261
19	H	0.044083
20	C	-0.012916
21	H	0.04999
22	C	-0.031367
23	H	0.045936
24	C	0.025393
25	C	-0.01374
26	C	-0.086115
27	H	0.025775
28	C	-0.008164
29	C	0.029319
30	C	-0.117417
31	C	0.043338
32	C	-0.043464
33	C	0.007136
34	C	-0.059191
35	C	-0.071973
36	C	0.217541
37	C	0.217427
38	O	-0.119616
39	O	-0.254259
40	O	-0.118887
41	O	-0.25336
42	C	0.043179
43	C	-0.07895
44	C	0.049051
45	C	-0.081112
46	H	0.025395
47	H	0.01769
48	H	0.04184
49	H	0.037134
50	H	0.043598
51	H	0.043268
52	H	0.039912
53	H	0.045826

54	H	0.029804
55	H	0.033768
56	H	0.043854
57	H	0.037416
58	H	0.03158
59	H	0.038213
60	H	0.034995
61	H	0.042036
62	H	0.039428
63	C	-0.02526
64	C	0.016936
65	C	0.215293
66	C	0.219483
67	C	-0.025363
68	C	-0.038976
69	C	-0.090669
70	O	-0.229306
71	O	-0.111523
72	O	-0.248433
73	O	-0.104962
74	C	0.047201
75	C	-0.079536
76	C	0.048751
77	C	-0.07827
78	H	0.065458
79	H	0.05056
80	H	0.049155
81	H	0.088155
82	H	0.046326
83	H	0.037847
84	H	0.046427
85	H	0.036192
86	H	0.043833
87	H	0.037197
88	H	0.036165
89	H	0.045071
90	H	0.031943
91	H	0.035581
92	H	0.043922
93	H	0.0359
94	C	-0.019721
95	H	0.004869
96	C	-0.01168
97	H	0.016166
98	C	-0.077717
99	H	0.030257

100	H	0.028438
101	H	0.03091
102	C	-0.077551
103	H	0.027344
104	H	0.031939
105	H	0.030591
106	C	-0.012214
107	H	0.012495
108	C	-0.086419
109	H	0.012776
110	H	0.029623
111	H	0.027402
112	C	-0.081696
113	H	0.026587
114	H	0.02405
115	H	0.030981
116	H	0.009297
117	Br	-0.187212
118	C	0.020626
119	H	0.050948
120	H	0.0437
121	C	-0.082465
122	H	0.016671
123	H	0.029937
124	H	0.02677
125	C	-0.076584
126	H	0.029903
127	H	0.035546
128	H	0.026983
129	C	-0.08489
130	H	0.028713
131	H	0.031822
132	H	0.01237
133	C	-0.076616
134	H	0.032993
135	H	0.030676
136	H	0.034673
137	C	-0.081467
138	H	0.045187
139	H	0.033078
140	H	0.036169
141	C	-0.08423
142	H	0.035231
143	H	0.02369
144	H	0.041237
145	C	-0.07543

146	H	0.04187
147	H	0.029198
148	H	0.030962
149	C	-0.08716
150	H	0.022469
151	H	0.034167
152	H	0.02683
	Tot	0.999959

Mo16 4r-1β

1	Mo	0.658932
2	N	-0.171173
3	C	0.018051
4	N	0.033102
5	C	0.080204
6	C	-0.046199
7	H	0.033863
8	C	0.035016
9	C	0.013421
10	C	-0.0306
11	H	0.045097
12	C	-0.016904
13	C	-0.027493
14	H	0.044627
15	C	0.011599
16	C	0.041482
17	C	0.02501
18	C	-0.032542
19	H	0.043238
20	C	-0.012547
21	H	0.05014
22	C	-0.031206
23	H	0.045734
24	C	0.025181
25	C	-0.012612
26	C	-0.086983
27	H	0.020492
28	C	-0.006764
29	C	0.028347
30	C	-0.128634
31	C	0.044301
32	C	-0.043271
33	C	0.006269
34	C	-0.060651
35	C	-0.075494
36	C	0.217094

37	C	0.216836
38	O	-0.117224
39	O	-0.25568
40	O	-0.119115
41	O	-0.253998
42	C	0.042801
43	C	-0.079099
44	C	0.048755
45	C	-0.08072
46	H	0.025537
47	H	0.016497
48	H	0.041523
49	H	0.036977
50	H	0.043178
51	H	0.043194
52	H	0.039344
53	H	0.04513
54	H	0.029679
55	H	0.034228
56	H	0.043774
57	H	0.036745
58	H	0.031134
59	H	0.038246
60	H	0.034332
61	H	0.041968
62	H	0.027912
63	C	-0.026783
64	C	0.015049
65	C	0.214427
66	C	0.219593
67	C	-0.026787
68	C	-0.037744
69	C	-0.089083
70	O	-0.22767
71	O	-0.111832
72	O	-0.251656
73	O	-0.104803
74	C	0.042005
75	C	-0.076474
76	C	0.049726
77	C	-0.077913
78	H	0.062514
79	H	0.051214
80	H	0.04757
81	H	0.089499
82	H	0.045385

83	H	0.037165
84	H	0.046978
85	H	0.037262
86	H	0.045099
87	H	0.037637
88	H	0.037371
89	H	0.037
90	H	0.029226
91	H	0.036937
92	H	0.046567
93	H	0.035
94	C	-0.011562
95	H	0.017211
96	C	-0.012557
97	H	0.015077
98	C	-0.078694
99	H	0.030947
100	H	0.028624
101	H	0.028136
102	C	-0.078029
103	H	0.026023
104	H	0.031286
105	H	0.030323
106	C	-0.011318
107	H	0.01384
108	C	-0.08534
109	H	0.011959
110	H	0.031138
111	H	0.029204
112	C	-0.081279
113	H	0.027074
114	H	0.024246
115	H	0.031497
116	H	0.011422
117	C	0.019832
118	H	0.051249
119	H	0.043872
120	C	-0.086656
121	H	0.01888
122	H	0.025612
123	H	0.02947
124	C	-0.076015
125	H	0.032954
126	H	0.036253
127	H	0.025166
128	C	-0.085535

129	H	0.028222
130	H	0.032065
131	H	0.01226
132	C	-0.076643
133	H	0.032934
134	H	0.029968
135	H	0.034378
136	C	-0.080413
137	H	0.046205
138	H	0.034436
139	H	0.035578
140	C	-0.083115
141	H	0.034874
142	H	0.030288
143	H	0.041858
144	C	-0.07711
145	H	0.040931
146	H	0.028187
147	H	0.031337
148	C	-0.08314
149	H	0.022297
150	H	0.035182
151	H	0.028582
152	C	0.05443
153	C	0.070738
154	C	0.072247
155	C	0.080386
156	C	0.077535
157	C	0.070896
158	F	-0.054288
159	F	-0.082302
160	F	-0.08359
161	F	-0.075589
162	F	-0.086994
163	O	-0.269673

Mo17 4r-1β

1	Mo	0.642356
2	N	-0.173921
3	C	0.015048
4	N	0.031542
5	C	0.080329
6	C	-0.045905
7	H	0.035236
8	C	0.035069
9	C	0.011583

10	C	-0.033748
11	H	0.0448
12	C	-0.020541
13	C	-0.03002
14	H	0.044887
15	C	0.011614
16	C	0.041749
17	C	0.023719
18	C	-0.027091
19	H	0.043833
20	C	-0.011099
21	H	0.052815
22	C	-0.02968
23	H	0.045339
24	C	-0.027842
25	C	0.030033
26	C	-0.081241
27	H	0.03035
28	C	-0.010531
29	C	0.028156
30	C	-0.134674
31	C	0.023662
32	C	-0.04931
33	C	0.007755
34	C	-0.055565
35	C	-0.076673
36	C	0.218591
37	C	0.216196
38	O	-0.117799
39	O	-0.25206
40	O	-0.11887
41	O	-0.24476
42	C	0.047381
43	C	-0.078944
44	C	0.047815
45	C	-0.079633
46	H	0.029981
47	H	0.019479
48	H	0.031623
49	H	0.034573
50	H	0.039164
51	H	0.041742
52	H	0.039415
53	H	0.045614
54	H	0.03096
55	H	0.036008

56	H	0.04295
57	H	0.043976
58	H	0.035564
59	H	0.036889
60	H	0.034731
61	H	0.042981
62	H	0.029339
63	C	-0.03171
64	C	0.016084
65	C	0.222562
66	C	0.217372
67	C	-0.022272
68	C	-0.039485
69	C	-0.090236
70	O	-0.242356
71	O	-0.100344
72	O	-0.250726
73	O	-0.108875
74	C	0.044639
75	C	-0.075256
76	C	0.050853
77	C	-0.08116
78	H	0.058866
79	H	0.053061
80	H	0.049043
81	H	0.084764
82	H	0.045929
83	H	0.0344
84	H	0.047398
85	H	0.040063
86	H	0.043167
87	H	0.031977
88	H	0.030582
89	H	0.03161
90	H	0.032644
91	H	0.038393
92	H	0.044009
93	H	0.036995
94	C	-0.009894
95	H	0.018502
96	C	-0.011795
97	H	0.014922
98	C	-0.08147
99	H	0.032632
100	H	0.023192
101	H	0.027488

102	C	-0.079497
103	H	0.027284
104	H	0.032607
105	H	0.029107
106	C	0.019238
107	H	0.049727
108	H	0.044258
109	C	-0.084841
110	H	0.021566
111	H	0.03068
112	H	0.029535
113	C	-0.076572
114	H	0.033608
115	H	0.037075
116	H	0.023758
117	C	-0.084836
118	H	0.028807
119	H	0.032584
120	H	0.016392
121	C	-0.076582
122	H	0.03366
123	H	0.029676
124	H	0.034499
125	C	-0.079512
126	H	0.046972
127	H	0.035847
128	H	0.035987
129	C	-0.082356
130	H	0.03585
131	H	0.031984
132	H	0.042786
133	C	-0.074362
134	H	0.043394
135	H	0.031514
136	H	0.033156
137	C	-0.079495
138	H	0.026825
139	H	0.038534
140	H	0.029433
141	C	0.053193
142	C	0.069336
143	C	0.069781
144	C	0.079315
145	C	0.07647
146	C	0.075087
147	F	-0.058255

148	F	-0.084825
149	F	-0.08701
150	F	-0.077497
151	F	-0.0844
152	C	-0.088794
153	H	0.018536
154	H	0.030322
155	H	0.018125
156	H	0.032343
157	O	-0.260494
	Tot	1.000037

Mo21 4r-1β

1	Mo	0.651906
2	N	-0.160309
3	C	0.013487
4	N	0.036042
5	C	0.079706
6	C	-0.045437
7	H	0.034667
8	C	0.032526
9	C	0.011083
10	C	-0.028727
11	H	0.045438
12	C	-0.014975
13	C	-0.028744
14	H	0.045202
15	C	0.012969
16	C	0.043424
17	C	0.02686
18	C	-0.03094
19	H	0.045273
20	C	-0.010422
21	H	0.051133
22	C	-0.030644
23	H	0.046406
24	C	0.026349
25	C	-0.013979
26	C	-0.078632
27	H	0.03083
28	C	-0.008004
29	C	0.037797
30	C	-0.114887
31	C	0.051072
32	C	-0.041087
33	C	0.0075

34	C	-0.060219
35	C	-0.069418
36	C	0.217077
37	C	0.217564
38	O	-0.119091
39	O	-0.252916
40	O	-0.118195
41	O	-0.246839
42	C	0.043119
43	C	-0.079038
44	C	0.049214
45	C	-0.079878
46	H	0.025537
47	H	0.016436
48	H	0.043303
49	H	0.040779
50	H	0.04329
51	H	0.049988
52	H	0.039653
53	H	0.045345
54	H	0.030324
55	H	0.035651
56	H	0.043986
57	H	0.036279
58	H	0.03178
59	H	0.038412
60	H	0.034873
61	H	0.04241
62	H	0.041745
63	C	-0.025006
64	C	0.015627
65	C	0.214574
66	C	0.215859
67	C	-0.025609
68	C	-0.040728
69	C	-0.091813
70	O	-0.240305
71	O	-0.107743
72	O	-0.242849
73	O	-0.105748
74	C	0.044218
75	C	-0.078144
76	C	0.048487
77	C	-0.080236
78	H	0.065849
79	H	0.048805

80	H	0.048687
81	H	0.084919
82	H	0.042984
83	H	0.038507
84	H	0.044987
85	H	0.035037
86	H	0.042104
87	H	0.035096
88	H	0.037359
89	H	0.038934
90	H	0.036344
91	H	0.034434
92	H	0.04244
93	H	0.035929
94	C	-0.018453
95	H	0.007357
96	C	-0.011279
97	H	0.016894
98	C	-0.076933
99	H	0.030829
100	H	0.029105
101	H	0.032204
102	C	-0.077254
103	H	0.02725
104	H	0.032543
105	H	0.031213
106	C	-0.012123
107	H	0.012585
108	C	-0.085586
109	H	0.013569
110	H	0.030418
111	H	0.027235
112	C	-0.081172
113	H	0.028046
114	H	0.024123
115	H	0.031836
116	H	0.009132
117	C	0.020497
118	H	0.051745
119	H	0.045343
120	C	-0.079245
121	H	0.010675
122	H	0.026469
123	H	0.031389
124	C	-0.081171
125	H	0.030456

126	H	0.025657
127	H	0.028588
128	C	-0.085254
129	H	0.027396
130	H	0.032211
131	H	0.012231
132	C	-0.076356
133	H	0.033391
134	H	0.03134
135	H	0.035375
136	C	-0.080262
137	H	0.046554
138	H	0.034307
139	H	0.036945
140	C	-0.082743
141	H	0.035683
142	H	0.025432
143	H	0.044253
144	C	-0.07502
145	H	0.042654
146	H	0.028932
147	H	0.030533
148	C	-0.084519
149	H	0.024811
150	H	0.036855
151	H	0.026371
152	O	-0.290422
153	S	0.51294
154	O	-0.299309
155	O	-0.286125
156	C	0.285875
157	F	-0.073358
158	F	-0.08425
159	F	-0.075233
	Tot	1.000237

Mo6 4r-1β

1	Mo	0.653205
2	N	-0.158418
3	C	0.021901
4	N	-0.011071
5	C	0.018544
6	H	0.044055
7	N	-0.011207
8	C	0.018685
9	H	0.047656

10	C	0.017361
11	C	0.01341
12	C	-0.030238
13	H	0.046018
14	C	0.025707
15	C	-0.044825
16	H	0.036826
17	C	-0.00357
18	C	0.020904
19	C	0.004343
20	C	-0.042182
21	H	0.040388
22	C	0.020328
23	C	-0.041436
24	H	0.04013
25	C	0.004714
26	C	-0.085078
27	H	0.021085
28	H	0.023966
29	H	0.043843
30	C	-0.072796
31	H	0.037625
32	H	0.044235
33	H	0.035878
34	C	-0.079748
35	H	0.037741
36	H	0.02501
37	H	0.040333
38	C	0.044346
39	C	-0.03069
40	C	0.020801
41	C	-0.029543
42	H	0.04155
43	C	0.021283
44	C	-0.033551
45	C	-0.085062
46	H	0.036652
47	C	-0.011206
48	C	0.02173
49	C	-0.111313
50	C	0.023417
51	C	-0.0458
52	C	0.008043
53	C	-0.059468
54	C	-0.074099
55	C	0.21806

56	C	0.216996
57	O	-0.118915
58	O	-0.24987
59	O	-0.119878
60	O	-0.252005
61	C	0.043972
62	C	-0.078927
63	C	0.048556
64	C	-0.08008
65	H	0.030515
66	H	0.013952
67	H	0.037896
68	H	0.035723
69	H	0.039415
70	H	0.042676
71	H	0.039957
72	H	0.045243
73	H	0.033284
74	H	0.033823
75	H	0.044197
76	H	0.03862
77	H	0.031235
78	H	0.035887
79	H	0.042165
80	H	0.03802
81	H	0.036928
82	C	-0.029696
83	C	0.017215
84	C	0.220657
85	C	0.217266
86	C	-0.021874
87	C	-0.036661
88	C	-0.09852
89	O	-0.245853
90	O	-0.100383
91	O	-0.251471
92	O	-0.107326
93	C	0.045357
94	C	-0.075199
95	C	0.049811
96	C	-0.080347
97	H	0.067989
98	H	0.054371
99	H	0.050831
100	H	0.088283
101	H	0.048657

102	H	0.030958
103	H	0.037446
104	H	0.044541
105	H	0.03404
106	H	0.034557
107	H	0.04252
108	H	0.031545
109	H	0.033932
110	H	0.038385
111	H	0.041824
112	H	0.036557
113	C	-0.072038
114	H	0.037743
115	H	0.03976
116	H	0.040388
117	C	-0.086073
118	H	0.016719
119	H	0.035044
120	H	0.040851
121	C	-0.084247
122	H	0.021755
123	H	0.042754
124	H	0.025503
125	O	-0.297428
126	S	0.515499
127	O	-0.293658
128	O	-0.298536
129	C	0.283889
130	F	-0.084087
131	F	-0.083149
132	F	-0.085016
133	H	0.042956
134	H	0.043644
135	C	-0.072036
136	H	0.03883
137	H	0.042552
138	H	0.039074
139	C	-0.076402
140	H	0.037953
141	H	0.040433
142	H	0.036324
143	H	0.053605
144	H	0.050395
	Tot	1.000267

Mo8 4r-1β

1	Mo	0.640724
2	N	-0.159665
3	C	-0.029991
4	N	0.019825
5	C	0.007898
6	H	0.072427
7	N	0.02122
8	C	0.005297
9	H	0.070468
10	C	0.042784
11	C	0.025053
12	C	-0.026
13	H	0.045652
14	C	-0.012241
15	H	0.052438
16	C	-0.021498
17	H	0.054158
18	C	-0.022818
19	C	0.028158
20	C	-0.084944
21	H	0.038332
22	C	-0.01541
23	C	0.026704
24	C	-0.108011
25	C	0.029621
26	C	-0.043916
27	C	0.008675
28	C	-0.0572
29	C	-0.071721
30	C	0.215077
31	C	0.215693
32	O	-0.119597
33	O	-0.257706
34	O	-0.120587
35	O	-0.25573
36	C	0.041444
37	C	-0.079452
38	C	0.048776
39	C	-0.07924
40	H	0.031861
41	H	0.01662
42	H	0.03973
43	H	0.038287
44	H	0.045325
45	H	0.037663
46	H	0.040006

47	H	0.044886
48	H	0.044473
49	H	0.034922
50	H	0.034569
51	H	0.034535
52	H	0.02781
53	H	0.041576
54	H	0.037879
55	H	0.035048
56	H	0.042747
57	C	-0.027664
58	C	0.018089
59	C	0.222051
60	C	0.218573
61	C	-0.021375
62	C	-0.037673
63	C	-0.09462
64	O	-0.242059
65	O	-0.101326
66	O	-0.251478
67	O	-0.103171
68	C	0.048638
69	C	-0.081989
70	C	0.050813
71	C	-0.080663
72	H	0.069568
73	H	0.054536
74	H	0.052646
75	H	0.090294
76	H	0.050194
77	H	0.032541
78	H	0.042832
79	H	0.036895
80	H	0.035647
81	H	0.026547
82	H	0.039994
83	H	0.046492
84	H	0.031491
85	H	0.034373
86	H	0.042045
87	H	0.028315
88	O	-0.287528
89	S	0.512824
90	O	-0.298975
91	O	-0.291936
92	C	0.280439

93	F	-0.078263
94	F	-0.089441
95	F	-0.090228
96	C	-0.047417
97	C	0.04296
98	C	-0.04985
99	C	-0.037366
100	C	-0.042101
101	C	-0.040424
102	H	0.028588
103	H	0.029375
104	H	0.023281
105	H	0.02495
106	H	0.027842
107	H	0.039501
108	H	0.029258
109	H	0.03483
110	H	0.028081
111	H	0.036903
112	H	0.024673
113	C	-0.041767
114	C	-0.038467
115	C	-0.049232
116	C	0.041016
117	C	-0.051578
118	C	-0.042177
119	H	0.035603
120	H	0.027807
121	H	0.038344
122	H	0.028297
123	H	0.023441
124	H	0.0281
125	H	0.028693
126	H	0.019105
127	H	0.026516
128	H	0.03553
129	H	0.021007
130	C	-0.083043
131	H	0.012157
132	H	0.036901
133	H	0.025508
134	C	-0.076908
135	H	0.036809
136	H	0.03205
137	H	0.030107
138	C	-0.08268

139	H	0.035457
140	H	0.014853
141	H	0.032277
142	H	0.043356
	Tot	1.000249

Mo9 4r-1β

1	Mo	0.652981
2	N	-0.168295
3	C	-0.013212
4	N	0.017494
5	C	0.000988
6	H	0.068467
7	N	0.019369
8	C	0.000566
9	H	0.06931
10	C	0.036515
11	C	0.021223
12	C	-0.030773
13	H	0.045024
14	C	-0.019614
15	H	0.049892
16	C	-0.034874
17	H	0.044463
18	C	-0.034407
19	C	0.028333
20	C	-0.08389
21	H	0.039707
22	C	-0.012468
23	C	0.021187
24	C	-0.119821
25	C	0.025345
26	C	-0.042365
27	C	0.00898
28	C	-0.053982
29	C	-0.072781
30	C	0.229901
31	C	0.209795
32	O	-0.106025
33	O	-0.236781
34	O	-0.119257
35	O	-0.265628
36	C	0.048478
37	C	-0.078249
38	C	0.048284
39	C	-0.079714

40	H	0.034188
41	H	0.019838
42	H	0.03759
43	H	0.039324
44	H	0.042869
45	H	0.040936
46	H	0.03967
47	H	0.04357
48	H	0.043775
49	H	0.03509
50	H	0.033281
51	H	0.048274
52	H	0.032421
53	H	0.044309
54	H	0.032772
55	H	0.039286
56	H	0.041154
57	C	-0.030936
58	C	0.013596
59	C	0.220132
60	C	0.215693
61	C	-0.023026
62	C	-0.038214
63	C	-0.097536
64	O	-0.237113
65	O	-0.106898
66	O	-0.256286
67	O	-0.10904
68	C	0.049694
69	C	-0.083223
70	C	0.042616
71	C	-0.078868
72	H	0.064741
73	H	0.053996
74	H	0.052352
75	H	0.090288
76	H	0.0487
77	H	0.030979
78	H	0.039224
79	H	0.029675
80	H	0.036607
81	H	0.03054
82	H	0.045763
83	H	0.048338
84	H	0.024606
85	H	0.034628

86	H	0.044627
87	H	0.029359
88	O	-0.268501
89	C	-0.044088
90	C	0.041785
91	C	-0.052227
92	C	-0.040532
93	C	-0.0425
94	C	-0.044483
95	H	0.029756
96	H	0.027986
97	H	0.029627
98	H	0.020278
99	H	0.027437
100	H	0.035995
101	H	0.025157
102	H	0.032314
103	H	0.026373
104	H	0.031957
105	H	0.020316
106	C	-0.041627
107	C	-0.04132
108	C	-0.047282
109	C	0.045422
110	C	-0.047581
111	C	-0.03951
112	H	0.035881
113	H	0.027203
114	H	0.036906
115	H	0.023691
116	H	0.028383
117	H	0.02818
118	H	0.026589
119	H	0.030481
120	H	0.028861
121	H	0.036594
122	H	0.028009
123	C	-0.085326
124	H	0.009995
125	H	0.03206
126	H	0.027854
127	C	-0.077308
128	H	0.034561
129	H	0.033488
130	H	0.029834
131	C	-0.086029

132	H	0.031913
133	H	0.010897
134	H	0.033339
135	H	0.031468
136	C	0.052147
137	C	0.073892
138	C	0.063027
139	C	0.079889
140	C	0.075173
141	C	0.070673
142	F	-0.083275
143	F	-0.082467
144	F	-0.087595
145	F	-0.073776
146	F	-0.077255
	Tot	1.000238

Mo7 4r-1β

1	Mo	0.650404
2	N	-0.175458
3	C	0.021451
4	N	-0.014736
5	C	0.014554
6	H	0.04365
7	N	-0.011396
8	C	0.01502
9	H	0.044861
10	C	0.022115
11	C	0.002544
12	C	-0.040613
13	H	0.042807
14	C	0.021811
15	C	-0.03973
16	H	0.039484
17	C	0.001863
18	C	0.021806
19	C	0.003084
20	C	-0.039613
21	H	0.040689
22	C	0.020169
23	C	-0.040231
24	H	0.040808
25	C	0.005476
26	C	-0.083647
27	H	0.018091
28	H	0.026668

29	H	0.044219
30	C	-0.073537
31	H	0.038072
32	H	0.042239
33	H	0.034839
34	C	-0.078964
35	H	0.039365
36	H	0.024385
37	H	0.040596
38	C	0.03608
39	C	0.020905
40	C	-0.033989
41	H	0.043169
42	C	-0.021638
43	H	0.047457
44	C	-0.034087
45	H	0.045373
46	C	0.016719
47	C	-0.080076
48	H	0.038267
49	H	0.03952
50	H	0.025349
51	C	-0.079539
52	H	0.023394
53	H	0.034782
54	H	0.041041
55	C	-0.08695
56	H	0.036829
57	C	-0.010755
58	C	0.011637
59	C	-0.117439
60	C	0.021438
61	C	-0.046002
62	C	0.007164
63	C	-0.059353
64	C	-0.073755
65	C	0.21606
66	C	0.217216
67	O	-0.119097
68	O	-0.258706
69	O	-0.120393
70	O	-0.249331
71	C	0.043047
72	C	-0.079035
73	C	0.048531
74	C	-0.081538

75	H	0.030445
76	H	0.01515
77	H	0.03723
78	H	0.035148
79	H	0.037572
80	H	0.042776
81	H	0.039824
82	H	0.045077
83	H	0.029385
84	H	0.032851
85	H	0.043289
86	H	0.037337
87	H	0.030436
88	H	0.035127
89	H	0.041349
90	H	0.037745
91	H	0.037361
92	C	-0.031206
93	C	0.01503
94	C	0.219853
95	C	0.215059
96	C	-0.020407
97	C	-0.03467
98	C	-0.09487
99	O	-0.241676
100	O	-0.105231
101	O	-0.25552
102	O	-0.109096
103	C	0.045843
104	C	-0.079066
105	C	0.046095
106	C	-0.078925
107	H	0.06287
108	H	0.055169
109	H	0.050102
110	H	0.08771
111	H	0.045434
112	H	0.030546
113	H	0.033693
114	H	0.04386
115	H	0.035933
116	H	0.035748
117	H	0.044324
118	H	0.029939
119	H	0.041736
120	H	0.030282

121	H	0.04073
122	H	0.036711
123	C	-0.071375
124	H	0.040872
125	H	0.039029
126	H	0.041884
127	C	-0.078682
128	H	0.026441
129	H	0.041529
130	H	0.042744
131	C	-0.081625
132	H	0.019829
133	H	0.042346
134	H	0.028793
135	O	-0.297984
136	H	0.051445
137	H	0.051098
138	C	0.022458
139	H	0.0325
140	C	0.2928
141	F	-0.093736
142	F	-0.109301
143	F	-0.081917
144	C	0.290384
145	F	-0.090919
146	F	-0.100762
147	F	-0.087382
	Tot	1.000015

Mo22 4r-1β

1	Mo	0.602642
2	N	-0.1843
3	C	0.00868
4	N	0.028008
5	C	0.079998
6	C	-0.045022
7	H	0.035093
8	C	0.034508
9	C	0.011566
10	C	-0.031525
11	H	0.044896
12	C	-0.022634
13	C	-0.03232
14	H	0.044024
15	C	0.011767
16	C	0.05264

17	C	-0.016644
18	C	-0.018241
19	H	0.051404
20	C	-0.009016
21	H	0.056222
22	C	-0.009502
23	H	0.055766
24	C	-0.016323
25	C	-0.084246
26	H	0.024117
27	C	-0.010977
28	C	0.026671
29	C	-0.117662
30	C	0.038679
31	C	-0.04462
32	C	0.00683
33	C	-0.062627
34	C	-0.071819
35	C	0.214641
36	C	0.217731
37	O	-0.122368
38	O	-0.258493
39	O	-0.119294
40	O	-0.250945
41	C	0.044165
42	C	-0.078919
43	C	0.049099
44	C	-0.081233
45	H	0.025786
46	H	0.010842
47	H	0.040468
48	H	0.037792
49	H	0.041986
50	H	0.045038
51	H	0.040217
52	H	0.045638
53	H	0.02954
54	H	0.033668
55	H	0.043785
56	H	0.040589
57	H	0.030673
58	H	0.037957
59	H	0.035599
60	H	0.042342
61	H	0.038865
62	C	-0.024241

63	C	0.01654
64	C	0.215195
65	C	0.21899
66	C	-0.025477
67	C	-0.039359
68	C	-0.090668
69	O	-0.231458
70	O	-0.111712
71	O	-0.252139
72	O	-0.105147
73	C	0.046847
74	C	-0.079433
75	C	0.048801
76	C	-0.078424
77	H	0.067636
78	H	0.050959
79	H	0.049019
80	H	0.087966
81	H	0.048708
82	H	0.039011
83	H	0.04678
84	H	0.036387
85	H	0.04409
86	H	0.037308
87	H	0.035971
88	H	0.044998
89	H	0.0311
90	H	0.035918
91	H	0.043619
92	H	0.035865
93	C	-0.018711
94	H	0.006811
95	C	-0.011149
96	H	0.016723
97	Br	-0.166863
98	C	0.020478
99	H	0.049723
100	H	0.044035
101	C	-0.084193
102	H	0.015872
103	H	0.031032
104	H	0.026584
105	C	-0.076141
106	H	0.030627
107	H	0.036367
108	H	0.026997

109	C	-0.085997
110	H	0.024513
111	H	0.034479
112	H	0.011098
113	C	-0.076534
114	H	0.032923
115	H	0.030399
116	H	0.03538
117	C	-0.080969
118	H	0.046063
119	H	0.034045
120	H	0.036206
121	C	-0.083953
122	H	0.035281
123	H	0.024415
124	H	0.041929
125	C	-0.074285
126	H	0.043166
127	H	0.030918
128	H	0.0316
129	C	-0.084626
130	H	0.027029
131	H	0.036032
132	H	0.026856
133	C	0.301098
134	F	-0.094578
135	F	-0.105578
136	F	-0.098714
137	H	0.038879
	Tot	1.000118

Mo1 4r-2α

1	Mo	0.657476
2	N	-0.156145
3	C	-0.018304
4	N	0.021427
5	C	0.009785
6	H	0.074741
7	N	0.022425
8	C	0.008535
9	H	0.074558
10	C	0.017562
11	C	0.015352
12	C	-0.031283
13	H	0.043175
14	C	0.025964

15	C	-0.044738
16	H	0.039503
17	C	0.000428
18	C	0.021279
19	C	0.014304
20	C	-0.036722
21	H	0.044204
22	C	0.022473
23	C	-0.04212
24	H	0.038368
25	C	0.005495
26	C	-0.085005
27	H	0.014954
28	H	0.035552
29	H	0.044178
30	C	-0.072255
31	H	0.039882
32	H	0.044595
33	H	0.03587
34	C	-0.084745
35	H	0.037123
36	H	0.035869
37	H	0.020745
38	C	0.037803
39	C	0.030368
40	C	-0.030618
41	H	0.046393
42	C	-0.015125
43	H	0.051573
44	C	-0.035405
45	H	0.044906
46	C	0.021289
47	C	-0.019383
48	H	0.025967
49	C	-0.013219
50	C	-0.05076
51	C	-0.055906
52	C	0.035057
53	C	-0.05104
54	C	0.009472
55	C	-0.056175
56	C	-0.073869
57	C	0.224621
58	C	0.21836
59	O	-0.105858
60	O	-0.233499

61	O	-0.11399
62	O	-0.253311
63	C	0.04946
64	C	-0.079346
65	C	0.048432
66	C	-0.078874
67	H	0.032012
68	H	0.022186
69	H	0.035421
70	H	0.037665
71	H	0.046195
72	H	0.040627
73	H	0.039351
74	H	0.044804
75	H	0.035568
76	H	0.034867
77	H	0.044002
78	H	0.037566
79	H	0.0461
80	H	0.036184
81	H	0.034864
82	H	0.043648
83	H	0.036307
84	C	-0.043591
85	C	0.009829
86	C	0.216565
87	C	0.215036
88	C	-0.02448
89	C	0.00486
90	C	-0.092359
91	O	-0.24639
92	O	-0.11544
93	O	-0.248361
94	O	-0.117567
95	C	0.044659
96	C	-0.078521
97	C	0.050409
98	C	-0.07796
99	H	0.033532
100	H	0.04786
101	H	0.050562
102	H	0.078562
103	H	0.031844
104	H	0.04122
105	H	0.041976
106	H	0.045888

107	H	0.035691
108	H	0.046212
109	H	0.036393
110	H	0.042337
111	H	0.027691
112	H	0.035778
113	H	0.044227
114	H	0.037135
115	C	-0.075049
116	H	0.043183
117	H	0.035616
118	H	0.038245
119	C	-0.090018
120	H	0.014214
121	H	0.035378
122	C	-0.083528
123	H	0.042949
124	H	0.031824
125	H	0.017288
126	S	0.500111
127	O	-0.302318
128	O	-0.30882
129	C	0.287038
130	F	-0.083945
131	F	-0.085379
132	F	-0.081698
133	O	-0.317659
134	H	0.033419
135	C	-0.079058
136	H	0.02343
137	H	0.042666
138	H	0.039171
139	C	-0.077312
140	H	0.041871
141	H	0.027964
142	H	0.03742
	Tot	0.999793

Mo2 4r-2α

1	Mo	0.666512
2	N	-0.151912
3	C	-0.017217
4	N	0.018962
5	C	0.008346
6	H	0.074563
7	N	0.022648

8	C	0.008039
9	H	0.07396
10	C	0.016891
11	C	0.016308
12	C	-0.031007
13	H	0.043906
14	C	0.026297
15	C	-0.043607
16	H	0.040048
17	C	0.001416
18	C	0.025446
19	C	0.017319
20	C	-0.035142
21	H	0.044872
22	C	0.023593
23	C	-0.04205
24	H	0.038568
25	C	0.008472
26	C	-0.083639
27	H	0.020685
28	H	0.031518
29	H	0.043636
30	C	-0.071707
31	H	0.040856
32	H	0.043002
33	H	0.038516
34	C	-0.087135
35	H	0.031177
36	H	0.038024
37	H	0.018443
38	C	0.047176
39	C	0.031338
40	C	-0.027194
41	H	0.045487
42	C	-0.015323
43	H	0.051618
44	C	-0.026342
45	H	0.050689
46	C	-0.022783
47	C	-0.018874
48	H	0.027341
49	C	-0.012577
50	C	-0.051889
51	C	-0.052181
52	C	0.034946
53	C	-0.050506

54	C	0.009089
55	C	-0.056645
56	C	-0.073802
57	C	0.224366
58	C	0.21811
59	O	-0.106266
60	O	-0.234775
61	O	-0.114521
62	O	-0.254911
63	C	0.049689
64	C	-0.079467
65	C	0.048705
66	C	-0.07905
67	H	0.032659
68	H	0.02224
69	H	0.035527
70	H	0.037723
71	H	0.044987
72	H	0.041567
73	H	0.039516
74	H	0.045051
75	H	0.035444
76	H	0.034733
77	H	0.044067
78	H	0.037643
79	H	0.046275
80	H	0.036208
81	H	0.034824
82	H	0.043662
83	H	0.037113
84	C	-0.048535
85	C	0.010661
86	C	0.218766
87	C	0.213082
88	C	-0.023986
89	C	0.007536
90	C	-0.090837
91	O	-0.234469
92	O	-0.11828
93	O	-0.249121
94	O	-0.114899
95	C	0.043999
96	C	-0.078813
97	C	0.051138
98	C	-0.078019
99	H	0.033853

100	H	0.047486
101	H	0.050666
102	H	0.079907
103	H	0.027441
104	H	0.039095
105	H	0.042218
106	H	0.047264
107	H	0.036146
108	H	0.046384
109	H	0.035713
110	H	0.039319
111	H	0.030543
112	H	0.035914
113	H	0.043907
114	H	0.037526
115	C	-0.074367
116	H	0.043807
117	H	0.036478
118	H	0.039365
119	C	-0.090651
120	H	0.013567
121	H	0.035842
122	C	-0.082868
123	H	0.042381
124	H	0.034804
125	H	0.015695
126	S	0.500738
127	O	-0.305293
128	O	-0.309533
129	C	0.287247
130	F	-0.083313
131	F	-0.084633
132	F	-0.081244
133	O	-0.313418
134	H	0.033109
135	C	0.02818
136	C	-0.092527
137	H	0.001062
138	H	0.034519
139	H	0.01215
140	C	-0.077609
141	H	0.035875
142	H	0.030634
143	H	0.026386
144	C	-0.084479
145	H	0.032103

146	H	0.013498
147	H	0.029686
148	H	0.041705
	Tot	0.9998

Mo3 4r-2α

1	Mo	0.664554
2	N	-0.17753
3	C	-0.020043
4	N	0.02158
5	C	0.008982
6	H	0.074902
7	N	0.022913
8	C	0.008965
9	H	0.074695
10	C	0.015792
11	C	0.014117
12	C	-0.033457
13	H	0.042052
14	C	0.025441
15	C	-0.044558
16	H	0.039935
17	C	0.002153
18	C	0.017569
19	C	0.01135
20	C	-0.041409
21	H	0.044309
22	C	0.016662
23	C	-0.049856
24	H	0.035258
25	C	0.000527
26	C	-0.085602
27	H	0.015109
28	H	0.034744
29	H	0.043267
30	C	-0.073362
31	H	0.035471
32	H	0.04251
33	H	0.038377
34	C	-0.088377
35	H	0.034001
36	H	0.035972
37	H	0.016293
38	C	0.034131
39	C	0.045239
40	C	-0.027682

41	H	0.05684
42	C	-0.006261
43	H	0.059888
44	C	-0.032009
45	H	0.056234
46	C	0.040273
47	C	-0.019557
48	H	0.026414
49	C	-0.013442
50	C	-0.049442
51	C	-0.052084
52	C	0.035374
53	C	-0.051459
54	C	0.009032
55	C	-0.056019
56	C	-0.074662
57	C	0.224052
58	C	0.218225
59	O	-0.105858
60	O	-0.236437
61	O	-0.114765
62	O	-0.253974
63	C	0.049656
64	C	-0.079185
65	C	0.048867
66	C	-0.078839
67	H	0.032558
68	H	0.02312
69	H	0.035261
70	H	0.037806
71	H	0.045221
72	H	0.04169
73	H	0.03976
74	H	0.045151
75	H	0.035501
76	H	0.034716
77	H	0.044224
78	H	0.03744
79	H	0.045925
80	H	0.036431
81	H	0.035332
82	H	0.043655
83	H	0.035846
84	C	-0.046052
85	C	0.009698
86	C	0.216061

87	C	0.213534
88	C	-0.022939
89	C	0.006855
90	C	-0.091674
91	O	-0.255057
92	O	-0.117406
93	O	-0.248904
94	O	-0.118408
95	C	0.041398
96	C	-0.078132
97	C	0.05016
98	C	-0.078437
99	H	0.034657
100	H	0.048345
101	H	0.051878
102	H	0.076913
103	H	0.025029
104	H	0.04103
105	H	0.042189
106	H	0.045825
107	H	0.035267
108	H	0.045982
109	H	0.035866
110	H	0.040696
111	H	0.024276
112	H	0.036708
113	H	0.043439
114	H	0.037376
115	C	-0.07466
116	H	0.043473
117	H	0.03601
118	H	0.038845
119	C	-0.08865
120	H	0.016054
121	H	0.034766
122	C	-0.087565
123	H	0.040463
124	H	0.032628
125	H	0.009348
126	S	0.503875
127	O	-0.300766
128	O	-0.305374
129	C	0.287851
130	F	-0.085613
131	F	-0.084183
132	F	-0.082278

133	Cl	0.036378
134	Cl	0.03584
135	O	-0.313023
136	H	0.034794
	Tot	0.999883

Mo10 4r-2α

1	Mo	0.646683
2	N	-0.15791
3	C	-0.025453
4	N	0.0093
5	C	0.108409
6	N	0.064104
7	C	0.041552
8	C	-0.018923
9	C	-0.003558
10	H	0.056694
11	C	-0.013682
12	C	-0.022442
13	H	0.050302
14	C	-0.046545
15	C	0.037742
16	C	-0.018384
17	C	-0.015881
18	H	0.053731
19	C	-0.018687
20	C	-0.0258
21	H	0.043489
22	C	-0.039288
23	C	0.034174
24	C	0.030334
25	C	-0.029478
26	H	0.049237
27	C	-0.013244
28	H	0.053624
29	C	-0.034508
30	H	0.047289
31	C	0.017739
32	C	-0.0142
33	H	0.031753
34	C	-0.01238
35	C	-0.045249
36	C	-0.055401
37	C	0.041453
38	C	-0.050326
39	C	0.008865

40	C	-0.054757
41	C	-0.072642
42	C	0.2217
43	C	0.215331
44	O	-0.108908
45	O	-0.240385
46	O	-0.112863
47	O	-0.266962
48	C	0.046247
49	C	-0.079627
50	C	0.048481
51	C	-0.078906
52	H	0.033344
53	H	0.021061
54	H	0.036934
55	H	0.039336
56	H	0.047056
57	H	0.04335
58	H	0.039046
59	H	0.044992
60	H	0.035551
61	H	0.034755
62	H	0.043994
63	H	0.030175
64	H	0.045741
65	H	0.036434
66	H	0.034686
67	H	0.042714
68	H	0.037653
69	C	-0.048224
70	C	0.010263
71	C	0.219019
72	C	0.213983
73	C	-0.024595
74	C	0.00923
75	C	-0.0945
76	O	-0.238292
77	O	-0.116754
78	O	-0.247609
79	O	-0.115654
80	C	0.04268
81	C	-0.079083
82	C	0.051127
83	C	-0.077835
84	H	0.034541
85	H	0.048609

86	H	0.050471
87	H	0.079502
88	H	0.029485
89	H	0.039864
90	H	0.042446
91	H	0.046912
92	H	0.036117
93	H	0.046724
94	H	0.03631
95	H	0.040155
96	H	0.023952
97	H	0.035882
98	H	0.044041
99	H	0.036911
100	S	0.493173
101	O	-0.314543
102	O	-0.309995
103	C	0.284306
104	F	-0.08162
105	F	-0.087436
106	F	-0.093797
107	O	-0.326864
108	C	-0.083531
109	H	0.027615
110	H	0.035322
111	H	0.040706
112	C	-0.074542
113	H	0.033394
114	H	0.036668
115	H	0.045538
116	N	-0.110541
117	H	0.050471
118	H	0.051017
119	H	0.032453
120	H	0.033407
121	H	0.045593
122	H	0.055563
123	C	-0.00967
124	C	-0.030487
125	C	-0.021207
126	C	-0.024286
127	C	-0.022574
128	C	-0.015451
129	H	0.037798
130	H	0.046611
131	H	0.049907

132	H	0.050816
133	H	0.051747
	Tot	0.999908

Mo13 4r-2α

1	Mo	0.660577
2	N	-0.190632
3	C	0.023289
4	N	-0.011049
5	C	0.01517
6	N	-0.012705
7	C	0.014374
8	C	0.013507
9	C	0.008337
10	C	-0.036206
11	H	0.040059
12	C	0.027219
13	C	-0.043283
14	H	0.040998
15	C	0.002341
16	C	0.019744
17	C	0.006695
18	C	-0.041859
19	H	0.042222
20	C	0.018381
21	C	-0.043238
22	H	0.037492
23	C	0.006175
24	C	-0.087876
25	H	0.016508
26	H	0.034709
27	H	0.036846
28	C	-0.072464
29	H	0.039803
30	H	0.044173
31	H	0.037622
32	C	-0.086824
33	H	0.030846
34	H	0.039322
35	H	0.018164
36	C	0.048892
37	C	-0.011803
38	C	-0.008641
39	H	0.055339
40	C	-0.013418
41	H	0.054962

42	C	-0.021668
43	H	0.049898
44	C	-0.024074
45	C	-0.035726
46	H	0.024102
47	C	-0.017341
48	C	-0.05102
49	C	-0.039351
50	C	0.013624
51	C	-0.051119
52	C	0.011227
53	C	-0.05719
54	C	-0.079787
55	C	0.22094
56	C	0.218878
57	O	-0.104316
58	O	-0.245726
59	O	-0.118342
60	O	-0.253083
61	C	0.047843
62	C	-0.079765
63	C	0.047861
64	C	-0.079679
65	H	0.031963
66	H	0.023391
67	H	0.03281
68	H	0.034928
69	H	0.033121
70	H	0.04258
71	H	0.039492
72	H	0.043545
73	H	0.035337
74	H	0.033807
75	H	0.043107
76	H	0.035603
77	H	0.044297
78	H	0.035564
79	H	0.034769
80	H	0.041907
81	H	0.028777
82	C	-0.051093
83	C	0.010362
84	C	0.217784
85	C	0.212084
86	C	-0.028356
87	C	0.003689

88	C	-0.109167
89	O	-0.255069
90	O	-0.116021
91	O	-0.247294
92	O	-0.117953
93	C	0.041463
94	C	-0.07914
95	C	0.049779
96	C	-0.078432
97	H	0.037965
98	H	0.043979
99	H	0.046541
100	H	0.066607
101	H	0.02223
102	H	0.038608
103	H	0.041728
104	H	0.045134
105	H	0.034863
106	H	0.045329
107	H	0.036218
108	H	0.041519
109	H	0.02258
110	H	0.035949
111	H	0.042408
112	H	0.036259
113	C	-0.076592
114	H	0.040659
115	H	0.038904
116	H	0.034406
117	C	-0.084747
118	H	0.021698
119	H	0.031802
120	C	-0.090727
121	H	0.040553
122	H	0.031902
123	H	0.00564
124	O	-0.252071
125	H	0.039648
126	H	0.043589
127	C	0.30261
128	F	-0.091855
129	F	-0.088865
130	F	-0.094198
131	C	0.054573
132	C	0.076006
133	C	0.07036

134	C	0.078128
135	C	0.078282
136	C	0.078629
137	F	-0.055706
138	F	-0.076766
139	F	-0.081407
140	F	-0.085638
141	F	-0.078394
142	C	-0.040877
143	H	0.043528
144	H	0.050637
145	H	0.037736
146	H	0.053306
147	H	0.048453
148	H	0.041479
	Tot	1.00019

Mo14 4r-2α

1	Mo	0.652797
2	N	-0.176392
3	C	0.020274
4	N	-0.01378
5	C	0.013814
6	N	-0.013153
7	C	0.013395
8	C	0.015274
9	C	0.01001
10	C	-0.035243
11	H	0.040039
12	C	0.026958
13	C	-0.0441
14	H	0.040442
15	C	0.00117
16	C	0.019535
17	C	0.008041
18	C	-0.04046
19	H	0.042579
20	C	0.02236
21	C	-0.0391
22	H	0.039437
23	C	0.007851
24	C	-0.088856
25	H	0.016249
26	H	0.034034
27	H	0.036826
28	C	-0.071525

29	H	0.039703
30	H	0.04219
31	H	0.039518
32	C	-0.084186
33	H	0.031669
34	H	0.037992
35	H	0.02227
36	C	0.035413
37	C	0.027964
38	C	-0.032418
39	H	0.045535
40	C	-0.017946
41	H	0.050851
42	C	-0.036115
43	H	0.043923
44	C	0.018376
45	C	-0.03588
46	H	0.023326
47	C	-0.017273
48	C	-0.056021
49	C	-0.041713
50	C	0.015111
51	C	-0.050529
52	C	0.010648
53	C	-0.056236
54	C	-0.079506
55	C	0.22059
56	C	0.219333
57	O	-0.102391
58	O	-0.249309
59	O	-0.119402
60	O	-0.253464
61	C	0.047409
62	C	-0.07954
63	C	0.048091
64	C	-0.079727
65	H	0.031857
66	H	0.023321
67	H	0.033144
68	H	0.035444
69	H	0.034285
70	H	0.042023
71	H	0.039628
72	H	0.043537
73	H	0.03523
74	H	0.03394

75	H	0.043097
76	H	0.035204
77	H	0.043521
78	H	0.035614
79	H	0.034849
80	H	0.041642
81	H	0.028964
82	C	-0.049477
83	C	0.010173
84	C	0.218827
85	C	0.214004
86	C	-0.027318
87	C	-0.00008
88	C	-0.10289
89	O	-0.241086
90	O	-0.116557
91	O	-0.246582
92	O	-0.115592
93	C	0.042224
94	C	-0.079423
95	C	0.050122
96	C	-0.078495
97	H	0.036036
98	H	0.044233
99	H	0.047088
100	H	0.067997
101	H	0.025002
102	H	0.038258
103	H	0.041707
104	H	0.045667
105	H	0.035297
106	H	0.04565
107	H	0.036355
108	H	0.039211
109	H	0.026048
110	H	0.034984
111	H	0.043308
112	H	0.036316
113	C	-0.076951
114	H	0.040093
115	H	0.038916
116	H	0.034358
117	C	-0.085901
118	H	0.021478
119	H	0.031328
120	C	-0.082438

121	H	0.04262
122	H	0.02924
123	H	0.023622
124	O	-0.258556
125	H	0.038835
126	C	0.052737
127	C	0.073181
128	C	0.066671
129	C	0.076957
130	C	0.076997
131	C	0.07531
132	F	-0.058189
133	F	-0.077303
134	F	-0.084179
135	F	-0.086742
136	F	-0.079074
137	C	-0.041611
138	H	0.040371
139	H	0.050226
140	H	0.038324
141	H	0.053012
142	H	0.048226
143	H	0.039803
144	C	-0.077534
145	H	0.041117
146	H	0.036413
147	H	0.029871
148	C	-0.085604
149	H	0.020593
150	H	0.035282
151	H	0.035198
	Tot	0.999737

Mo15 4r-2α

1	Mo	0.666434
2	N	-0.197508
3	C	0.024099
4	N	-0.010784
5	C	0.0142
6	N	-0.014695
7	C	0.014025
8	C	0.017655
9	C	0.013861
10	C	-0.03362
11	H	0.042779
12	C	0.025656

13	C	-0.041523
14	H	0.041865
15	C	0.004227
16	C	0.01945
17	C	0.006298
18	C	-0.041237
19	H	0.042914
20	C	0.015675
21	C	-0.052451
22	H	0.03555
23	C	-0.001217
24	C	-0.087852
25	H	0.017011
26	H	0.024358
27	H	0.042289
28	C	-0.076096
29	H	0.031876
30	H	0.041735
31	H	0.039094
32	C	-0.085631
33	H	0.039095
34	H	0.031978
35	H	0.02342
36	C	0.018377
37	C	0.102036
38	C	0.089919
39	C	0.099107
40	C	0.083885
41	C	0.093399
42	C	-0.030731
43	H	0.028074
44	C	-0.011132
45	C	-0.052485
46	C	-0.041301
47	C	0.019277
48	C	-0.050924
49	C	0.008903
50	C	-0.053937
51	C	-0.074151
52	C	0.22636
53	C	0.217127
54	O	-0.109837
55	O	-0.233899
56	O	-0.110705
57	O	-0.258502
58	C	0.048436

59	C	-0.078261
60	C	0.04874
61	C	-0.078861
62	H	0.034354
63	H	0.023724
64	H	0.033573
65	H	0.036513
66	H	0.04152
67	H	0.043139
68	H	0.039163
69	H	0.045292
70	H	0.035559
71	H	0.034808
72	H	0.044237
73	H	0.036838
74	H	0.046278
75	H	0.037417
76	H	0.035701
77	H	0.044154
78	H	0.034659
79	C	-0.050639
80	C	0.009488
81	C	0.216709
82	C	0.211702
83	C	-0.028074
84	C	-0.002685
85	C	-0.108129
86	O	-0.25736
87	O	-0.11627
88	O	-0.248427
89	O	-0.11792
90	C	0.040961
91	C	-0.076076
92	C	0.05005
93	C	-0.07898
94	H	0.032911
95	H	0.041917
96	H	0.047802
97	H	0.068481
98	H	0.022243
99	H	0.036974
100	H	0.04178
101	H	0.046624
102	H	0.035019
103	H	0.045621
104	H	0.034923

105	H	0.036777
106	H	0.027287
107	H	0.038453
108	H	0.045546
109	H	0.037077
110	C	-0.075306
111	H	0.043213
112	H	0.037663
113	H	0.036476
114	C	-0.082786
115	H	0.016509
116	H	0.038258
117	C	-0.087736
118	H	0.042063
119	H	0.032346
120	H	0.010829
121	O	-0.287054
122	H	0.042766
123	C	-0.042123
124	H	0.04004
125	H	0.048931
126	H	0.035584
127	H	0.052575
128	H	0.047218
129	H	0.038596
130	C	0.059994
131	C	-0.108135
132	H	0.032154
133	H	0.021617
134	H	0.033452
135	C	0.296774
136	F	-0.08031
137	F	-0.089931
138	F	-0.091922
139	C	0.289442
140	F	-0.091801
141	F	-0.098965
142	F	-0.088702
143	F	-0.060246
144	F	-0.068475
145	F	-0.069237
146	F	-0.075248
147	F	-0.061392
	Tot	0.999692

Mo11 4r-2α

1	Mo	0.657754
2	N	-0.185243
3	C	-0.016773
4	N	0.015248
5	C	0.00284
6	H	0.068115
7	N	0.016164
8	C	0.003643
9	H	0.069007
10	C	0.031228
11	C	0.018737
12	C	-0.034764
13	H	0.044936
14	C	-0.024164
15	H	0.048939
16	C	-0.038233
17	H	0.043695
18	C	0.011676
19	C	-0.022092
20	H	0.0368
21	C	-0.010525
22	C	-0.053353
23	C	-0.037175
24	C	0.020623
25	C	-0.047496
26	C	0.01117
27	C	-0.053334
28	C	-0.073287
29	C	0.217284
30	C	0.220717
31	O	-0.107297
32	O	-0.261092
33	O	-0.119669
34	O	-0.252052
35	C	0.04855
36	C	-0.078823
37	C	0.048476
38	C	-0.079767
39	H	0.032788
40	H	0.026779
41	H	0.033752
42	H	0.036886
43	H	0.042239
44	H	0.042025
45	H	0.04052
46	H	0.043736

47	H	0.035273
48	H	0.033809
49	H	0.043457
50	H	0.037208
51	H	0.04394
52	H	0.035677
53	H	0.035829
54	H	0.041961
55	H	0.033249
56	C	-0.049828
57	C	0.010047
58	C	0.218256
59	C	0.216063
60	C	-0.028047
61	C	-0.009148
62	C	-0.100192
63	O	-0.241161
64	O	-0.115616
65	O	-0.247203
66	O	-0.118296
67	C	0.041921
68	C	-0.079752
69	C	0.049891
70	C	-0.079049
71	H	0.042224
72	H	0.040929
73	H	0.044569
74	H	0.050572
75	H	0.024349
76	H	0.036554
77	H	0.04177
78	H	0.04566
79	H	0.034243
80	H	0.045124
81	H	0.035873
82	H	0.038891
83	H	0.025018
84	H	0.035015
85	H	0.04255
86	H	0.035894
87	O	-0.258263
88	C	-0.082226
89	H	0.022559
90	H	0.036547
91	H	0.037718
92	C	-0.080121

93	H	0.031093
94	H	0.027752
95	H	0.040536
96	C	0.044939
97	H	0.028031
98	C	0.050622
99	H	0.028596
100	C	-0.081144
101	H	0.042255
102	H	0.027779
103	H	0.035148
104	C	-0.080193
105	H	0.033644
106	H	0.029045
107	H	0.042786
108	C	-0.076768
109	H	0.046229
110	H	0.035405
111	H	0.034381
112	C	-0.081965
113	H	0.025473
114	H	0.051226
115	H	0.02616
116	C	0.073953
117	C	-0.011433
118	C	-0.011992
119	C	-0.032868
120	C	-0.033733
121	C	-0.043801
122	H	0.041246
123	H	0.042116
124	H	0.04406
125	C	-0.007664
126	C	0.007684
127	C	0.012696
128	C	-0.049401
129	C	-0.049472
130	C	0.012524
131	H	0.035777
132	H	0.03383
133	C	-0.013338
134	C	0.003235
135	C	0.010851
136	C	-0.054028
137	C	-0.049872
138	C	0.008766

139	H	0.031903
140	H	0.029391
141	C	-0.084908
142	H	0.029756
143	H	0.035486
144	H	0.02649
145	C	-0.081915
146	H	0.027653
147	H	0.039165
148	H	0.027282
149	C	-0.074478
150	H	0.038226
151	H	0.038331
152	H	0.03606
153	C	-0.088756
154	H	0.01755
155	H	0.024895
156	H	0.036128
157	C	-0.089904
158	H	0.025808
159	H	0.016692
160	H	0.031389
161	C	-0.075593
162	H	0.031261
163	H	0.040766
164	H	0.038162
	Tot	0.999937

Mo4 4r-2α

1	Mo	0.651311
2	N	-0.191513
3	C	-0.015518
4	N	0.018927
5	C	0.004952
6	H	0.073316
7	N	0.019222
8	C	0.006248
9	H	0.073405
10	C	0.019681
11	C	0.013337
12	C	-0.030661
13	H	0.044343
14	C	0.030253
15	C	-0.040019
16	H	0.042692
17	C	0.003358

18	C	0.022209
19	C	0.009382
20	C	-0.040674
21	H	0.041973
22	C	0.022463
23	C	-0.039723
24	H	0.040504
25	C	0.011383
26	C	-0.081958
27	H	0.024949
28	H	0.035361
29	H	0.04084
30	C	-0.071919
31	H	0.040434
32	H	0.042976
33	H	0.038731
34	C	-0.085349
35	H	0.031277
36	H	0.038669
37	H	0.022109
38	C	0.034537
39	C	0.021663
40	C	-0.034199
41	H	0.043654
42	C	-0.023851
43	H	0.047834
44	C	-0.035991
45	H	0.042689
46	C	0.014944
47	C	-0.022972
48	H	0.03445
49	C	-0.008693
50	C	-0.054069
51	C	-0.040027
52	C	0.016745
53	C	-0.049701
54	C	0.01124
55	C	-0.054834
56	C	-0.073737
57	C	0.225109
58	C	0.219079
59	O	-0.112031
60	O	-0.229947
61	O	-0.111784
62	O	-0.252292
63	C	0.047706

64	C	-0.078886
65	C	0.048118
66	C	-0.079074
67	H	0.030365
68	H	0.025968
69	H	0.033126
70	H	0.034821
71	H	0.041112
72	H	0.04113
73	H	0.038555
74	H	0.044494
75	H	0.035517
76	H	0.034578
77	H	0.043517
78	H	0.036955
79	H	0.045243
80	H	0.036067
81	H	0.034772
82	H	0.043188
83	H	0.035722
84	C	-0.049271
85	C	0.010629
86	C	0.218704
87	C	0.214516
88	C	-0.028022
89	C	-0.00362
90	C	-0.0934
91	O	-0.246154
92	O	-0.118294
93	O	-0.250136
94	O	-0.117328
95	C	0.041355
96	C	-0.080265
97	C	0.050076
98	C	-0.078579
99	H	0.036637
100	H	0.041778
101	H	0.046187
102	H	0.069144
103	H	0.024206
104	H	0.035765
105	H	0.041332
106	H	0.045416
107	H	0.035196
108	H	0.045062
109	H	0.035631

110	H	0.038383
111	H	0.025023
112	H	0.033666
113	H	0.042355
114	H	0.035635
115	C	-0.080481
116	H	0.037667
117	H	0.031645
118	H	0.032008
119	C	-0.079975
120	H	0.032047
121	H	0.028827
122	C	-0.081437
123	H	0.042764
124	H	0.037267
125	H	0.017211
126	O	-0.248372
127	H	0.042578
128	C	-0.083339
129	H	0.020791
130	H	0.036861
131	H	0.035393
132	C	-0.077212
133	H	0.040316
134	H	0.034902
135	H	0.029647
136	C	0.061597
137	C	-0.006338
138	C	-0.011328
139	C	-0.051473
140	C	-0.053532
141	C	0.000921
142	H	0.034377
143	H	0.033405
144	C	-0.075697
145	H	0.034675
146	H	0.036843
147	H	0.035003
148	C	-0.082417
149	H	0.032742
150	H	0.032666
151	H	0.012455
152	C	-0.094982
153	H	0.014154
154	H	0.021131
155	H	0.027283

	Tot	0.999996

Mo5 4r-2α

1	Mo	0.662414
2	N	-0.195915
3	C	-0.015853
4	N	0.022454
5	C	0.00892
6	H	0.075187
7	N	0.021809
8	C	0.007088
9	H	0.07422
10	C	0.01607
11	C	0.011163
12	C	-0.034754
13	H	0.040459
14	C	0.026871
15	C	-0.043247
16	H	0.041106
17	C	0.00189
18	C	0.018389
19	C	0.008332
20	C	-0.042856
21	H	0.042846
22	C	0.017967
23	C	-0.047304
24	H	0.03729
25	C	0.003414
26	C	-0.084875
27	H	0.018771
28	H	0.033758
29	H	0.041338
30	C	-0.073506
31	H	0.036325
32	H	0.041994
33	H	0.039062
34	C	-0.086017
35	H	0.034016
36	H	0.03819
37	H	0.019715
38	C	0.031508
39	C	0.04299
40	C	-0.029711
41	H	0.055945
42	C	-0.009954
43	H	0.058575

44	C	-0.032508
45	H	0.055484
46	C	0.037695
47	C	-0.032996
48	H	0.024064
49	C	-0.01676
50	C	-0.0512
51	C	-0.041578
52	C	0.01853
53	C	-0.049521
54	C	0.010269
55	C	-0.055419
56	C	-0.079182
57	C	0.219533
58	C	0.219424
59	O	-0.101351
60	O	-0.252584
61	O	-0.120404
62	O	-0.253293
63	C	0.046479
64	C	-0.079258
65	C	0.048213
66	C	-0.079724
67	H	0.032674
68	H	0.023493
69	H	0.034183
70	H	0.036584
71	H	0.034815
72	H	0.043369
73	H	0.040099
74	H	0.04416
75	H	0.034953
76	H	0.033824
77	H	0.043432
78	H	0.034577
79	H	0.04195
80	H	0.036076
81	H	0.035582
82	H	0.041584
83	H	0.029096
84	C	-0.049619
85	C	0.010092
86	C	0.217048
87	C	0.213122
88	C	-0.026078
89	C	0.002116

90	C	-0.104008
91	O	-0.255546
92	O	-0.116921
93	O	-0.246204
94	O	-0.118674
95	C	0.04051
96	C	-0.078199
97	C	0.050165
98	C	-0.078724
99	H	0.037827
100	H	0.044928
101	H	0.049233
102	H	0.063733
103	H	0.020762
104	H	0.039332
105	H	0.042056
106	H	0.045966
107	H	0.034574
108	H	0.045868
109	H	0.036119
110	H	0.03813
111	H	0.024582
112	H	0.036849
113	H	0.043116
114	H	0.037319
115	C	-0.075756
116	H	0.041704
117	H	0.038519
118	H	0.036036
119	C	-0.08574
120	H	0.023999
121	H	0.031852
122	C	-0.089342
123	H	0.040265
124	H	0.033561
125	H	0.006669
126	O	-0.261022
127	Cl	0.030219
128	Cl	0.031442
129	H	0.039373
130	C	0.050036
131	C	0.073115
132	C	0.066415
133	C	0.077556
134	C	0.078882
135	C	0.076267

136	F	-0.061538
137	F	-0.073427
138	F	-0.082752
139	F	-0.084496
140	F	-0.082019
	Tot	0.999739

Mo19 4r-2α

1	Mo	0.618959
2	N	-0.153941
3	C	0.00286
4	C	-0.046076
5	H	0.033928
6	N	0.030498
7	C	0.078978
8	C	0.033752
9	C	0.012576
10	C	-0.029752
11	H	0.044541
12	C	-0.017299
13	C	-0.030972
14	H	0.043772
15	C	0.011966
16	C	-0.020333
17	H	0.003814
18	C	-0.010181
19	H	0.015931
20	C	0.040341
21	C	0.027935
22	C	-0.028716
23	H	0.046781
24	C	-0.01369
25	H	0.051121
26	C	-0.031287
27	H	0.044893
28	C	0.02074
29	C	-0.023256
30	H	0.029079
31	C	-0.012892
32	C	-0.051788
33	C	-0.052367
34	C	0.033483
35	C	-0.048212
36	C	0.008972
37	C	-0.051185
38	C	-0.071757

39	C	0.21632
40	C	0.219223
41	O	-0.107785
42	O	-0.264718
43	O	-0.120973
44	O	-0.253913
45	C	0.047621
46	C	-0.079012
47	C	0.048266
48	C	-0.079253
49	H	0.036231
50	H	0.030173
51	H	0.035279
52	H	0.040405
53	H	0.04523
54	H	0.04405
55	H	0.040533
56	H	0.043133
57	H	0.036018
58	H	0.03398
59	H	0.043608
60	H	0.034877
61	H	0.043071
62	H	0.036267
63	H	0.036031
64	H	0.041309
65	H	0.036549
66	C	-0.050129
67	C	0.010368
68	C	0.216174
69	C	0.217044
70	C	-0.027751
71	C	-0.001845
72	C	-0.099831
73	O	-0.237533
74	O	-0.108927
75	O	-0.248333
76	O	-0.118224
77	C	0.044545
78	C	-0.078229
79	C	0.050215
80	C	-0.078218
81	H	0.043893
82	H	0.044166
83	H	0.048933
84	H	0.074271

85	H	0.024214
86	H	0.04027
87	H	0.042017
88	H	0.046418
89	H	0.035107
90	H	0.046003
91	H	0.03615
92	H	0.03945
93	H	0.030051
94	H	0.036644
95	H	0.043924
96	H	0.037666
97	C	-0.010552
98	H	0.012146
99	C	-0.009826
100	H	0.016671
101	C	-0.076838
102	H	0.034404
103	H	0.025365
104	H	0.031932
105	C	-0.079448
106	H	0.030415
107	H	0.024929
108	H	0.035483
109	C	-0.082027
110	H	0.02999
111	H	0.019298
112	H	0.030996
113	C	-0.08248
114	H	0.029129
115	H	0.028399
116	H	0.020002
117	C	0.016748
118	H	0.045224
119	C	-0.081994
120	H	0.045461
121	H	0.036061
122	H	0.032679
123	C	-0.083968
124	H	0.041002
125	H	0.025028
126	H	0.035073
127	C	-0.089458
128	H	0.015565
129	H	0.025764
130	H	0.037479

131	C	-0.074648
132	H	0.029324
133	H	0.027796
134	H	0.045817
135	C	-0.076466
136	H	0.026269
137	H	0.035884
138	H	0.030004
139	C	-0.083899
140	H	0.029522
141	H	0.015343
142	H	0.026491
143	C	-0.076988
144	H	0.034456
145	H	0.030077
146	H	0.033074
147	C	-0.084956
148	H	0.029561
149	H	0.012529
150	H	0.031599
151	Br	-0.203316
152	H	0.051452
	Tot	0.999814

Mo16 4r-2α

1	Mo	0.659051
2	N	-0.168177
3	C	0.011474
4	C	-0.045132
5	H	0.035121
6	N	0.036826
7	C	0.079452
8	C	0.033015
9	C	0.011889
10	C	-0.030363
11	H	0.045072
12	C	-0.017448
13	C	-0.029237
14	H	0.045278
15	C	0.013023
16	C	-0.017735
17	H	0.007048
18	C	-0.009764
19	H	0.01619
20	C	0.039023
21	C	0.029055

22	C	-0.026932
23	H	0.046073
24	C	-0.014496
25	H	0.050299
26	C	-0.031928
27	H	0.043674
28	C	0.017972
29	C	-0.031808
30	H	0.024191
31	C	-0.014868
32	C	-0.051154
33	C	-0.046021
34	C	0.01788
35	C	-0.046778
36	C	0.008428
37	C	-0.050475
38	C	-0.078398
39	C	0.224238
40	C	0.211946
41	O	-0.116079
42	O	-0.249315
43	O	-0.113816
44	O	-0.27277
45	C	0.047326
46	C	-0.081867
47	C	0.048251
48	C	-0.079536
49	H	0.033985
50	H	0.029576
51	H	0.03575
52	H	0.038027
53	H	0.03659
54	H	0.042932
55	H	0.039086
56	H	0.043955
57	H	0.035138
58	H	0.033525
59	H	0.042703
60	H	0.028768
61	H	0.045895
62	H	0.035898
63	H	0.031202
64	H	0.042131
65	H	0.031921
66	C	-0.048604
67	C	0.010257

68	C	0.215216
69	C	0.217914
70	C	-0.026881
71	C	-0.00036
72	C	-0.099064
73	O	-0.23711
74	O	-0.109782
75	O	-0.249417
76	O	-0.119629
77	C	0.044504
78	C	-0.079183
79	C	0.049902
80	C	-0.078808
81	H	0.041956
82	H	0.043146
83	H	0.047993
84	H	0.070548
85	H	0.025618
86	H	0.038939
87	H	0.041784
88	H	0.045572
89	H	0.034747
90	H	0.045395
91	H	0.035688
92	H	0.042631
93	H	0.026846
94	H	0.035445
95	H	0.043571
96	H	0.036444
97	O	-0.257596
98	C	-0.010704
99	H	0.009673
100	C	-0.011626
101	H	0.016725
102	C	0.063989
103	C	0.081541
104	C	0.073729
105	C	0.081315
106	C	0.078299
107	C	0.077077
108	C	-0.076741
109	H	0.033081
110	H	0.025476
111	H	0.032236
112	C	-0.080407
113	H	0.029716

114	H	0.023721
115	H	0.034251
116	C	-0.080913
117	H	0.029494
118	H	0.024881
119	H	0.031028
120	C	-0.083469
121	H	0.02893
122	H	0.027909
123	H	0.01776
124	C	0.018143
125	H	0.04588
126	C	-0.080022
127	H	0.046483
128	H	0.036177
129	H	0.034273
130	C	-0.084353
131	H	0.043728
132	H	0.026979
133	H	0.034183
134	C	-0.084898
135	H	0.021234
136	H	0.026184
137	H	0.038128
138	C	-0.075875
139	H	0.030377
140	H	0.028045
141	H	0.043943
142	C	-0.078172
143	H	0.028674
144	H	0.029591
145	H	0.030631
146	C	-0.083117
147	H	0.022936
148	H	0.016263
149	H	0.029515
150	C	-0.077728
151	H	0.034673
152	H	0.029512
153	H	0.032953
154	C	-0.085407
155	H	0.029786
156	H	0.013094
157	H	0.031331
158	F	-0.077221
159	F	-0.081022

160	F	-0.083909
161	F	-0.077115
162	F	-0.055422
163	H	0.052038
	Tot	0.999919

Mo17 4r-2α

1	Mo	0.661301
2	N	-0.167321
3	C	0.011848
4	C	-0.04491
5	H	0.035335
6	N	0.037046
7	C	0.080122
8	C	0.03369
9	C	0.012212
10	C	-0.029464
11	H	0.045159
12	C	-0.018766
13	C	-0.029682
14	H	0.044908
15	C	0.013175
16	C	-0.015471
17	H	0.010423
18	C	-0.0102
19	H	0.016777
20	C	0.044653
21	C	-0.027114
22	C	-0.026714
23	H	0.050507
24	C	-0.015542
25	H	0.051177
26	C	-0.025689
27	H	0.045246
28	C	0.03001
29	C	-0.031548
30	H	0.024418
31	C	-0.014071
32	C	-0.051354
33	C	-0.042503
34	C	0.017444
35	C	-0.045965
36	C	0.008654
37	C	-0.050744
38	C	-0.078004
39	C	0.224469

40	C	0.211885
41	O	-0.116126
42	O	-0.24892
43	O	-0.114527
44	O	-0.271763
45	C	0.047583
46	C	-0.081677
47	C	0.048106
48	C	-0.079597
49	H	0.034907
50	H	0.029656
51	H	0.035967
52	H	0.037894
53	H	0.03712
54	H	0.042011
55	H	0.038991
56	H	0.043755
57	H	0.035255
58	H	0.033331
59	H	0.042487
60	H	0.030254
61	H	0.045708
62	H	0.035852
63	H	0.031636
64	H	0.042112
65	H	0.032521
66	C	-0.054141
67	C	0.01034
68	C	0.216434
69	C	0.215155
70	C	-0.025546
71	C	0.002657
72	C	-0.100521
73	O	-0.239281
74	O	-0.117366
75	O	-0.249794
76	O	-0.118053
77	C	0.044515
78	C	-0.079111
79	C	0.050141
80	C	-0.079003
81	H	0.043456
82	H	0.04372
83	H	0.047918
84	H	0.07228
85	H	0.018129

86	H	0.039066
87	H	0.041551
88	H	0.046683
89	H	0.035089
90	H	0.045361
91	H	0.034972
92	H	0.040403
93	H	0.029858
94	H	0.035813
95	H	0.043208
96	H	0.037176
97	O	-0.258747
98	C	0.029074
99	C	0.062133
100	C	0.080344
101	C	0.075234
102	C	0.082395
103	C	0.079047
104	C	0.078037
105	C	-0.084554
106	H	0.029871
107	H	0.015393
108	H	0.031434
109	C	-0.076959
110	H	0.028805
111	H	0.03439
112	H	0.03166
113	C	0.019152
114	H	0.045739
115	C	-0.079899
116	H	0.046456
117	H	0.036648
118	H	0.034354
119	C	-0.084692
120	H	0.043721
121	H	0.025402
122	H	0.034253
123	C	-0.082981
124	H	0.022911
125	H	0.027768
126	H	0.039157
127	C	-0.075049
128	H	0.031252
129	H	0.028848
130	H	0.043915
131	C	-0.076875

132	H	0.032474
133	H	0.031537
134	H	0.027956
135	C	-0.083891
136	H	0.022768
137	H	0.01596
138	H	0.029647
139	C	-0.077336
140	H	0.034785
141	H	0.029863
142	H	0.033007
143	C	-0.08578
144	H	0.029015
145	H	0.012201
146	H	0.031655
147	F	-0.077805
148	F	-0.08194
149	F	-0.083237
150	F	-0.078231
151	F	-0.060295
152	C	-0.092835
153	H	0.029124
154	H	0.018379
155	H	0.003297
156	H	0.041573
157	H	0.051496
	Tot	1.000065

Mo21 4r-2α

1	Mo	0.662799
2	N	-0.147568
3	C	0.011616
4	C	-0.044815
5	H	0.033926
6	N	0.039445
7	C	0.081092
8	C	0.033348
9	C	0.013653
10	C	-0.027662
11	H	0.047005
12	C	-0.017463
13	C	-0.031402
14	H	0.044598
15	C	0.011334
16	C	-0.015182
17	H	0.009678

18	C	-0.011848
19	H	0.010222
20	C	0.04139
21	C	0.029765
22	C	-0.026044
23	H	0.048373
24	C	-0.009758
25	H	0.052679
26	C	-0.029132
27	H	0.046182
28	C	0.022686
29	C	-0.020084
30	H	0.029922
31	C	-0.015739
32	C	-0.048286
33	C	-0.052249
34	C	0.036169
35	C	-0.046131
36	C	0.008978
37	C	-0.051349
38	C	-0.076847
39	C	0.213888
40	C	0.22062
41	O	-0.108488
42	O	-0.263286
43	O	-0.117747
44	O	-0.258152
45	C	0.046003
46	C	-0.079828
47	C	0.048803
48	C	-0.078993
49	H	0.036414
50	H	0.027603
51	H	0.035798
52	H	0.04266
53	H	0.040755
54	H	0.044817
55	H	0.04073
56	H	0.043808
57	H	0.036298
58	H	0.034361
59	H	0.043756
60	H	0.032409
61	H	0.042592
62	H	0.036064
63	H	0.035232

64	H	0.040831
65	H	0.034735
66	C	-0.049219
67	C	0.010405
68	C	0.217388
69	C	0.216404
70	C	-0.022717
71	C	0.010363
72	C	-0.095257
73	O	-0.234262
74	O	-0.110906
75	O	-0.247876
76	O	-0.116669
77	C	0.044324
78	C	-0.078643
79	C	0.050887
80	C	-0.077664
81	H	0.042563
82	H	0.048955
83	H	0.052264
84	H	0.080432
85	H	0.02417
86	H	0.041396
87	H	0.042319
88	H	0.047195
89	H	0.036192
90	H	0.046715
91	H	0.036269
92	H	0.038834
93	H	0.029553
94	H	0.036679
95	H	0.044052
96	H	0.037829
97	O	-0.299598
98	C	-0.012279
99	H	0.009628
100	C	-0.009646
101	H	0.016201
102	C	-0.076213
103	H	0.035924
104	H	0.025124
105	H	0.032859
106	C	-0.078014
107	H	0.031739
108	H	0.027355
109	H	0.036152

110	C	-0.082385
111	H	0.032282
112	H	0.019169
113	H	0.030549
114	C	-0.082541
115	H	0.031254
116	H	0.028725
117	H	0.018014
118	C	0.018503
119	H	0.046573
120	C	-0.083066
121	H	0.045948
122	H	0.035387
123	H	0.032081
124	C	-0.082385
125	H	0.043062
126	H	0.024239
127	H	0.036565
128	C	-0.09252
129	H	0.012851
130	H	0.019154
131	H	0.036473
132	C	-0.073391
133	H	0.030318
134	H	0.028548
135	H	0.047527
136	C	-0.077458
137	H	0.038487
138	H	0.029905
139	H	0.02513
140	C	-0.083574
141	H	0.031088
142	H	0.013716
143	H	0.02649
144	C	-0.078634
145	H	0.03512
146	H	0.023301
147	H	0.035039
148	C	-0.084402
149	H	0.031297
150	H	0.018534
151	H	0.026726
152	S	0.510929
153	O	-0.307416
154	O	-0.292521
155	C	0.285567

156	F	-0.07258
157	F	-0.081104
158	F	-0.082544
159	H	0.051633
	Tot	0.999852

Mo6 4r-2α

1	Mo	0.660144
2	N	-0.154228
3	C	0.018905
4	N	-0.014705
5	C	0.019984
6	H	0.045202
7	N	-0.012424
8	C	0.020232
9	H	0.048065
10	C	0.014187
11	C	0.01472
12	C	-0.033121
13	H	0.041947
14	C	0.025253
15	C	-0.045591
16	H	0.039239
17	C	0.000052
18	C	0.018101
19	C	0.012951
20	C	-0.039999
21	H	0.044876
22	C	0.017794
23	C	-0.046134
24	H	0.036275
25	C	0.003383
26	C	-0.08655
27	H	0.014241
28	H	0.034317
29	H	0.041616
30	C	-0.072794
31	H	0.039585
32	H	0.043364
33	H	0.036983
34	C	-0.085457
35	H	0.033546
36	H	0.036319
37	H	0.019658
38	C	0.044808
39	C	-0.022486

40	C	0.023388
41	C	-0.027038
42	H	0.044579
43	C	0.018972
44	C	-0.035346
45	C	-0.020405
46	H	0.02684
47	C	-0.013579
48	C	-0.047943
49	C	-0.053933
50	C	0.034104
51	C	-0.050727
52	C	0.009005
53	C	-0.056307
54	C	-0.074206
55	C	0.224186
56	C	0.218079
57	O	-0.105148
58	O	-0.235984
59	O	-0.115413
60	O	-0.254876
61	C	0.049375
62	C	-0.079545
63	C	0.048287
64	C	-0.079115
65	H	0.032306
66	H	0.023052
67	H	0.035218
68	H	0.037527
69	H	0.045223
70	H	0.040709
71	H	0.039329
72	H	0.044692
73	H	0.035421
74	H	0.034691
75	H	0.043825
76	H	0.036642
77	H	0.045999
78	H	0.036241
79	H	0.034836
80	H	0.04333
81	H	0.036701
82	C	-0.045948
83	C	0.010557
84	C	0.216943
85	C	0.213046

86	C	-0.024919
87	C	0.008596
88	C	-0.097271
89	O	-0.250206
90	O	-0.119348
91	O	-0.248014
92	O	-0.117638
93	C	0.042641
94	C	-0.079281
95	C	0.050333
96	C	-0.078339
97	H	0.034748
98	H	0.046677
99	H	0.050443
100	H	0.081479
101	H	0.029497
102	H	0.039818
103	H	0.041734
104	H	0.04589
105	H	0.035576
106	H	0.045861
107	H	0.036098
108	H	0.040556
109	H	0.026398
110	H	0.035509
111	H	0.043046
112	H	0.036874
113	C	-0.074224
114	H	0.043612
115	H	0.036316
116	H	0.039755
117	C	-0.088968
118	H	0.014258
119	H	0.035292
120	C	-0.083651
121	H	0.041427
122	H	0.031457
123	H	0.01837
124	S	0.502232
125	O	-0.303533
126	O	-0.30931
127	C	0.286595
128	F	-0.085742
129	F	-0.085862
130	F	-0.082919
131	O	-0.313638

132	H	0.033333
133	H	0.050274
134	H	0.048081
135	H	0.046253
136	H	0.041529
137	C	-0.071591
138	H	0.037167
139	H	0.045995
140	H	0.040005
141	C	-0.071906
142	H	0.040729
143	H	0.038508
144	H	0.043745
	Tot	1.000195

Mo8 4r-2α

1	Mo	0.647681
2	N	-0.156899
3	C	-0.025604
4	N	0.016555
5	C	0.003994
6	H	0.071491
7	N	0.024511
8	C	0.006098
9	H	0.07038
10	C	0.046982
11	C	-0.049309
12	C	-0.043236
13	H	0.033895
14	C	-0.04082
15	C	-0.039309
16	H	0.035058
17	C	-0.047477
18	C	0.043826
19	C	-0.050786
20	C	-0.037684
21	H	0.029585
22	C	-0.042604
23	C	-0.041426
24	H	0.023535
25	C	-0.05238
26	C	0.039392
27	C	0.024885
28	C	-0.02744
29	H	0.047306
30	C	-0.011867

31	H	0.053963
32	C	-0.026585
33	H	0.0522
34	C	-0.028434
35	C	-0.015653
36	H	0.031108
37	C	-0.009052
38	C	-0.049435
39	C	-0.050852
40	C	0.037222
41	C	-0.048158
42	C	0.009567
43	C	-0.052911
44	C	-0.073017
45	C	0.222924
46	C	0.215208
47	O	-0.109624
48	O	-0.234964
49	O	-0.117388
50	O	-0.260257
51	C	0.048219
52	C	-0.07926
53	C	0.048156
54	C	-0.079325
55	H	0.035072
56	H	0.029686
57	H	0.037373
58	H	0.038071
59	H	0.046279
60	H	0.040976
61	H	0.039273
62	H	0.044439
63	H	0.03503
64	H	0.033724
65	H	0.043849
66	H	0.034972
67	H	0.045771
68	H	0.036427
69	H	0.034539
70	H	0.043041
71	H	0.036884
72	C	-0.04514
73	C	0.010153
74	C	0.210731
75	C	0.220708
76	C	-0.028669

77	C	0.019209
78	C	-0.104293
79	O	-0.244061
80	O	-0.113588
81	O	-0.249163
82	O	-0.116489
83	C	0.051054
84	C	-0.077552
85	C	0.050007
86	C	-0.08172
87	H	0.042981
88	H	0.046741
89	H	0.048395
90	H	0.079062
91	H	0.033903
92	H	0.045713
93	H	0.04191
94	H	0.046907
95	H	0.032248
96	H	0.043794
97	H	0.027747
98	H	0.047833
99	H	0.040097
100	H	0.036722
101	H	0.045925
102	H	0.037045
103	S	0.497332
104	O	-0.311178
105	O	-0.296025
106	C	0.28643
107	F	-0.085039
108	F	-0.085748
109	F	-0.093595
110	O	-0.317522
111	C	0.027088
112	C	-0.08294
113	H	0.013215
114	H	0.034214
115	H	0.028244
116	C	-0.076426
117	H	0.036906
118	H	0.032785
119	H	0.031231
120	C	-0.087015
121	H	0.034334
122	H	0.008564

123	H	0.029191
124	H	0.036073
125	H	0.031211
126	H	0.025764
127	H	0.028923
128	H	0.025701
129	H	0.024688
130	H	0.038424
131	H	0.022481
132	H	0.027896
133	H	0.020146
134	H	0.030244
135	H	0.028278
136	H	0.02489
137	H	0.037782
138	H	0.034321
139	H	0.026449
140	H	0.034387
141	H	0.016871
142	H	0.025909
	Tot	1.000085

Mo9 4r-2α

1	Mo	0.65719
2	N	-0.173531
3	C	-0.016641
4	N	0.018661
5	C	-0.00086
6	H	0.06896
7	N	0.018694
8	C	-0.00082
9	H	0.068849
10	C	0.037123
11	C	-0.044563
12	C	-0.038855
13	H	0.030371
14	C	-0.040993
15	C	-0.039164
16	H	0.035978
17	C	-0.049986
18	C	0.039661
19	C	-0.049733
20	C	-0.039754
21	H	0.02699
22	C	-0.040754
23	C	-0.037295

24	H	0.027781
25	C	-0.044549
26	C	0.034696
27	C	0.018758
28	C	-0.031456
29	H	0.04523
30	C	-0.018697
31	H	0.051691
32	C	-0.031794
33	H	0.049422
34	C	-0.037112
35	C	-0.031058
36	H	0.028274
37	C	-0.016031
38	C	-0.05261
39	C	-0.044373
40	C	0.022825
41	C	-0.050194
42	C	0.008791
43	C	-0.050438
44	C	-0.078513
45	C	0.212856
46	C	0.219775
47	O	-0.118403
48	O	-0.2527
49	O	-0.117976
50	O	-0.258122
51	C	0.047123
52	C	-0.080079
53	C	0.048392
54	C	-0.078859
55	H	0.033416
56	H	0.026845
57	H	0.033475
58	H	0.038285
59	H	0.034737
60	H	0.04456
61	H	0.038813
62	H	0.043457
63	H	0.03742
64	H	0.03333
65	H	0.042883
66	H	0.036571
67	H	0.043838
68	H	0.033304
69	H	0.034556

70	H	0.041312
71	H	0.028599
72	C	-0.045566
73	C	0.009269
74	C	0.210848
75	C	0.220504
76	C	-0.029367
77	C	0.013656
78	C	-0.107602
79	O	-0.244616
80	O	-0.113547
81	O	-0.250162
82	O	-0.11629
83	C	0.050721
84	C	-0.077596
85	C	0.049609
86	C	-0.080949
87	H	0.040877
88	H	0.044818
89	H	0.047417
90	H	0.073764
91	H	0.033805
92	H	0.044618
93	H	0.041053
94	H	0.045898
95	H	0.031528
96	H	0.047413
97	H	0.02732
98	H	0.047331
99	H	0.040026
100	H	0.036548
101	H	0.045624
102	H	0.037313
103	O	-0.265935
104	C	0.027026
105	C	-0.084767
106	H	0.011571
107	H	0.033081
108	H	0.026332
109	C	-0.076939
110	H	0.035837
111	H	0.032867
112	H	0.030324
113	C	-0.087436
114	H	0.033115
115	H	0.008978

116	H	0.029397
117	H	0.031744
118	H	0.028242
119	H	0.02026
120	H	0.026425
121	H	0.034393
122	H	0.038274
123	H	0.025868
124	H	0.037289
125	H	0.02682
126	H	0.023883
127	H	0.026615
128	H	0.016603
129	H	0.030216
130	H	0.038517
131	H	0.036679
132	H	0.028951
133	H	0.038052
134	H	0.032974
135	H	0.03008
136	C	0.051282
137	C	0.070413
138	C	0.067364
139	C	0.079791
140	C	0.080862
141	C	0.076117
142	F	-0.068409
143	F	-0.0785
144	F	-0.082901
145	F	-0.084163
146	F	-0.089157
	Tot	0.99988

Mo7 4r-2α

1	Mo	0.647991
2	N	-0.178872
3	C	0.01833
4	N	-0.011562
5	C	0.015691
6	H	0.04445
7	N	-0.017042
8	C	0.014511
9	H	0.05053
10	C	0.017123
11	C	-0.001126
12	C	-0.04005

13	H	0.042396
14	C	0.027864
15	C	-0.03858
16	H	0.041857
17	C	0.000715
18	C	0.021477
19	C	0.00441
20	C	-0.040315
21	H	0.041861
22	C	0.022805
23	C	-0.039696
24	H	0.040353
25	C	0.008547
26	C	-0.083625
27	H	0.015878
28	H	0.028486
29	H	0.043746
30	C	-0.071775
31	H	0.039531
32	H	0.044432
33	H	0.038366
34	C	-0.082583
35	H	0.035355
36	H	0.038563
37	H	0.022972
38	C	0.032581
39	C	0.024786
40	C	-0.032698
41	C	-0.020243
42	H	0.049035
43	C	-0.036867
44	C	0.016107
45	C	-0.030976
46	H	0.026517
47	C	-0.014749
48	C	-0.045502
49	C	-0.042342
50	C	0.020173
51	C	-0.050256
52	C	0.010462
53	C	-0.054603
54	C	-0.074095
55	C	0.223229
56	C	0.215396
57	O	-0.111072
58	O	-0.235646

59	O	-0.117199
60	O	-0.263021
61	C	0.047546
62	C	-0.079634
63	C	0.047246
64	C	-0.079571
65	H	0.032323
66	H	0.026194
67	H	0.033556
68	H	0.035636
69	H	0.042402
70	H	0.040615
71	H	0.038154
72	H	0.043329
73	H	0.035381
74	H	0.033769
75	H	0.042592
76	H	0.03716
77	H	0.04479
78	H	0.035937
79	H	0.034098
80	H	0.042197
81	H	0.033617
82	C	-0.052054
83	C	0.010756
84	C	0.217886
85	C	0.215375
86	C	-0.027453
87	C	0.003937
88	C	-0.106873
89	O	-0.240903
90	O	-0.115994
91	O	-0.248119
92	O	-0.117127
93	C	0.04288
94	C	-0.079688
95	C	0.050095
96	C	-0.078845
97	H	0.042981
98	H	0.043036
99	H	0.046703
100	H	0.072089
101	H	0.021104
102	H	0.037748
103	H	0.041608
104	H	0.046169

105	H	0.035113
106	H	0.045255
107	H	0.035499
108	H	0.03945
109	H	0.026795
110	H	0.035065
111	H	0.042812
112	H	0.036305
113	C	-0.078551
114	H	0.034124
115	H	0.035503
116	H	0.037015
117	C	-0.080711
118	H	0.023021
119	H	0.037191
120	C	-0.078191
121	H	0.04456
122	H	0.030703
123	H	0.018039
124	O	-0.290199
125	H	0.042471
126	H	0.051296
127	H	0.043521
128	C	-0.077468
129	H	0.041204
130	H	0.037141
131	H	0.026969
132	C	-0.088078
133	H	0.018319
134	H	0.032144
135	H	0.035253
136	C	0.025991
137	H	0.035998
138	C	0.295348
139	F	-0.102794
140	F	-0.103936
141	F	-0.071269
142	C	0.290008
143	F	-0.096431
144	F	-0.101302
145	F	-0.09695
146	H	0.043868
147	H	0.045285
	Tot	1.000063

Mo22 4r-2α

1	Mo	0.622634
2	N	-0.17192
3	C	0.002371
4	C	-0.045911
5	H	0.034046
6	N	0.031167
7	C	0.079927
8	C	0.030827
9	C	0.012003
10	C	-0.031477
11	H	0.045277
12	C	-0.026588
13	C	-0.038462
14	H	0.041526
15	C	0.005171
16	C	-0.016352
17	H	0.008533
18	C	-0.012305
19	H	0.015501
20	C	0.052734
21	C	-0.013959
22	C	-0.010963
23	H	0.055206
24	C	-0.011124
25	H	0.056649
26	C	-0.016346
27	H	0.053979
28	C	-0.015964
29	C	-0.020619
30	H	0.029424
31	C	-0.015115
32	C	-0.057966
33	C	-0.051367
34	C	0.039327
35	C	-0.047929
36	C	0.008613
37	C	-0.048376
38	C	-0.070731
39	C	0.216906
40	C	0.218797
41	O	-0.107106
42	O	-0.269825
43	O	-0.122913
44	O	-0.253914
45	C	0.046922
46	C	-0.078007

47	C	0.04887
48	C	-0.079777
49	H	0.037556
50	H	0.030991
51	H	0.036274
52	H	0.041511
53	H	0.046904
54	H	0.045316
55	H	0.041424
56	H	0.04432
57	H	0.035223
58	H	0.033288
59	H	0.04358
60	H	0.033708
61	H	0.041781
62	H	0.036985
63	H	0.038809
64	H	0.041587
65	H	0.036878
66	C	-0.045853
67	C	0.009954
68	C	0.217288
69	C	0.210871
70	C	-0.024699
71	C	-0.000579
72	C	-0.095295
73	O	-0.251421
74	O	-0.120313
75	O	-0.249877
76	O	-0.11506
77	C	0.043323
78	C	-0.078274
79	C	0.050152
80	C	-0.076833
81	H	0.045112
82	H	0.046939
83	H	0.05185
84	H	0.074265
85	H	0.028772
86	H	0.041695
87	H	0.042141
88	H	0.045337
89	H	0.036422
90	H	0.046641
91	H	0.036568
92	H	0.040097

93	H	0.028426
94	H	0.036135
95	H	0.043842
96	H	0.037506
97	C	0.018197
98	H	0.044382
99	C	-0.080263
100	H	0.045848
101	H	0.036526
102	H	0.03432
103	C	-0.083528
104	H	0.040371
105	H	0.025755
106	H	0.036451
107	C	-0.090479
108	H	0.014428
109	H	0.025297
110	H	0.035866
111	C	-0.076084
112	H	0.030688
113	H	0.025691
114	H	0.046435
115	C	-0.077064
116	H	0.024358
117	H	0.035111
118	H	0.031632
119	C	-0.086123
120	H	0.030677
121	H	0.013452
122	H	0.026487
123	C	-0.076457
124	H	0.034058
125	H	0.0313
126	H	0.032608
127	C	-0.085938
128	H	0.023639
129	H	0.013187
130	H	0.032697
131	H	0.050655
132	C	0.30155
133	F	-0.095582
134	F	-0.082541
135	F	-0.101271
136	Br	-0.197627
137	H	0.048545
	Tot	0.999915

Mo1 4r-2β

1	Mo	0.664633
2	N	-0.151424
3	C	-0.015548
4	N	0.027252
5	C	0.011891
6	H	0.075138
7	N	0.025963
8	C	0.010537
9	H	0.07445
10	C	0.018609
11	C	-0.001627
12	C	-0.046122
13	H	0.037819
14	C	0.018629
15	C	-0.036679
16	H	0.045199
17	C	0.01099
18	C	0.022452
19	C	0.006417
20	C	-0.041675
21	H	0.038994
22	C	0.022775
23	C	-0.036715
24	H	0.045569
25	C	0.014271
26	C	-0.084328
27	H	0.037094
28	H	0.036968
29	H	0.021178
30	C	-0.072132
31	H	0.039778
32	H	0.036365
33	H	0.044589
34	C	-0.093388
35	H	0.033669
36	H	0.017074
37	H	0.034149
38	C	0.037162
39	C	0.029325
40	C	-0.0331
41	H	0.047276
42	C	-0.011923
43	H	0.052357
44	C	-0.03594

45	H	0.044664
46	C	0.027294
47	C	-0.007493
48	H	0.045209
49	C	-0.019549
50	C	0.002954
51	C	-0.085169
52	C	0.016942
53	C	-0.047331
54	C	0.013812
55	C	-0.054955
56	C	-0.07467
57	C	0.226154
58	C	0.220694
59	O	-0.10399
60	O	-0.236754
61	O	-0.108502
62	O	-0.253455
63	C	0.043874
64	C	-0.08289
65	C	0.048427
66	C	-0.079137
67	H	0.028647
68	H	0.012624
69	H	0.035258
70	H	0.03252
71	H	0.039233
72	H	0.032552
73	H	0.037
74	H	0.045288
75	H	0.04343
76	H	0.034808
77	H	0.035848
78	H	0.025807
79	H	0.047253
80	H	0.033565
81	H	0.028241
82	H	0.041531
83	H	0.044439
84	C	-0.051964
85	C	0.011264
86	C	0.215636
87	C	0.218502
88	C	-0.028329
89	C	0.012842
90	C	-0.119864

91	O	-0.249747
92	O	-0.115827
93	O	-0.247844
94	O	-0.118453
95	C	0.049924
96	C	-0.078007
97	C	0.050086
98	C	-0.078885
99	H	0.029562
100	H	0.044147
101	H	0.04508
102	H	0.069163
103	H	0.024452
104	H	0.038119
105	H	0.045439
106	H	0.042617
107	H	0.033356
108	H	0.034382
109	H	0.046021
110	H	0.037865
111	H	0.047421
112	H	0.036507
113	H	0.036214
114	H	0.044689
115	C	-0.073573
116	H	0.037111
117	H	0.041637
118	H	0.037674
119	C	-0.081371
120	H	0.013288
121	H	0.036448
122	H	0.041657
123	C	-0.085591
124	H	0.02804
125	H	0.040628
126	H	0.02812
127	O	-0.292267
128	S	0.519367
129	O	-0.28462
130	O	-0.296439
131	C	0.284004
132	F	-0.083092
133	F	-0.086472
134	F	-0.087332
135	C	-0.078063
136	H	0.04223

137	H	0.029138
138	H	0.03368
139	C	-0.087974
140	H	0.036756
141	H	0.029135
142	H	0.021632
	Tot	1.000255

Mo2 4r-2β

1	Mo	0.668259
2	N	-0.151001
3	C	-0.01507
4	N	0.027126
5	C	0.012986
6	H	0.075377
7	N	0.025282
8	C	0.011528
9	H	0.075325
10	C	0.018867
11	C	-0.000237
12	C	-0.045588
13	H	0.038564
14	C	0.020454
15	C	-0.036887
16	H	0.04573
17	C	0.011528
18	C	0.020418
19	C	0.004622
20	C	-0.043122
21	H	0.040531
22	C	0.021384
23	C	-0.039776
24	H	0.043725
25	C	0.011801
26	C	-0.081792
27	H	0.031737
28	H	0.040436
29	H	0.026237
30	C	-0.07454
31	H	0.043304
32	H	0.037812
33	H	0.032812
34	C	-0.092983
35	H	0.036963
36	H	0.015982
37	H	0.033124

38	C	0.041335
39	C	0.027627
40	C	-0.02657
41	H	0.045081
42	C	-0.014135
43	H	0.051132
44	C	-0.026755
45	H	0.049382
46	C	-0.022907
47	C	-0.012213
48	H	0.041171
49	C	-0.020609
50	C	0.000262
51	C	-0.081611
52	C	0.014911
53	C	-0.047403
54	C	0.010141
55	C	-0.050236
56	C	-0.081623
57	C	0.231944
58	C	0.216486
59	O	-0.108705
60	O	-0.239946
61	O	-0.105774
62	O	-0.263251
63	C	0.045057
64	C	-0.08077
65	C	0.049345
66	C	-0.079394
67	H	0.030243
68	H	0.021865
69	H	0.03666
70	H	0.033649
71	H	0.036891
72	H	0.021705
73	H	0.039164
74	H	0.045879
75	H	0.043714
76	H	0.034029
77	H	0.034785
78	H	0.025424
79	H	0.047332
80	H	0.036175
81	H	0.0279
82	H	0.041728
83	H	0.040692

84	C	-0.053363
85	C	0.009665
86	C	0.216584
87	C	0.218218
88	C	-0.030194
89	C	0.016942
90	C	-0.122862
91	O	-0.234605
92	O	-0.116064
93	O	-0.234048
94	O	-0.113099
95	C	0.049891
96	C	-0.078021
97	C	0.05053
98	C	-0.078298
99	H	0.031506
100	H	0.042261
101	H	0.042393
102	H	0.069635
103	H	0.025438
104	H	0.039131
105	H	0.046447
106	H	0.042609
107	H	0.033638
108	H	0.034296
109	H	0.04596
110	H	0.039136
111	H	0.047053
112	H	0.036265
113	H	0.036686
114	H	0.045223
115	C	-0.072779
116	H	0.037875
117	H	0.041871
118	H	0.039647
119	C	-0.081549
120	H	0.01869
121	H	0.032777
122	H	0.043171
123	C	-0.085329
124	H	0.028372
125	H	0.041649
126	H	0.029225
127	O	-0.29109
128	S	0.519954
129	O	-0.288195

130	O	-0.295905
131	C	0.283028
132	F	-0.083508
133	F	-0.084987
134	F	-0.088251
135	C	0.028591
136	H	0.033579
137	C	-0.087796
138	H	0.016065
139	H	0.017861
140	H	0.032348
141	C	-0.079451
142	H	0.029775
143	H	0.023858
144	H	0.034088
145	C	-0.083972
146	H	0.0213
147	H	0.028448
148	H	0.030946
	Tot	0.999987

Mo3 4r-2β

1	Mo	0.665141
2	N	-0.173539
3	C	-0.016139
4	N	0.028666
5	C	0.013204
6	H	0.07577
7	N	0.026409
8	C	0.011629
9	H	0.074958
10	C	0.017027
11	C	-0.001407
12	C	-0.046829
13	H	0.037869
14	C	0.018186
15	C	-0.038232
16	H	0.043925
17	C	0.010132
18	C	0.017488
19	C	0.000255
20	C	-0.050141
21	H	0.035798
22	C	0.015762
23	C	-0.042979
24	H	0.044995

25	C	0.009883
26	C	-0.088772
27	H	0.036096
28	H	0.034176
29	H	0.015856
30	C	-0.073645
31	H	0.04188
32	H	0.03475
33	H	0.038656
34	C	-0.094114
35	H	0.033787
36	H	0.016675
37	H	0.03248
38	C	0.03301
39	C	0.047249
40	C	-0.026964
41	H	0.057814
42	C	-0.002529
43	H	0.059645
44	C	-0.030306
45	H	0.055588
46	C	0.043922
47	C	-0.007958
48	H	0.044945
49	C	-0.01869
50	C	-0.001989
51	C	-0.08429
52	C	0.017622
53	C	-0.046283
54	C	0.014457
55	C	-0.057293
56	C	-0.07383
57	C	0.22691
58	C	0.221301
59	O	-0.103705
60	O	-0.236286
61	O	-0.110097
62	O	-0.251244
63	C	0.045413
64	C	-0.081494
65	C	0.048542
66	C	-0.078418
67	H	0.024459
68	H	0.011781
69	H	0.036007
70	H	0.033386

71	H	0.039751
72	H	0.034325
73	H	0.037285
74	H	0.044871
75	H	0.043769
76	H	0.035509
77	H	0.036075
78	H	0.027678
79	H	0.046902
80	H	0.034272
81	H	0.029564
82	H	0.041975
83	H	0.04459
84	C	-0.052842
85	C	0.010305
86	C	0.218301
87	C	0.213763
88	C	-0.031585
89	C	0.011889
90	C	-0.120683
91	O	-0.246778
92	O	-0.115492
93	O	-0.248662
94	O	-0.117486
95	C	0.047849
96	C	-0.0786
97	C	0.050218
98	C	-0.078063
99	H	0.028256
100	H	0.045357
101	H	0.044928
102	H	0.068272
103	H	0.018765
104	H	0.038287
105	H	0.04579
106	H	0.042547
107	H	0.034619
108	H	0.035967
109	H	0.046371
110	H	0.03334
111	H	0.045594
112	H	0.037005
113	H	0.03582
114	H	0.044145
115	C	-0.073675
116	H	0.0378

117	H	0.040747
118	H	0.037087
119	C	-0.086014
120	H	0.008717
121	H	0.032251
122	H	0.040244
123	C	-0.087776
124	H	0.025892
125	H	0.039145
126	H	0.026701
127	O	-0.288899
128	S	0.521308
129	O	-0.281457
130	O	-0.295934
131	C	0.284197
132	F	-0.082607
133	F	-0.085775
134	F	-0.085732
135	Cl	0.017963
136	Cl	0.035707
	Tot	0.999988

Mo10 4r-2β

1	Mo	0.655076
2	N	-0.149741
3	C	-0.028699
4	N	0.071382
5	N	0.014887
6	C	0.115675
7	C	0.041177
8	C	-0.044699
9	C	-0.02525
10	H	0.047904
11	C	-0.01723
12	C	-0.011078
13	H	0.054965
14	C	-0.019319
15	C	0.039014
16	C	-0.035627
17	C	-0.023614
18	H	0.047796
19	C	-0.01816
20	C	-0.016518
21	H	0.051291
22	C	-0.028176
23	C	0.036646

24	C	0.025963
25	C	-0.0329
26	H	0.046778
27	C	-0.012264
28	H	0.052003
29	C	-0.033694
30	H	0.046343
31	C	0.025177
32	C	-0.015774
33	H	0.042537
34	C	-0.019142
35	C	0.003102
36	C	-0.086914
37	C	0.018897
38	C	-0.048352
39	C	0.012804
40	C	-0.05264
41	C	-0.073294
42	C	0.224706
43	C	0.221723
44	O	-0.106142
45	O	-0.251449
46	O	-0.105918
47	O	-0.242352
48	C	0.04556
49	C	-0.081974
50	C	0.04881
51	C	-0.07843
52	H	0.027517
53	H	0.009376
54	H	0.036847
55	H	0.033524
56	H	0.040129
57	H	0.034537
58	H	0.034662
59	H	0.047001
60	H	0.044644
61	H	0.03396
62	H	0.03701
63	H	0.026601
64	H	0.046363
65	H	0.032
66	H	0.023714
67	H	0.045333
68	H	0.046413
69	C	-0.051644

70	C	0.010712
71	C	0.214602
72	C	0.219616
73	C	-0.027827
74	C	0.009668
75	C	-0.119688
76	O	-0.243642
77	O	-0.113224
78	O	-0.248665
79	O	-0.120472
80	C	0.050125
81	C	-0.078454
82	C	0.049737
83	C	-0.079052
84	H	0.030991
85	H	0.046963
86	H	0.047451
87	H	0.069426
88	H	0.026309
89	H	0.036149
90	H	0.044992
91	H	0.042616
92	H	0.032689
93	H	0.036047
94	H	0.045827
95	H	0.036629
96	H	0.048391
97	H	0.037716
98	H	0.035333
99	H	0.044837
100	O	-0.294523
101	S	0.515422
102	O	-0.294006
103	O	-0.308899
104	C	0.282677
105	F	-0.083525
106	F	-0.087074
107	F	-0.09451
108	C	-0.076907
109	H	0.042345
110	H	0.029474
111	H	0.035739
112	C	-0.08489
113	H	0.03351
114	H	0.037427
115	H	0.020234

116	N	-0.098658
117	H	0.031947
118	H	0.051778
119	H	0.051232
120	H	0.042399
121	H	0.049966
122	H	0.03445
123	C	-0.007095
124	C	-0.028016
125	C	-0.021739
126	C	-0.024579
127	C	-0.02421
128	C	-0.01643
129	H	0.037269
130	H	0.039211
131	H	0.04894
132	H	0.046431
133	H	0.049935
	Tot	0.999972

Mo13 4r-2β

1	Mo	0.656465
2	N	-0.190986
3	C	0.024623
4	N	-0.011749
5	C	0.014863
6	N	-0.010547
7	C	0.014619
8	C	0.017186
9	C	0.005819
10	C	-0.044027
11	H	0.040239
12	C	0.021529
13	C	-0.045404
14	H	0.037693
15	C	0.005889
16	C	0.019313
17	C	0.004998
18	C	-0.043469
19	H	0.037779
20	C	0.01563
21	C	-0.040453
22	H	0.041255
23	C	0.006841
24	C	-0.085103
25	H	0.037746

26	H	0.034079
27	H	0.020648
28	C	-0.073249
29	H	0.040145
30	H	0.039968
31	H	0.03604
32	C	-0.095352
33	H	0.028578
34	H	0.011898
35	H	0.032244
36	C	0.050038
37	C	-0.019708
38	C	-0.020298
39	H	0.051768
40	C	-0.016772
41	H	0.053557
42	C	-0.017622
43	H	0.050167
44	C	-0.017872
45	C	-0.02326
46	H	0.036546
47	C	-0.010938
48	C	0.00064
49	C	-0.082545
50	C	0.016685
51	C	-0.04331
52	C	0.008683
53	C	-0.05757
54	C	-0.073369
55	C	0.220542
56	C	0.220736
57	O	-0.107445
58	O	-0.245316
59	O	-0.113224
60	O	-0.243957
61	C	0.044466
62	C	-0.074671
63	C	0.049252
64	C	-0.07928
65	H	0.025664
66	H	0.022913
67	H	0.037254
68	H	0.037595
69	H	0.034818
70	H	0.044165
71	H	0.039339

72	H	0.046053
73	H	0.044087
74	H	0.033412
75	H	0.035195
76	H	0.0273
77	H	0.036818
78	H	0.036317
79	H	0.038988
80	H	0.044685
81	H	0.041412
82	C	-0.056993
83	C	0.010121
84	C	0.215444
85	C	0.2157
86	C	-0.027425
87	C	0.016286
88	C	-0.118982
89	O	-0.250898
90	O	-0.119285
91	O	-0.248992
92	O	-0.117313
93	C	0.048896
94	C	-0.078756
95	C	0.050151
96	C	-0.080683
97	H	0.033407
98	H	0.046146
99	H	0.04477
100	H	0.065754
101	H	0.019923
102	H	0.033874
103	H	0.045818
104	H	0.042476
105	H	0.031789
106	H	0.032576
107	H	0.044952
108	H	0.039298
109	H	0.045678
110	H	0.03466
111	H	0.035925
112	H	0.043819
113	C	-0.074496
114	H	0.038591
115	H	0.04075
116	H	0.035572
117	C	-0.086163

118	H	0.016537
119	H	0.041889
120	H	0.034918
121	C	-0.083925
122	H	0.026623
123	H	0.03691
124	H	0.034582
125	O	-0.255188
126	C	0.301832
127	C	-0.04118
128	H	0.049519
129	H	0.043718
130	H	0.048784
131	H	0.041061
132	H	0.038123
133	H	0.053096
134	C	0.060054
135	C	0.073932
136	C	0.073649
137	C	0.07331
138	C	0.077877
139	C	0.075375
140	F	-0.072391
141	F	-0.084358
142	F	-0.085311
143	F	-0.084418
144	F	-0.077734
145	H	0.038757
146	F	-0.10283
147	F	-0.093788
148	F	-0.099503
	Tot	0.99999

Mo14 4r-2β

1	Mo	0.660305
2	N	-0.166758
3	C	0.025154
4	N	-0.008909
5	C	0.012052
6	N	-0.015152
7	C	0.010876
8	C	0.021969
9	C	0.001421
10	C	-0.044101
11	H	0.038783
12	C	0.018368

13	C	-0.046388
14	H	0.038078
15	C	0.004731
16	C	0.025491
17	C	0.002427
18	C	-0.043262
19	H	0.037866
20	C	0.018108
21	C	-0.039164
22	H	0.042083
23	C	0.010701
24	C	-0.084833
25	H	0.027385
26	H	0.040274
27	H	0.019917
28	C	-0.073754
29	H	0.038691
30	H	0.033706
31	H	0.040501
32	C	-0.08828
33	H	0.037367
34	H	0.014728
35	H	0.034046
36	C	0.035319
37	C	0.022746
38	C	-0.034409
39	H	0.045818
40	C	-0.015381
41	H	0.050778
42	C	-0.035133
43	H	0.043424
44	C	0.025501
45	C	-0.016456
46	H	0.040905
47	C	-0.011647
48	C	0.005901
49	C	-0.079592
50	C	0.007393
51	C	-0.05031
52	C	0.008583
53	C	-0.047057
54	C	-0.081399
55	C	0.235566
56	C	0.21494
57	O	-0.102744
58	O	-0.22858

59	O	-0.108163
60	O	-0.266467
61	C	0.042034
62	C	-0.076479
63	C	0.049095
64	C	-0.078744
65	H	0.035402
66	H	0.030733
67	H	0.029043
68	H	0.03538
69	H	0.042271
70	H	0.029612
71	H	0.039129
72	H	0.04562
73	H	0.044438
74	H	0.035193
75	H	0.035182
76	H	0.023263
77	H	0.036527
78	H	0.043698
79	H	0.036411
80	H	0.038468
81	H	0.023763
82	C	-0.053136
83	C	0.008583
84	C	0.215484
85	C	0.216465
86	C	-0.029628
87	C	0.005939
88	C	-0.124859
89	O	-0.255378
90	O	-0.119196
91	O	-0.245737
92	O	-0.11429
93	C	0.049122
94	C	-0.077987
95	C	0.049997
96	C	-0.079309
97	H	0.031
98	H	0.042425
99	H	0.041799
100	H	0.061193
101	H	0.025758
102	H	0.036937
103	H	0.046165
104	H	0.042126

105	H	0.032636
106	H	0.033199
107	H	0.045101
108	H	0.038516
109	H	0.046034
110	H	0.035636
111	H	0.03669
112	H	0.044154
113	C	-0.074224
114	H	0.038339
115	H	0.040174
116	H	0.036537
117	C	-0.079954
118	H	0.020616
119	H	0.032689
120	H	0.042058
121	C	-0.086367
122	H	0.022746
123	H	0.036754
124	H	0.032852
125	O	-0.262218
126	C	-0.077132
127	H	0.041384
128	H	0.026966
129	H	0.036676
130	C	-0.087408
131	H	0.033346
132	H	0.030562
133	H	0.021902
134	C	-0.043526
135	H	0.047421
136	H	0.040583
137	H	0.045071
138	H	0.039246
139	H	0.034434
140	H	0.053233
141	C	0.054888
142	C	0.069061
143	C	0.069814
144	C	0.076933
145	C	0.077092
146	C	0.075939
147	F	-0.067809
148	F	-0.086398
149	F	-0.083471
150	F	-0.083348

151	F	-0.074802
	Tot	1.000099

Mo15 4r-2β

1	Mo	0.674394
2	N	-0.193846
3	C	0.023355
4	N	-0.014205
5	C	0.013705
6	N	-0.011813
7	C	0.013242
8	C	0.019168
9	C	0.007871
10	C	-0.041418
11	H	0.041311
12	C	0.022485
13	C	-0.042059
14	H	0.041512
15	C	0.006987
16	C	0.019728
17	C	-0.003601
18	C	-0.052214
19	H	0.034651
20	C	0.017892
21	C	-0.038109
22	H	0.039002
23	C	0.011716
24	C	-0.087127
25	H	0.036089
26	H	0.030624
27	H	0.020142
28	C	-0.074144
29	H	0.038974
30	H	0.035987
31	H	0.040084
32	C	-0.091673
33	H	0.033402
34	H	0.016367
35	H	0.026837
36	C	0.022825
37	C	0.101088
38	C	0.089405
39	C	0.098305
40	C	0.083618
41	C	0.098224
42	C	-0.013366

43	H	0.041492
44	C	-0.012917
45	C	-0.000562
46	C	-0.07999
47	C	0.006366
48	C	-0.049746
49	C	0.008647
50	C	-0.050532
51	C	-0.083799
52	C	0.230322
53	C	0.219453
54	O	-0.107261
55	O	-0.231769
56	O	-0.104844
57	O	-0.253708
58	C	0.043892
59	C	-0.076486
60	C	0.049245
61	C	-0.078964
62	H	0.032553
63	H	0.020747
64	H	0.030348
65	H	0.034207
66	H	0.038243
67	H	0.026804
68	H	0.039186
69	H	0.046311
70	H	0.044676
71	H	0.034097
72	H	0.035577
73	H	0.028673
74	H	0.037861
75	H	0.041362
76	H	0.036687
77	H	0.040845
78	H	0.030091
79	C	-0.05947
80	C	0.009167
81	C	0.216971
82	C	0.216432
83	C	-0.029742
84	C	0.003606
85	C	-0.127307
86	O	-0.257986
87	O	-0.118139
88	O	-0.241768

89	O	-0.115338
90	C	0.04911
91	C	-0.078356
92	C	0.050205
93	C	-0.0788
94	H	0.021195
95	H	0.042446
96	H	0.043161
97	H	0.063569
98	H	0.014329
99	H	0.033584
100	H	0.046081
101	H	0.042396
102	H	0.033662
103	H	0.033925
104	H	0.045393
105	H	0.038908
106	H	0.046108
107	H	0.035166
108	H	0.036214
109	H	0.044208
110	C	-0.071555
111	H	0.04042
112	H	0.040896
113	H	0.04254
114	C	-0.08444
115	H	0.012662
116	H	0.039197
117	H	0.04075
118	C	-0.078067
119	H	0.03002
120	H	0.041403
121	H	0.03617
122	O	-0.29306
123	C	-0.04298
124	H	0.049082
125	H	0.042513
126	H	0.04556
127	H	0.039453
128	H	0.036625
129	H	0.052951
130	C	0.05923
131	C	-0.10051
132	H	0.036997
133	H	0.028121
134	H	0.032381

135	C	0.289174
136	F	-0.099583
137	F	-0.101387
138	F	-0.08489
139	C	0.293717
140	F	-0.083042
141	F	-0.070857
142	F	-0.09626
143	F	-0.066164
144	F	-0.07505
145	F	-0.056648
146	F	-0.078302
147	F	-0.068591
	Tot	0.99993

Mo11 4r-2β

1	Mo	0.640457
2	N	-0.184114
3	C	-0.016246
4	N	0.014761
5	C	0.002103
6	H	0.065861
7	N	0.019414
8	C	0.00572
9	H	0.067807
10	C	0.032822
11	C	0.021838
12	C	-0.035318
13	H	0.04444
14	C	-0.021687
15	H	0.049107
16	C	-0.040282
17	H	0.043542
18	C	0.013093
19	C	-0.023694
20	H	0.037699
21	C	-0.016635
22	C	0.001833
23	C	-0.081477
24	C	0.011769
25	C	-0.04886
26	C	0.012533
27	C	-0.055661
28	C	-0.076911
29	C	0.224982
30	C	0.222876

31	O	-0.10487
32	O	-0.219624
33	O	-0.11142
34	O	-0.24506
35	C	0.044996
36	C	-0.078961
37	C	0.048968
38	C	-0.078793
39	H	0.027347
40	H	0.021862
41	H	0.033716
42	H	0.033607
43	H	0.038875
44	H	0.032792
45	H	0.03853
46	H	0.045626
47	H	0.044649
48	H	0.03496
49	H	0.035779
50	H	0.027733
51	H	0.044278
52	H	0.035612
53	H	0.033662
54	H	0.043036
55	H	0.039656
56	C	-0.052513
57	C	0.009882
58	C	0.218688
59	C	0.219011
60	C	-0.037795
61	C	0.015278
62	C	-0.120535
63	O	-0.243377
64	O	-0.118661
65	O	-0.244577
66	O	-0.116904
67	C	0.048871
68	C	-0.078894
69	C	0.049339
70	C	-0.078862
71	H	0.03911
72	H	0.029292
73	H	0.036038
74	H	0.064788
75	H	0.024017
76	H	0.035643

77	H	0.045651
78	H	0.040749
79	H	0.034836
80	H	0.031241
81	H	0.045423
82	H	0.03784
83	H	0.045561
84	H	0.034908
85	H	0.036314
86	H	0.043767
87	O	-0.255256
88	C	-0.081989
89	H	0.03281
90	H	0.036992
91	H	0.021891
92	C	-0.08108
93	H	0.038575
94	H	0.036909
95	H	0.021313
96	C	0.053197
97	H	0.02689
98	C	0.05201
99	H	0.030833
100	C	-0.093152
101	H	0.026608
102	H	0.029163
103	H	0.026729
104	C	-0.077749
105	H	0.032091
106	H	0.046597
107	H	0.035695
108	C	-0.0773
109	H	0.035408
110	H	0.035013
111	H	0.044922
112	C	-0.082551
113	H	0.041085
114	H	0.026408
115	H	0.034062
116	C	0.083584
117	C	-0.004009
118	C	-0.006595
119	C	-0.030816
120	C	-0.033728
121	C	-0.040872
122	H	0.039846

123	H	0.039199
124	H	0.042756
125	C	-0.010256
126	C	0.008212
127	C	0.008196
128	C	-0.051043
129	C	-0.043647
130	C	0.012492
131	H	0.031694
132	H	0.03874
133	C	-0.008934
134	C	0.004239
135	C	0.011873
136	C	-0.057915
137	C	-0.053751
138	C	0.001762
139	H	0.027182
140	H	0.033062
141	C	-0.084425
142	H	0.035335
143	H	0.031459
144	H	0.020493
145	C	-0.0875
146	H	0.029944
147	H	0.034282
148	H	0.017241
149	C	-0.075402
150	H	0.033408
151	H	0.039128
152	H	0.035066
153	C	-0.085751
154	H	0.030272
155	H	0.02256
156	H	0.032586
157	C	-0.077179
158	H	0.033167
159	H	0.028207
160	H	0.040214
161	C	-0.084512
162	H	0.016142
163	H	0.031833
164	H	0.033086
	Tot	0.999903

Mo4 4r-2β

1	Mo	0.645807

2	N	-0.183769
3	C	-0.0194
4	N	0.027319
5	C	0.012071
6	H	0.073073
7	N	0.027315
8	C	0.012168
9	H	0.073793
10	C	0.027128
11	C	0.004561
12	C	-0.037674
13	H	0.039444
14	C	0.020847
15	C	-0.037834
16	H	0.040726
17	C	0.009011
18	C	0.023217
19	C	0.005474
20	C	-0.042649
21	H	0.038674
22	C	0.020315
23	C	-0.039611
24	H	0.041342
25	C	0.011349
26	C	-0.082303
27	H	0.033483
28	H	0.040631
29	H	0.02231
30	C	-0.074601
31	H	0.036932
32	H	0.033925
33	H	0.041701
34	C	-0.091915
35	H	0.034609
36	H	0.016097
37	H	0.034917
38	C	0.032839
39	C	0.017053
40	C	-0.037011
41	H	0.04328
42	C	-0.021062
43	H	0.04798
44	C	-0.03418
45	H	0.043674
46	C	0.0203
47	C	-0.026025

48	H	0.039333
49	C	-0.013449
50	C	0.003491
51	C	-0.084506
52	C	0.006403
53	C	-0.04951
54	C	0.012819
55	C	-0.055565
56	C	-0.076889
57	C	0.229482
58	C	0.2221
59	O	-0.107825
60	O	-0.225299
61	O	-0.109306
62	O	-0.247342
63	C	0.048886
64	C	-0.079692
65	C	0.049215
66	C	-0.078576
67	H	0.028873
68	H	0.016854
69	H	0.030688
70	H	0.033088
71	H	0.037619
72	H	0.032726
73	H	0.038258
74	H	0.045159
75	H	0.044441
76	H	0.035644
77	H	0.035701
78	H	0.03631
79	H	0.047536
80	H	0.037271
81	H	0.033136
82	H	0.043685
83	H	0.040084
84	C	-0.049318
85	C	0.008768
86	C	0.215654
87	C	0.214313
88	C	-0.02862
89	C	0.005687
90	C	-0.118251
91	O	-0.255025
92	O	-0.121855
93	O	-0.251273

94	O	-0.114425
95	C	0.048287
96	C	-0.078981
97	C	0.049547
98	C	-0.078637
99	H	0.039271
100	H	0.04445
101	H	0.040626
102	H	0.062382
103	H	0.030425
104	H	0.037726
105	H	0.045411
106	H	0.041047
107	H	0.034894
108	H	0.031179
109	H	0.044519
110	H	0.038483
111	H	0.044864
112	H	0.033956
113	H	0.036398
114	H	0.043299
115	C	-0.074113
116	H	0.036192
117	H	0.040803
118	H	0.036323
119	C	-0.078555
120	H	0.023711
121	H	0.030624
122	H	0.042911
123	C	-0.086006
124	H	0.020809
125	H	0.036676
126	H	0.034006
127	O	-0.241517
128	C	-0.078582
129	H	0.038226
130	H	0.038811
131	H	0.023208
132	C	-0.086147
133	H	0.034043
134	H	0.031858
135	H	0.021569
136	C	0.063466
137	C	-0.005067
138	C	-0.007766
139	C	-0.047654

140	C	-0.051508
141	C	0.003171
142	H	0.034709
143	H	0.034068
144	C	-0.085522
145	H	0.018144
146	H	0.031917
147	H	0.029784
148	C	-0.089792
149	H	0.029619
150	H	0.018281
151	H	0.027334
152	C	-0.076063
153	H	0.034709
154	H	0.034082
155	H	0.038303
	Tot	1.00004

Mo5 4r-2β

1	Mo	0.66177
2	N	-0.193363
3	C	-0.012843
4	N	0.02764
5	C	0.011578
6	H	0.074255
7	N	0.026907
8	C	0.011919
9	H	0.074942
10	C	0.02278
11	C	0.004149
12	C	-0.041087
13	H	0.041995
14	C	0.016969
15	C	-0.047903
16	H	0.038376
17	C	0.002849
18	C	0.019807
19	C	0.001735
20	C	-0.048041
21	H	0.036411
22	C	0.015959
23	C	-0.044661
24	H	0.042689
25	C	0.007336
26	C	-0.089258
27	H	0.032397

28	H	0.036923
29	H	0.014408
30	C	-0.073517
31	H	0.040823
32	H	0.033823
33	H	0.039493
34	C	-0.091108
35	H	0.036414
36	H	0.014306
37	H	0.037389
38	C	0.030879
39	C	0.042634
40	C	-0.029205
41	H	0.055675
42	C	-0.006701
43	H	0.057782
44	C	-0.030201
45	H	0.053769
46	C	0.041743
47	C	-0.017734
48	H	0.042669
49	C	-0.013827
50	C	0.000078
51	C	-0.079969
52	C	0.007031
53	C	-0.047162
54	C	0.009184
55	C	-0.049328
56	C	-0.077979
57	C	0.23106
58	C	0.214092
59	O	-0.10997
60	O	-0.234653
61	O	-0.108473
62	O	-0.26981
63	C	0.048669
64	C	-0.081461
65	C	0.048951
66	C	-0.079274
67	H	0.032636
68	H	0.023021
69	H	0.032223
70	H	0.035844
71	H	0.041879
72	H	0.032918
73	H	0.039481

74	H	0.045438
75	H	0.044024
76	H	0.034373
77	H	0.034558
78	H	0.032966
79	H	0.04797
80	H	0.039845
81	H	0.024775
82	H	0.039931
83	H	0.033075
84	C	-0.054191
85	C	0.009427
86	C	0.218341
87	C	0.21463
88	C	-0.033334
89	C	0.002661
90	C	-0.117605
91	O	-0.246609
92	O	-0.115423
93	O	-0.248791
94	O	-0.117827
95	C	0.047692
96	C	-0.078618
97	C	0.050194
98	C	-0.079343
99	H	0.037227
100	H	0.041934
101	H	0.043377
102	H	0.062931
103	H	0.017663
104	H	0.037207
105	H	0.045913
106	H	0.042133
107	H	0.033668
108	H	0.035396
109	H	0.045705
110	H	0.033991
111	H	0.045176
112	H	0.036812
113	H	0.036104
114	H	0.043794
115	C	-0.075598
116	H	0.04074
117	H	0.039064
118	H	0.033918
119	C	-0.081625

120	H	0.019329
121	H	0.030159
122	H	0.0427
123	C	-0.088585
124	H	0.024659
125	H	0.035785
126	H	0.031766
127	O	-0.249669
128	Cl	0.011036
129	Cl	0.022092
130	C	0.053398
131	C	0.070639
132	C	0.072868
133	C	0.078343
134	C	0.07726
135	C	0.078513
136	F	-0.079424
137	F	-0.078836
138	F	-0.081898
139	F	-0.08664
140	F	-0.077902
	Tot	1.00002

Mo19 4r-2β

1	Mo	0.621891
2	N	-0.15439
3	C	0.010124
4	N	0.03346
5	C	0.077474
6	C	-0.050876
7	H	0.03312
8	C	0.029745
9	C	0.00564
10	C	-0.035461
11	H	0.042308
12	C	-0.021798
13	C	-0.030584
14	H	0.043964
15	C	0.012762
16	C	0.039198
17	C	0.021821
18	C	-0.03126
19	H	0.045583
20	C	-0.013831
21	H	0.05215
22	C	-0.032225

23	H	0.045015
24	C	0.020802
25	C	-0.007954
26	H	0.042246
27	C	-0.01195
28	C	0.004593
29	C	-0.077666
30	C	0.013288
31	C	-0.047266
32	C	0.007246
33	C	-0.046464
34	C	-0.080316
35	C	0.229262
36	C	0.215534
37	O	-0.107893
38	O	-0.22955
39	O	-0.112695
40	O	-0.272753
41	C	0.041758
42	C	-0.07933
43	C	0.049063
44	C	-0.079585
45	H	0.034702
46	H	0.029078
47	H	0.035076
48	H	0.037061
49	H	0.041507
50	H	0.027695
51	H	0.040131
52	H	0.045521
53	H	0.044271
54	H	0.034586
55	H	0.033867
56	H	0.019467
57	H	0.041938
58	H	0.041171
59	H	0.036083
60	H	0.035798
61	H	0.036597
62	C	-0.050856
63	C	0.009054
64	C	0.21622
65	C	0.217207
66	C	-0.027889
67	C	0.023775
68	C	-0.128517

69	O	-0.245759
70	O	-0.117519
71	O	-0.239686
72	O	-0.113296
73	C	0.049558
74	C	-0.077784
75	C	0.050636
76	C	-0.079062
77	H	0.030442
78	H	0.047832
79	H	0.041191
80	H	0.058243
81	H	0.028655
82	H	0.037786
83	H	0.047025
84	H	0.042713
85	H	0.03318
86	H	0.033867
87	H	0.04582
88	H	0.039009
89	H	0.046331
90	H	0.036179
91	H	0.037219
92	H	0.044787
93	C	-0.008292
94	H	0.014704
95	C	-0.007967
96	H	0.01559
97	C	-0.013588
98	H	0.010897
99	C	-0.01078
100	H	0.017793
101	C	-0.079432
102	H	0.03017
103	H	0.029871
104	H	0.024643
105	C	-0.077099
106	H	0.030395
107	H	0.036263
108	H	0.026853
109	C	-0.077504
110	H	0.032537
111	H	0.025983
112	H	0.030564
113	C	-0.079985
114	H	0.021768

115	H	0.032886
116	H	0.027836
117	C	0.015906
118	H	0.042325
119	H	0.050787
120	C	-0.079179
121	H	0.031911
122	H	0.043429
123	H	0.036361
124	C	-0.078185
125	H	0.045979
126	H	0.035716
127	H	0.036411
128	C	-0.084583
129	H	0.037028
130	H	0.024305
131	H	0.02515
132	C	-0.079825
133	H	0.025925
134	H	0.038095
135	H	0.023959
136	Br	-0.226033
137	C	-0.079047
138	H	0.02628
139	H	0.032457
140	H	0.032487
141	C	-0.08628
142	H	0.029089
143	H	0.011317
144	H	0.029605
145	C	-0.079912
146	H	0.025621
147	H	0.029298
148	H	0.032426
149	C	-0.078428
150	H	0.032431
151	H	0.026178
152	H	0.032023
	Tot	1.000249

Mo16 4r-2β

1	Mo	0.663315
2	N	-0.16416
3	C	0.018196
4	N	0.043811
5	C	0.079133

6	C	-0.047246
7	H	0.036727
8	C	0.032589
9	C	0.006966
10	C	-0.032224
11	H	0.043545
12	C	-0.022633
13	C	-0.033529
14	H	0.043175
15	C	0.014242
16	C	0.037316
17	C	0.023508
18	C	-0.030153
19	H	0.04543
20	C	-0.013629
21	H	0.051241
22	C	-0.033354
23	H	0.044256
24	C	0.020064
25	C	-0.015052
26	H	0.042407
27	C	-0.017212
28	C	0.002409
29	C	-0.084491
30	C	0.016168
31	C	-0.047631
32	C	0.011445
33	C	-0.056315
34	C	-0.073436
35	C	0.22895
36	C	0.217
37	O	-0.113629
38	O	-0.219505
39	O	-0.106247
40	O	-0.266987
41	C	0.050039
42	C	-0.080858
43	C	0.050023
44	C	-0.078972
45	H	0.026883
46	H	0.015533
47	H	0.035586
48	H	0.034541
49	H	0.04056
50	H	0.035494
51	H	0.03899

52	H	0.045977
53	H	0.044214
54	H	0.034367
55	H	0.035241
56	H	0.037053
57	H	0.048366
58	H	0.037814
59	H	0.029333
60	H	0.042449
61	H	0.04501
62	C	-0.052689
63	C	0.009401
64	C	0.215208
65	C	0.218127
66	C	-0.030086
67	C	0.0072
68	C	-0.118572
69	O	-0.245583
70	O	-0.117188
71	O	-0.249268
72	O	-0.117035
73	C	0.049527
74	C	-0.077892
75	C	0.050247
76	C	-0.079392
77	H	0.028189
78	H	0.042013
79	H	0.043752
80	H	0.06593
81	H	0.026767
82	H	0.034737
83	H	0.046361
84	H	0.042833
85	H	0.033191
86	H	0.032371
87	H	0.045728
88	H	0.038586
89	H	0.046592
90	H	0.036058
91	H	0.036725
92	H	0.044423
93	C	-0.009894
94	H	0.015117
95	C	-0.007502
96	H	0.01844
97	O	-0.259382

98	C	-0.012364
99	H	0.010764
100	C	-0.011197
101	H	0.013254
102	C	-0.079567
103	H	0.030799
104	H	0.030067
105	H	0.025236
106	C	-0.078176
107	H	0.03037
108	H	0.035222
109	H	0.026211
110	C	-0.077636
111	H	0.031594
112	H	0.025357
113	H	0.029853
114	C	-0.079982
115	H	0.023683
116	H	0.031176
117	H	0.02836
118	C	0.050479
119	C	0.067997
120	C	0.07127
121	C	0.077698
122	C	0.078657
123	C	0.075439
124	F	-0.078856
125	F	-0.086691
126	F	-0.084624
127	F	-0.086289
128	F	-0.073172
129	C	0.016088
130	H	0.043072
131	H	0.050634
132	C	-0.079365
133	H	0.031628
134	H	0.044536
135	H	0.035902
136	C	-0.079237
137	H	0.045487
138	H	0.035944
139	H	0.036541
140	C	-0.083198
141	H	0.04236
142	H	0.025003
143	H	0.024107

144	C	-0.081042
145	H	0.020731
146	H	0.039495
147	H	0.027774
148	C	-0.082607
149	H	0.017183
150	H	0.028933
151	H	0.031913
152	C	-0.077165
153	H	0.033137
154	H	0.0274
155	H	0.032935
156	C	-0.090623
157	H	0.024967
158	H	0.009104
159	H	0.019749
160	C	-0.079739
161	H	0.026545
162	H	0.030267
163	H	0.031314
	Tot	0.999852

Mo17 4r-2β

1	Mo	0.669453
2	N	-0.164948
3	C	0.020545
4	N	0.042859
5	C	0.078768
6	C	-0.046954
7	H	0.036875
8	C	0.032088
9	C	0.007491
10	C	-0.031878
11	H	0.043914
12	C	-0.020456
13	C	-0.03284
14	H	0.043723
15	C	0.014288
16	C	0.042218
17	C	0.024181
18	C	-0.029477
19	H	0.045288
20	C	-0.015718
21	H	0.051498
22	C	-0.029416
23	H	0.049887

24	C	-0.026222
25	C	-0.017916
26	H	0.042023
27	C	-0.019012
28	C	0.001253
29	C	-0.083544
30	C	0.012159
31	C	-0.047701
32	C	0.012449
33	C	-0.055508
34	C	-0.075371
35	C	0.228782
36	C	0.215765
37	O	-0.111473
38	O	-0.22621
39	O	-0.107615
40	O	-0.266745
41	C	0.048626
42	C	-0.080214
43	C	0.049733
44	C	-0.079007
45	H	0.028477
46	H	0.014663
47	H	0.032936
48	H	0.034459
49	H	0.039413
50	H	0.032719
51	H	0.038159
52	H	0.045508
53	H	0.043995
54	H	0.03495
55	H	0.035368
56	H	0.035719
57	H	0.04855
58	H	0.038432
59	H	0.028601
60	H	0.042025
61	H	0.042918
62	C	-0.052289
63	C	0.009907
64	C	0.21647
65	C	0.217027
66	C	-0.030275
67	C	0.009576
68	C	-0.118743
69	O	-0.244554

70	O	-0.118768
71	O	-0.247713
72	O	-0.113874
73	C	0.049221
74	C	-0.078364
75	C	0.050303
76	C	-0.078419
77	H	0.028671
78	H	0.042154
79	H	0.042464
80	H	0.067682
81	H	0.028233
82	H	0.038309
83	H	0.046686
84	H	0.042227
85	H	0.034605
86	H	0.031933
87	H	0.046035
88	H	0.039074
89	H	0.046027
90	H	0.035241
91	H	0.03652
92	H	0.044455
93	C	-0.010591
94	H	0.012992
95	C	-0.007862
96	H	0.018471
97	O	-0.256004
98	C	0.028003
99	C	-0.084433
100	H	0.028575
101	H	0.014228
102	H	0.030411
103	C	-0.077237
104	H	0.033635
105	H	0.030325
106	H	0.033727
107	C	0.052373
108	C	0.069969
109	C	0.072087
110	C	0.078979
111	C	0.079651
112	C	0.076856
113	F	-0.082593
114	F	-0.085694
115	F	-0.083454

116	F	-0.0856
117	F	-0.071082
118	C	0.018517
119	H	0.043421
120	H	0.051478
121	C	-0.079548
122	H	0.032601
123	H	0.044842
124	H	0.036007
125	C	-0.077905
126	H	0.045658
127	H	0.036012
128	H	0.036036
129	C	-0.083283
130	H	0.041619
131	H	0.022945
132	H	0.023012
133	C	-0.080033
134	H	0.021891
135	H	0.040045
136	H	0.027147
137	C	-0.088978
138	H	0.009409
139	H	0.027593
140	H	0.028833
141	H	0.035317
142	C	-0.083283
143	H	0.017067
144	H	0.030012
145	H	0.032029
146	C	-0.077771
147	H	0.034121
148	H	0.027969
149	H	0.032585
150	C	-0.089287
151	H	0.027059
152	H	0.011359
153	H	0.018667
154	C	-0.079273
155	H	0.026576
156	H	0.029874
157	H	0.03166
	Tot	1.000088

Mo21 4r-2β

1	Mo	0.671341

2	N	-0.146742
3	C	0.014073
4	N	0.040996
5	C	0.080966
6	C	-0.048841
7	H	0.038068
8	C	0.032161
9	C	0.007365
10	C	-0.027515
11	H	0.044747
12	C	-0.016215
13	C	-0.032095
14	H	0.042386
15	C	0.012852
16	C	0.040637
17	C	0.023967
18	C	-0.02977
19	H	0.046586
20	C	-0.011551
21	H	0.052935
22	C	-0.030777
23	H	0.046147
24	C	0.022003
25	C	-0.006688
26	H	0.044482
27	C	-0.017814
28	C	0.00533
29	C	-0.075862
30	C	0.012623
31	C	-0.048908
32	C	0.009442
33	C	-0.052525
34	C	-0.075616
35	C	0.226532
36	C	0.214551
37	O	-0.10621
38	O	-0.236399
39	O	-0.108696
40	O	-0.271693
41	C	0.042412
42	C	-0.077816
43	C	0.048569
44	C	-0.078255
45	H	0.030956
46	H	0.018657
47	H	0.034067

48	H	0.034881
49	H	0.039716
50	H	0.033761
51	H	0.038068
52	H	0.045237
53	H	0.044396
54	H	0.035367
55	H	0.035777
56	H	0.026411
57	H	0.044468
58	H	0.039959
59	H	0.034566
60	H	0.042002
61	H	0.0433
62	C	-0.050229
63	C	0.009298
64	C	0.217157
65	C	0.217105
66	C	-0.027176
67	C	0.024595
68	C	-0.121761
69	O	-0.247016
70	O	-0.115967
71	O	-0.241574
72	O	-0.116156
73	C	0.049642
74	C	-0.077883
75	C	0.050585
76	C	-0.079204
77	H	0.038827
78	H	0.04719
79	H	0.045819
80	H	0.067304
81	H	0.027935
82	H	0.037308
83	H	0.046879
84	H	0.043181
85	H	0.03344
86	H	0.032627
87	H	0.046088
88	H	0.038549
89	H	0.04673
90	H	0.036153
91	H	0.036743
92	H	0.044439
93	C	-0.010084

94	H	0.015184
95	C	-0.012229
96	H	0.014747
97	O	-0.297077
98	C	-0.012623
99	H	0.010041
100	C	-0.011291
101	H	0.013115
102	C	-0.078737
103	H	0.030619
104	H	0.030866
105	H	0.025794
106	C	-0.077221
107	H	0.031123
108	H	0.036636
109	H	0.027234
110	C	-0.077002
111	H	0.033013
112	H	0.025143
113	H	0.031965
114	C	-0.079908
115	H	0.02252
116	H	0.03283
117	H	0.028435
118	C	0.019569
119	H	0.040994
120	H	0.052047
121	C	-0.082121
122	H	0.028443
123	H	0.043252
124	H	0.033734
125	C	-0.08225
126	H	0.044629
127	H	0.033887
128	H	0.03537
129	C	-0.076638
130	H	0.042637
131	H	0.029404
132	H	0.030558
133	C	-0.084023
134	H	0.020159
135	H	0.038738
136	H	0.025214
137	S	0.502921
138	O	-0.306682
139	O	-0.315012

140	C	0.286713
141	F	-0.083205
142	F	-0.066373
143	F	-0.085283
144	C	-0.079629
145	H	0.024816
146	H	0.028121
147	H	0.034105
148	C	-0.075354
149	H	0.032823
150	H	0.028926
151	H	0.034458
152	C	-0.082522
153	H	0.029778
154	H	0.028251
155	H	0.024787
156	C	-0.085782
157	H	0.014577
158	H	0.030376
159	H	0.029173
	Tot	1.000074

Mo6 4r-2β

1	Mo	0.667881
2	N	-0.152825
3	C	0.024886
4	N	-0.010491
5	C	0.017611
6	H	0.04372
7	N	-0.011549
8	C	0.017748
9	H	0.045648
10	C	0.016115
11	C	-0.0015
12	C	-0.046829
13	H	0.037171
14	C	0.020579
15	C	-0.036831
16	H	0.045204
17	C	0.011347
18	C	0.021112
19	C	0.002486
20	C	-0.044961
21	H	0.038237
22	C	0.01861
23	C	-0.041047

24	H	0.044913
25	C	0.011289
26	C	-0.08413
27	H	0.033539
28	H	0.03897
29	H	0.021018
30	C	-0.072931
31	H	0.044015
32	H	0.037611
33	H	0.035981
34	C	-0.093549
35	H	0.036144
36	H	0.013013
37	H	0.029123
38	C	0.044064
39	C	-0.027634
40	C	0.023374
41	C	-0.024228
42	H	0.045042
43	C	0.01877
44	C	-0.029658
45	C	-0.014469
46	H	0.041152
47	C	-0.020486
48	C	0.001542
49	C	-0.076526
50	C	0.013883
51	C	-0.047149
52	C	0.010565
53	C	-0.051683
54	C	-0.08269
55	C	0.235639
56	C	0.217056
57	O	-0.10419
58	O	-0.238659
59	O	-0.106625
60	O	-0.258547
61	C	0.047407
62	C	-0.079307
63	C	0.049104
64	C	-0.078782
65	H	0.031287
66	H	0.021751
67	H	0.036278
68	H	0.033828
69	H	0.037834

70	H	0.023619
71	H	0.038136
72	H	0.045599
73	H	0.043807
74	H	0.034831
75	H	0.035487
76	H	0.025264
77	H	0.050313
78	H	0.038708
79	H	0.026654
80	H	0.042897
81	H	0.036811
82	C	-0.057401
83	C	0.009211
84	C	0.217119
85	C	0.217554
86	C	-0.030394
87	C	0.012979
88	C	-0.128289
89	O	-0.250986
90	O	-0.117392
91	O	-0.239713
92	O	-0.114908
93	C	0.048695
94	C	-0.078126
95	C	0.050698
96	C	-0.078797
97	H	0.033286
98	H	0.040365
99	H	0.042554
100	H	0.066125
101	H	0.020242
102	H	0.035994
103	H	0.046193
104	H	0.043011
105	H	0.032602
106	H	0.034768
107	H	0.04571
108	H	0.037539
109	H	0.046581
110	H	0.036258
111	H	0.036016
112	H	0.044439
113	C	-0.073635
114	H	0.037562
115	H	0.041478

116	H	0.037706
117	C	-0.081565
118	H	0.018238
119	H	0.032384
120	H	0.041765
121	C	-0.083615
122	H	0.025623
123	H	0.041537
124	H	0.031647
125	O	-0.292268
126	S	0.517103
127	O	-0.296386
128	O	-0.297451
129	C	0.283981
130	F	-0.082936
131	F	-0.08266
132	F	-0.086134
133	C	-0.072706
134	H	0.037071
135	H	0.040616
136	H	0.041031
137	C	-0.072498
138	H	0.044316
139	H	0.039647
140	H	0.038113
141	H	0.044522
142	H	0.037326
143	H	0.050058
144	H	0.050767
	Tot	0.999972

Mo8 4r-2β

1	Mo	0.673424
2	N	-0.142277
3	C	-0.019556
4	N	0.02246
5	C	0.005369
6	H	0.0705
7	N	0.023749
8	C	0.006775
9	H	0.06977
10	C	0.046854
11	C	0.027325
12	C	-0.024951
13	H	0.047661
14	C	-0.009291

15	H	0.054102
16	C	-0.022914
17	H	0.05225
18	C	-0.019891
19	C	-0.013639
20	H	0.04203
21	C	-0.020109
22	C	0.002947
23	C	-0.075913
24	C	0.017414
25	C	-0.04711
26	C	0.009632
27	C	-0.04786
28	C	-0.083147
29	C	0.241263
30	C	0.21635
31	O	-0.094839
32	O	-0.229515
33	O	-0.105653
34	O	-0.263249
35	C	0.041599
36	C	-0.079407
37	C	0.049889
38	C	-0.078275
39	H	0.035136
40	H	0.023379
41	H	0.037396
42	H	0.036098
43	H	0.040861
44	H	0.025926
45	H	0.039476
46	H	0.04708
47	H	0.045011
48	H	0.034857
49	H	0.035918
50	H	0.016791
51	H	0.043147
52	H	0.041636
53	H	0.028324
54	H	0.040118
55	H	0.033694
56	C	-0.054006
57	C	0.009782
58	C	0.2166
59	C	0.216794
60	C	-0.02969

61	C	0.018479
62	C	-0.132469
63	O	-0.249703
64	O	-0.118305
65	O	-0.241144
66	O	-0.11395
67	C	0.049478
68	C	-0.07785
69	C	0.050956
70	C	-0.079147
71	H	0.036811
72	H	0.043852
73	H	0.043231
74	H	0.065217
75	H	0.025854
76	H	0.038123
77	H	0.047156
78	H	0.043122
79	H	0.032745
80	H	0.033602
81	H	0.046188
82	H	0.038695
83	H	0.046558
84	H	0.036164
85	H	0.036652
86	H	0.044862
87	O	-0.298037
88	S	0.51349
89	O	-0.30295
90	O	-0.298509
91	C	0.284102
92	F	-0.080072
93	F	-0.084499
94	F	-0.088791
95	C	0.028264
96	H	0.038858
97	C	-0.085634
98	H	0.011069
99	H	0.032248
100	H	0.030888
101	C	-0.08532
102	H	0.029522
103	H	0.008108
104	H	0.032423
105	C	-0.076598
106	H	0.034049

107	H	0.031209
108	H	0.035559
109	C	0.037968
110	C	-0.051743
111	C	-0.04951
112	C	-0.039589
113	C	-0.039551
114	C	-0.041956
115	H	0.025019
116	H	0.02726
117	H	0.022939
118	H	0.025997
119	H	0.026765
120	C	0.03799
121	C	-0.050169
122	C	-0.051511
123	C	-0.039588
124	C	-0.046059
125	C	-0.043601
126	H	0.021513
127	H	0.02193
128	H	0.023876
129	H	0.033198
130	H	0.033242
131	H	0.019752
132	H	0.026868
133	H	0.035412
134	H	0.035397
135	H	0.038739
136	H	0.024903
137	H	0.022702
138	H	0.02517
139	H	0.014392
140	H	0.025846
141	H	0.038184
142	H	0.025618
	Tot	1.000052

Mo9 4r-2β

1	Mo	0.665973
2	N	-0.166902
3	C	-0.016948
4	N	0.019163
5	C	0.001964
6	H	0.06933
7	N	0.023886

8	C	0.001544
9	H	0.067846
10	C	0.043563
11	C	0.022274
12	C	-0.029276
13	H	0.045288
14	C	-0.018064
15	H	0.050747
16	C	-0.027642
17	H	0.048762
18	C	-0.026384
19	C	-0.020327
20	H	0.041968
21	C	-0.014132
22	C	0.004397
23	C	-0.079414
24	C	0.007628
25	C	-0.047354
26	C	0.008391
27	C	-0.04792
28	C	-0.081102
29	C	0.23231
30	C	0.213685
31	O	-0.106334
32	O	-0.232847
33	O	-0.108561
34	O	-0.261979
35	C	0.040718
36	C	-0.075662
37	C	0.049261
38	C	-0.078838
39	H	0.035827
40	H	0.025219
41	H	0.032837
42	H	0.037355
43	H	0.0416
44	H	0.029207
45	H	0.039181
46	H	0.04586
47	H	0.04469
48	H	0.035171
49	H	0.03524
50	H	0.024044
51	H	0.033188
52	H	0.040818
53	H	0.035886

54	H	0.044621
55	H	0.031159
56	C	-0.052416
57	C	0.010253
58	C	0.216552
59	C	0.217185
60	C	-0.026996
61	C	0.017946
62	C	-0.126643
63	O	-0.248953
64	O	-0.118943
65	O	-0.243606
66	O	-0.114124
67	C	0.049502
68	C	-0.078375
69	C	0.05032
70	C	-0.078743
71	H	0.035552
72	H	0.04589
73	H	0.043541
74	H	0.065624
75	H	0.027851
76	H	0.037828
77	H	0.046292
78	H	0.042449
79	H	0.033514
80	H	0.033763
81	H	0.045634
82	H	0.038806
83	H	0.046423
84	H	0.035378
85	H	0.036444
86	H	0.044387
87	O	-0.260128
88	C	0.02763
89	H	0.034444
90	C	-0.088203
91	H	0.008842
92	H	0.031195
93	H	0.028094
94	C	-0.086521
95	H	0.027725
96	H	0.008719
97	H	0.02985
98	C	-0.077525
99	H	0.033071

100	H	0.029968
101	H	0.033618
102	C	0.034889
103	C	-0.048847
104	C	-0.048966
105	C	-0.03736
106	C	-0.039795
107	C	-0.041546
108	H	0.028127
109	H	0.028185
110	H	0.02799
111	H	0.034276
112	H	0.036522
113	C	0.040597
114	C	-0.050335
115	C	-0.051397
116	C	-0.042862
117	C	-0.043979
118	C	-0.043324
119	H	0.021625
120	H	0.021987
121	H	0.022682
122	H	0.033661
123	H	0.034122
124	H	0.020806
125	H	0.023236
126	H	0.030255
127	H	0.023567
128	H	0.034822
129	H	0.026819
130	H	0.026619
131	H	0.025114
132	H	0.018413
133	H	0.024638
134	H	0.035852
135	H	0.024408
136	C	0.05374
137	C	0.068769
138	C	0.068819
139	C	0.077449
140	C	0.080165
141	C	0.076739
142	F	-0.072693
143	F	-0.085268
144	F	-0.081297
145	F	-0.0816

146	F	-0.079595
	Tot	1.000053

Mo7 4r-2β

1	Mo	0.660457
2	N	-0.17165
3	C	0.024712
4	N	-0.011326
5	C	0.01448
6	H	0.042895
7	N	-0.009664
8	C	0.015161
9	H	0.050195
10	C	0.026393
11	C	0.005053
12	C	-0.040999
13	H	0.042042
14	C	0.018087
15	C	-0.04222
16	H	0.041421
17	C	0.008257
18	C	0.022445
19	C	0.004406
20	C	-0.042413
21	H	0.038613
22	C	0.019853
23	C	-0.039031
24	H	0.042964
25	C	0.010388
26	C	-0.084161
27	H	0.032474
28	H	0.038563
29	H	0.020682
30	C	-0.072743
31	H	0.03927
32	H	0.0358
33	H	0.040663
34	C	-0.092671
35	H	0.034323
36	H	0.014025
37	H	0.032771
38	C	0.035153
39	C	0.024485
40	C	-0.0348
41	H	0.044893
42	C	-0.018226

43	H	0.049437
44	C	-0.037773
45	H	0.04194
46	C	0.022663
47	C	-0.016342
48	H	0.042083
49	C	-0.016119
50	C	0.006376
51	C	-0.082251
52	C	0.006537
53	C	-0.048233
54	C	0.011319
55	C	-0.05159
56	C	-0.077845
57	C	0.229833
58	C	0.216983
59	O	-0.110388
60	O	-0.235371
61	O	-0.10615
62	O	-0.259601
63	C	0.046705
64	C	-0.07977
65	C	0.049448
66	C	-0.078959
67	H	0.031459
68	H	0.020255
69	H	0.02973
70	H	0.033653
71	H	0.039101
72	H	0.033115
73	H	0.038607
74	H	0.045443
75	H	0.043977
76	H	0.034896
77	H	0.035279
78	H	0.028772
79	H	0.047981
80	H	0.039028
81	H	0.02912
82	H	0.04172
83	H	0.03728
84	C	-0.053182
85	C	0.009618
86	C	0.21537
87	C	0.218947
88	C	-0.029164

89	C	0.003803
90	C	-0.123081
91	O	-0.249692
92	O	-0.116859
93	O	-0.249752
94	O	-0.117949
95	C	0.049377
96	C	-0.078558
97	C	0.049896
98	C	-0.079143
99	H	0.027209
100	H	0.042114
101	H	0.044051
102	H	0.064782
103	H	0.025181
104	H	0.036254
105	H	0.045739
106	H	0.042338
107	H	0.032859
108	H	0.03354
109	H	0.045147
110	H	0.037657
111	H	0.046705
112	H	0.036067
113	H	0.036027
114	H	0.043938
115	C	-0.072674
116	H	0.040565
117	H	0.040174
118	H	0.040932
119	C	-0.080137
120	H	0.026468
121	H	0.028544
122	H	0.043658
123	C	-0.085039
124	H	0.026983
125	H	0.037322
126	H	0.03218
127	O	-0.29308
128	H	0.048645
129	H	0.044563
130	C	-0.087991
131	H	0.033599
132	H	0.029146
133	H	0.019988
134	C	-0.078814

135	H	0.036582
136	H	0.039492
137	H	0.023581
138	C	0.017935
139	H	0.024885
140	C	0.290345
141	F	-0.103237
142	F	-0.099562
143	F	-0.094415
144	C	0.294599
145	F	-0.100957
146	F	-0.086582
147	F	-0.080349
	Tot	0.99996

Mo22 4r-2β

1	Mo	0.63569
2	N	-0.166052
3	C	0.021818
4	N	0.037211
5	C	0.080372
6	C	-0.048777
7	H	0.043907
8	C	0.036004
9	C	0.005845
10	C	-0.033109
11	H	0.04398
12	C	-0.021196
13	C	-0.0302
14	H	0.043903
15	C	0.013152
16	C	0.052205
17	C	-0.019521
18	C	-0.015699
19	H	0.054766
20	C	-0.010406
21	H	0.057527
22	C	-0.012663
23	H	0.055358
24	C	-0.01829
25	C	-0.024497
26	H	0.039468
27	C	-0.01771
28	C	0.000875
29	C	-0.083372
30	C	0.009248

31	C	-0.053104
32	C	0.008267
33	C	-0.052673
34	C	-0.077809
35	C	0.227536
36	C	0.217037
37	O	-0.101191
38	O	-0.23649
39	O	-0.10904
40	O	-0.26194
41	C	0.044661
42	C	-0.078795
43	C	0.048917
44	C	-0.078306
45	H	0.032594
46	H	0.020438
47	H	0.032057
48	H	0.034698
49	H	0.039684
50	H	0.030029
51	H	0.037714
52	H	0.046023
53	H	0.044796
54	H	0.035334
55	H	0.036273
56	H	0.024276
57	H	0.046993
58	H	0.038844
59	H	0.032596
60	H	0.039914
61	H	0.040624
62	C	-0.05399
63	C	0.009645
64	C	0.216525
65	C	0.214679
66	C	-0.025976
67	C	0.013054
68	C	-0.120302
69	O	-0.253793
70	O	-0.120455
71	O	-0.246338
72	O	-0.113271
73	C	0.049103
74	C	-0.078264
75	C	0.050289
76	C	-0.078734

77	H	0.038594
78	H	0.046877
79	H	0.044664
80	H	0.067956
81	H	0.023564
82	H	0.036959
83	H	0.046601
84	H	0.042147
85	H	0.033951
86	H	0.03175
87	H	0.04555
88	H	0.039644
89	H	0.045759
90	H	0.03497
91	H	0.03614
92	H	0.044306
93	C	-0.008387
94	H	0.001292
95	C	-0.010104
96	H	0.020506
97	C	0.019429
98	H	0.032693
99	H	0.05141
100	C	-0.081888
101	H	0.03391
102	H	0.045186
103	H	0.032132
104	C	-0.083784
105	H	0.041533
106	H	0.031317
107	H	0.032277
108	C	-0.081944
109	H	0.038155
110	H	0.020849
111	H	0.024232
112	C	-0.085395
113	H	0.014982
114	H	0.038621
115	H	0.025868
116	H	0.043917
117	C	0.302207
118	F	-0.099052
119	F	-0.090071
120	F	-0.092394
121	Br	-0.212911
122	C	-0.077173

123	H	0.035589
124	H	0.024049
125	H	0.035315
126	C	-0.080193
127	H	0.028255
128	H	0.025576
129	H	0.033297
130	C	-0.077503
131	H	0.027145
132	H	0.032719
133	H	0.033566
134	C	-0.082398
135	H	0.028392
136	H	0.017469
137	H	0.029908
	Tot	0.999996
